# Novel Alaninamide Derivatives with Drug-like Potential for Development as Antiseizure and Antinociceptive Therapies—In Vitro and In Vivo Characterization

**DOI:** 10.1021/acschemneuro.4c00013

**Published:** 2024-05-14

**Authors:** Marcin Jakubiec, Michał Abram, Mirosław Zagaja, Marta Andres-Mach, Joanna Szala-Rycaj, Gniewomir Latacz, Ewelina Honkisz-Orzechowska, Szczepan Mogilski, Monika Kubacka, Małgorzata Szafarz, Krzysztof Pociecha, Katarzyna Przejczowska-Pomierny, Elżbieta Wyska, Katarzyna Socała, Dorota Nieoczym, Bartłomiej Szulczyk, Piotr Wlaź, Cameron S. Metcalf, Karen Wilcox, Rafał M. Kamiński, Krzysztof Kamiński

**Affiliations:** † Department of Medicinal Chemistry, Faculty of Pharmacy, Jagiellonian University Medical College, Medyczna 9, 30-688 Krakow, Poland; ‡ Department of Experimental Pharmacology, Institute of Rural Health, Jaczewskiego 2, 20-950 Lublin, Poland; § Department of Technology and Biotechnology of Drugs, Faculty of Pharmacy, Jagiellonian University Medical College, Medyczna 9, 30-688 Krakow, Poland; ∥ Department Pharmacodynamics, Faculty of Pharmacy, Jagiellonian University Medical College, Medyczna 9, 30-688 Krakow, Poland; ⊥ Department of Pharmacokinetics and Physical Pharmacy, Faculty of Pharmacy, Jagiellonian University Medical College, Medyczna 9, 30-688 Krakow, Poland; # Department of Animal Physiology and Pharmacology, Institute of Biological Sciences, Faculty of Biology and Biotechnology, Maria Curie-Skłodowska University, Akademicka 19, 20-033 Lublin, Poland; ∇ Chair and Department of Pharmacotherapy and Pharmaceutical Care, Centre for Preclinical Research and Technology, Medical University of Warsaw, Banacha 1B, 02-097 Warsaw, Poland; ○ Department of Pharmacology and Toxicology, University of Utah, Salt Lake City, Utah 84112, United States

**Keywords:** hybrid molecules, antiseizure activity, antinociceptive activity, epilepsy, neuropathic pain, ADME-Tox properties

## Abstract

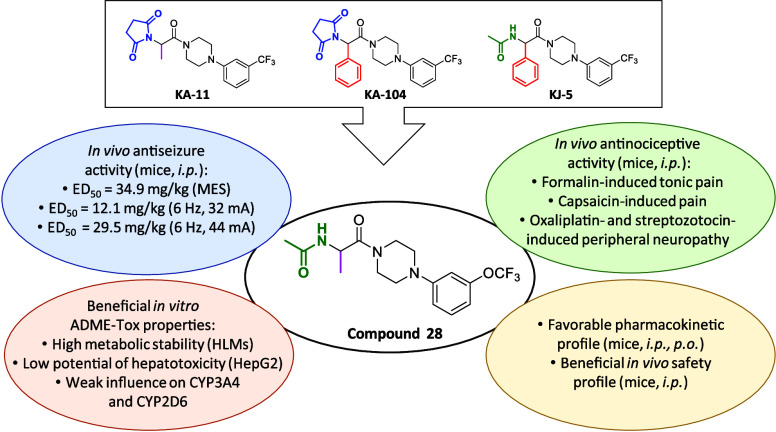

In the present study, a series of original alaninamide derivatives have been designed applying a combinatorial chemistry approach, synthesized, and characterized in the *in vivo* and *in vitro* assays. The obtained molecules showed potent and broad-spectrum activity in basic seizure models, namely, the maximal electroshock (MES) test, the 6 Hz (32 mA) seizure model, and notably, the 6 Hz (44 mA) model of pharmacoresistant seizures. Most potent compounds **26** and **28** displayed the following pharmacological values: ED_50_ = 64.3 mg/kg (MES), ED_50_ = 15.6 mg/kg (6 Hz, 32 mA), ED_50_ = 29.9 mg/kg (6 Hz, 44 mA), and ED_50_ = 34.9 mg/kg (MES), ED_50_ = 12.1 mg/kg (6 Hz, 32 mA), ED_50_ = 29.5 mg/kg (6 Hz, 44 mA), respectively. Additionally, **26** and **28** were effective in the *iv*PTZ seizure threshold test and had no influence on the grip strength. Moreover, lead compound **28** was tested in the PTZ-induced kindling model, and then, its influence on glutamate and GABA levels in the hippocampus and cortex was evaluated by the high-performance liquid chromatography (HPLC) method. In addition, **28** revealed potent efficacy in formalin-induced tonic pain, capsaicin-induced pain, and oxaliplatin- and streptozotocin-induced peripheral neuropathy. Pharmacokinetic studies and *in vitro* ADME-Tox data proved favorable drug-like properties of **28**. The patch-clamp recordings in rat cortical neurons showed that **28** at a concentration of 10 μM significantly inhibited fast sodium currents. Therefore, **28** seems to be an interesting candidate for future preclinical development in epilepsy and pain indications.

## 1 Introduction

Epilepsy is a chronic neurological disease characterized by an enduring (i.e., persisting) predisposition to generate unprovoked seizures and affects people of all ages, races, social classes, and geographical locations.^1^ A multifactorial pathogenesis of epilepsy complicates therapeutic approaches, and currently available antiseizure medications (ASMs) display limited efficacy. In fact, approximately 30% of patients with epilepsy have drug-resistant epilepsy (DRE) and do not respond to ASMs.^2^ DRE is a serious condition as it is associated with a risk of sudden unexpected death in epilepsy (SUDEP), as well as psychiatric, psychosocial, and medical complications, having a profound influence on the overall quality of life.^3^

One of the promising therapeutic strategies in DRE involves development of multimodal (multitarget/multifunctional) molecules modulating different molecular targets and thereby overcoming the issues associated with polytherapy, which is often used in this condition. Polytherapy with ASMs increases the risk of drug–drug interactions, as well as may cause multiple adverse effects, and thus suboptimal adherence.^4,5^ Such multitarget molecules could be designed as hybrid or chimeric congeners, which incorporate multiple pharmacophores into a single molecule, often with a broader and synergic mechanism of action. The trend to transition from the single target to the multitarget concept is observed in not only epilepsy but also many neurodegenerative diseases (i.e., Alzheimer’s and Parkinson’s), depression, diabetes, metabolic and inflammatory diseases, cancer, as well as other neurological disorders such as neuropathic pain.^6−13^

**Figure 1:**
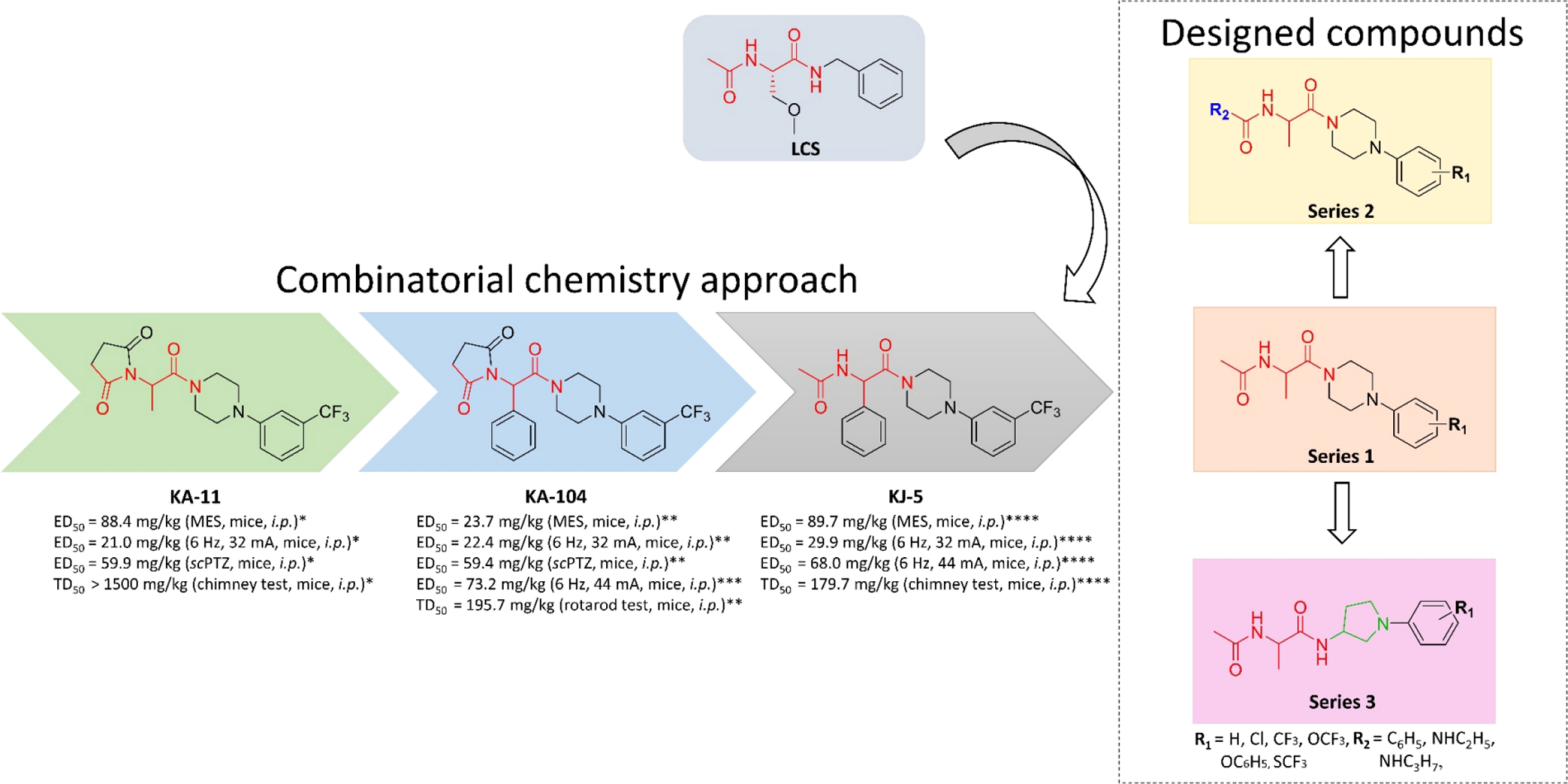
Development process yielding in broad-spectrum antiseizure compounds **KA-11**, **KA-104**, and **KJ-5** described previously, as well as a general structure of new hybrid molecules reported herein. * Data from ref (14) (see compound **11**). ** Data from ref (16) (see compound **22**). *** Data from ref (17). **** Data from ref (18) (see compound **53**).

Adhering to the concept of multitargeted strategy, we have recently developed several novel series of hybrid compounds based on the pyrrolidine-2,5-dione scaffold, exemplified by the most promising compounds (**KA-11** and **KA-104**), as well as their acyclic analogues such as **KJ-5** (Figure 1).^14−18^ Consequently, these hybrid molecules demonstrate potent and broad-spectrum antiseizure activity in a range of preclinical seizure models, including the maximal electroshock (MES), 6 Hz (32/44 mA), and/or the subcutaneous pentylenetetrazol (*sc*PTZ). It should be stressed that all of the aforementioned compounds belong to a class of modified amino acid derivatives with α-alanine (**KA-11**) or phenylglycine (**KA-104** and **KJ-5**) as core fragments. As shown in Figure 1, the exchange of the α-alanine residue (**KA-11**) to phenylglycine (**KA-104**) caused a substantial increase in activity against MES, whereas further modification and degradation of the pyrrolidine-2,5-dione ring to the acetamide moiety resulted in compound **KJ-5**, which was most effective in the 6 Hz (44 mA) seizure model of DRE. Despite the less potent activity of **KA-11** than that of **KA-104** in the MES test, this compound showed limited impact on locomotor performance even at very high doses (TD_50_ > 1500 mg/kg). Building on the success of these chemotypes, namely, **KA-11**, **KA-104**, and **KJ-5**,^15,17^ we now focused on a library of alaninamide derivatives designed as hybrids of **KA-11** and **KJ-5** (Figure 1, Series 1). It should also be emphasized that the 2-acetamidopropanamide fragment (marked in red) is structurally similar to a fragment of lacosamide (LCS), an approved ASM with well-established activity in the preclinical models. Thus, we hypothesize herein that the proposed structural modifications may potentially further improve the antiseizure activity of such compounds as well as retain or improve antinociceptive properties previously reported for the predecessor compounds. Aiming to broaden the novel chemical space, as well as following the above-described structure–activity relationships, we designed two additional modifications: (i) introduction of an additional aromatic ring at the acetamide moiety (to increase lipophilicity and potentially improve blood–brain barrier (BBB) permeability) together with the substitution of acetamide with the urea fragment (Figure 1, Series 2); (ii) bioisosteric replacement of the piperazine ring into the pyrrolidin-3-amine moiety (Figure 1, Series 3). Notably, both urea and pyrrolidin-3-amine fragments are present in a number of TRPV1 channel antagonists (i.e., BCTC or SB-705498) characterized by potent analgesic activity in preclinical studies.^19−25^ Further, we hypothesized that these structural modifications may enhance the interaction of compounds described herein with TRPV1, which was one of the most important molecular targets for **KA-104**.^16^ Finally, due to the structural similarities of the newly designed compounds to the previously reported **KA-11**, **KA-104**, and **KJ-5**, we assumed that these molecules may also be characterized by a multimodal mechanism of action, including interaction with Na_v_x, Ca_v_1.2, and TRPV1 channels, translating into broad antiseizure and antinociceptive activity.

The present integrated drug discovery studies involved design, synthesis, *in vivo* determination of antiseizure and antinociceptive activities, and evaluation of the pharmacokinetic profiles. In addition, the most effective compounds were further characterized in the mechanism of action studies, such as *in vitro* binding/functional assays. Finally, preliminary ADME-Tox studies were performed using a range of standard assays such as membrane permeability, plasma protein binding, metabolic stability, hepatotoxicity, or influence on the function of cytochrome P450 isoforms, CYP3A4 and CYP2D6.

## 2 Results and Discussion

### 2.1 *In Silico* Studies

All compounds were designed following drug-like physicochemical properties based on Lipinski (RO5) and Veber rules using SwissAdme software,^26,27^ as well as the central nervous system multiparameter optimization (CNS MPO) algorithm.^28^ The physicochemical properties of all obtained compounds are shown in Table 1.

**Table 1:** Parameters Calculated According to the Lipinski Rule, Veber Rule, and CNS MPO

			Lipinski’s rule				Veber’s rule		
cmpd	R_1_	R_2_	MW ≤ 500	log *P* ≤ 5	HBD^a^ ≤ 5	HBA^b^ ≤ 10	NBR^c^ ≤ 10	TPSA^d^ ≤ 140 Å^2^	CNS MPO^e^
**21**	H		275.35	1.13	1	2	5	52.65	5.75
**22**	3-Cl		309.79	1.64	1	2	5	52.65	5.75
**23**	4-Cl		309.79	1.64	1	2	5	52.65	5.75
**24**	3,4-diCl		344.24	2.16	1	2	5	52.65	5.75
**25**	3,5-diCl		344.24	2.16	1	2	5	52.65	5.75
**26**	3-CF_3_		343.34	2.19	1	5	6	52.65	5.75
**27**	4-CF_3_		343.34	2.19	1	5	6	52.65	5.75
**28**	3-OCF_3_		359.34	2.02	1	6	7	61.88	5.73
**29**	3-OC_6_H_5_		367.44	2.32	1	3	7	61.88	5.64
**30**	3-SCF_3_		375.41	2.53	1	5	7	77.95	5.35
**31**	3-Cl	C_6_H_5_	371.86	2.78	1	2	6	52.65	5.09
**32**	3-CF_3_	C_6_H_5_	405.41	3.29	1	5	7	52.65	4.57
**33**	3-Cl	NHC_2_H_5_	338.83	1.71	2	2	7	64.68	5.50
**34**	3-CF_3_	NHC_2_H_5_	372.39	2.21	2	5	8	64.68	5.41
**35**	3-Cl	NHC_3_H_7_	352.86	1.91	2	2	7	64.68	5.50
**36**	3-CF_3_	NHC_3_H_7_	386.41	2.56	2	5	8	64.68	5.23
**45**	H		275.35	1.22	2	2	6	61.44	5.50
**46**	3-CF_3_		343.34	2.33	2	5	7	61.44	5.50
**47**	3-OCF_3_		359.34	2.11	2	6	8	70.67	5.50
**48**	3-SCF_3_		375.41	2.70	2	5	8	86.74	5.15

a HBD: number of hydrogen bond donors.

b HBA: number of hydrogen bond acceptors.

c NBR: number of rotatable bonds.

d TPSA: topological polar surface area.

e CNS MPO: central nervous system multiparameter optimization scores were calculated using Instant JChem 21.4.0 software (ChemAxon).

RO5 and Veber rules are often used in the drug discovery process to evaluate drug-like physicochemical properties suitable for oral administration in humans, which are as follows: molecular weight (MW) ≤ 500 Da, lipophilicity (log *P*) ≤ 5, number of hydrogen bond donors (HBD) ≤ 5, number of hydrogen bond acceptors (HBA) ≤ 10, for RO5, whereas Veber’s rule involves number of rotatable bonds (*N*) ≤ 10 and topological polar surface area (TPSA) ≤ 140 Å^2^. Importantly, all compounds described in the present study comply with the above-mentioned rules. The CNS MPO score (Instant JChem by ChemAxon software version 21.4.0), which predicts blood–brain barrier (BBB) permeability, was also calculated for all of the compounds. CNS MPO approach assumptions involve six key physicochemical properties: lipophilicity (*C* log *P*), calculated distribution coefficient at pH 7.4 (*C* log *D*), molecular weight (MW), TPSA, HBDs, and most basic center (p*K*_a_). Each parameter has values between 0 and 1; thus, the collective score ranges from 0 to 6 (a higher CNS MPO score is more desirable). The scores ≥4.0 are widely used as a cutoff to select compounds for hit finding in CNS therapeutic area drug discovery programs. It should be stressed here that all of the designed compounds had scores ≥4. Importantly, most of the compounds (**21**–**31**, **33**–**36**, and **45**–**48**) in the set had CNS MPO scores greater than 5, which indicates optimal properties for penetration through the BBB. Only one molecule, namely **32**, had a score <5 (4.57), i.e., likely due to a relatively high MW.

### 2.2 Chemistry

In chemical studies, a library of 20 original compounds grouped into 3 series was obtained. It should be emphasized that due to ethical issues (animal testing as the first line of screening, which is characteristic for identification of new ASMs), the substitution mode of the phenylpiperazine moiety was restricted only to atoms/groups favorable for antiseizure activity as identified in the previous studies, e.g., 3-Cl, 4-Cl, 3,4-diCl, 3,5-diCl, 3-CF_3_, 4-CF_3_, 3-OCF_3_, 3-OC_6_H_5_, and 3-SCF_3_.^14,16,18,29^

Compounds from Series 1 (**21**–**30**) were synthesized by applying the multistep procedure that also involved preparation of selected noncommercial amines (**A5**–**A8**, for details, see the Supporting Information (SI)). The noncommercial 4-arylpiperazines (**A5**–**A8**) were synthesized according to Scheme S1 in a two-step reaction. First, Boc-protected intermediates **A1**–**A4** were obtained by the reaction of aryl bromides with 1-Boc-piperazine in the Buchwald–Hartwig reaction in the nitrogen atmosphere.^30^ The removal of the Boc group in acid conditions (TFA, trifluoroacetic acid), followed by neutralization with 25% ammonium hydroxide, yielded the desired 4-arylpiperazine derivatives **A5**–**A8**, which were used for the next reactions without purification.

**Scheme 1:**
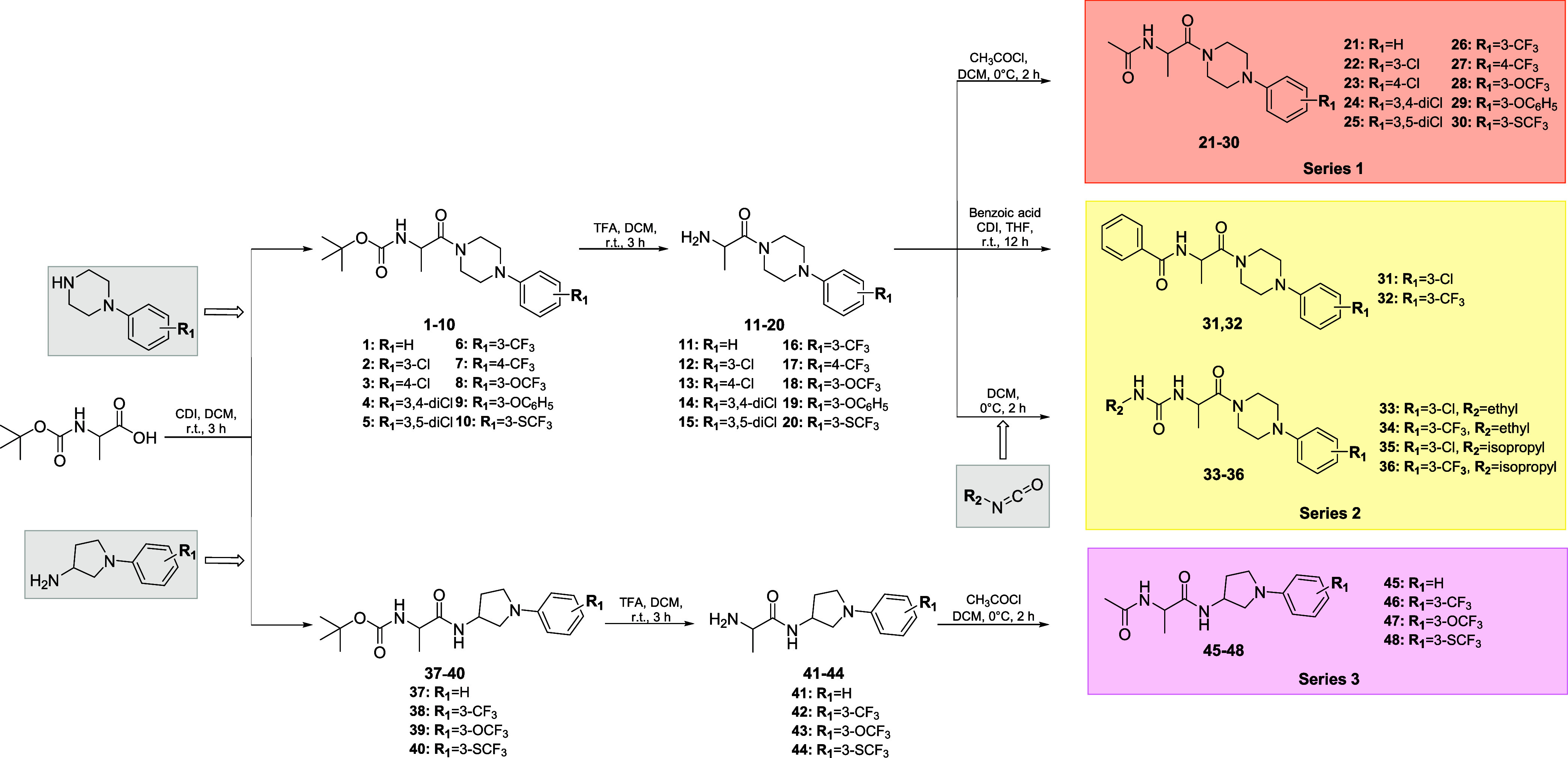
Synthesis of Intermediates and Final Compounds of Series 1 (**21**–**30**), Series 2 (**31**–**36**), and Series 3 (**45**–**48**)

The final compounds from Series 1 (**21**–**30**) were synthesized according to Scheme 1. First, the condensation reaction of appropriate 4-arylpiperazine derivatives (commercial or noncommercial, **A5**–**A8**), with Boc-dl-alanine in the presence of carbonyldiimidazole (CDI) yielded Boc-protected intermediates **1**–**10**. In the next step, as a result of the removal of the Boc protecting group by addition of TFA, the amine derivatives (**11**–**20**) were obtained. The target compounds (**21**–**30**) were obtained in the acylation reaction of amine derivatives (**11**–**20**) by acetyl chloride.

Continuing chemical studies, we proposed targeted modifications of compounds from Series 1 (**22** and **26**) with confirmed antiseizure activity by the exchange of the acetyl fragment to the benzoyl moiety (Series 2, compounds **31** and **32**), as well as its conversion to the urea moiety modified with alkyl substituents (Series 2, compounds **33**–**36**). Therefore, compounds of Series 2 were synthesized in a similar manner as for Series 1, applying benzoic acid, CDI as a coupling agent, and intermediate **12** (3-Cl) or **16** (3-CF_3_) as the amine component. The same amines (**12** and **16**) underwent condensation reaction with appropriately substituted isocyanates to give urea derivatives **33**–**36**.

Finally, with the aim of examining the influence of the piperazine moiety on antiseizure activity for the unsubstituent derivative (**21**) and the most potent antiseizure compounds (**26**, **28**, and **30**) from Series 1, respective pyrrolidin-3-amine bioisosteres were synthesized (Series 3). At the first step of the synthetic procedure, noncommercial 1-phenylpyrrolidin-3-amine derivatives (**A13**–**A16**) were synthesized in a two-step reaction, according to Scheme S2. First, Boc-protected intermediates **A9**–**A12** were obtained by the reaction of aryl bromides with *N*-Boc-3-amine-pyrrolidine in the Buchwald–Hartwig reaction in the nitrogen atmosphere. The removal of the Boc group with TFA was followed by neutralization with 25% ammonium hydroxide and yielded the desired 1-phenylpyrrolidin-3-amine derivatives **A13**–**A16**, which were used for the next reactions without purification (for details, see SI).

The final compounds of Series 3 (**45**–**48**) were synthesized in a similar chemical procedure as for Series 1 using as substrates respective noncommercially available 1-phenylpyrrolidin-3-amine derivatives. The crude products of all three final series (**21**–**36**, **45**–**48**) were purified by column chromatography. The desired compounds were obtained as white solids, followed by a wash-up with diethyl ether. For details, see Scheme 1.

All final compounds **21**–**30** (Series 1), **31**–**36** (Series 2), and **45**–**48** (Series 3) were obtained in good yields (>84%). The structures of noncommercial 4-phenylpiperazines, as well as 1-phenylpyrrolidin-3-amines and final molecules, were confirmed by ^1^H NMR and/or ^13^C NMR spectra analyses. Moreover, for all compounds, the liquid chromatography-mass spectrometry (LC-MS) spectra were also obtained, and for the most potent antiseizure compounds (**22**, **24**–**26**, **28**–**30**), high-resolution mass spectrometry (HRMS) analysis was carried out. The purity of final compounds determined by the ultraperformance liquid chromatography (UPLC) method was ≥97.5%. The physicochemical and spectral data for intermediates and final compounds are summarized in the Section 4. The procedure for the synthesis of starting amines **A1**–**A16** and analytical data are described in the SI.

### 2.3 Antiseizure Activity

Identification and profiling of new ASM candidate compounds are still largely based on predictive animal models of seizures/epilepsy, while a range of *in silico* and *in vitro* approaches (i.e., molecular modeling or high throughput screening, etc.) are increasingly used in the early stages of ASM discovery.^31^ The Epilepsy Therapy Screening Program (ETSP) of the National Institute of Neurological Disorders and Stroke (NIH, Bethesda, MD) has enabled the discovery of several approved ASMs by focusing on the *in vivo* testing of compounds synthesized by both academic laboratories and industry.^32^ The ETSP provides a broad panel of seizure tests (ca. 30 rodent models), but a subset of them is used for the initial screening of new compounds with potential antiseizure activity. The routinely used seizure models include: (i) the MES test, which is an experimental model of tonic–clonic seizures, (ii) the 6 Hz model (32 mA) of focal seizures, and (iii) the *sc*PTZ model of generalized absence or myoclonic seizures.^33,34^ Consequently, in the current studies, all final compounds **21**–**36** and **45**–**48** were initially studied in the MES and the 6 Hz (32 mA) seizure models after intraperitoneal (*i.p.*) administration at a screening dose of 100 mg/kg in mice at a time point of 0.5 h (the screening group consisted of four mice, and the results obtained are summarized in Table S1).

According to the MES screening data, the maximal 100% antiseizure protection (four mice out of four tested) was demonstrated for **22**, **26**, and **28**–**30**. Still satisfactory 75% protection (three mice out of four tested) was demonstrated for compounds **24** and **25**. Other molecules, **27** and **36**, showed either limited protection (25%) or lack of activity in the MES test. Thus, the results from the MES indicated that the highest antiseizure activity was demonstrated by compounds from Series 1 containing electron-withdrawing atom/group in the phenylpiperazine moiety (especially at 3-position), namely, **22** (3-Cl), **26** (3-CF_3_), **28** (3-OCF_3_), **29** (3-O_6_H_5_), and **30** (3-SCF_3_), while respective benzoyl or urea (Series 2) analogues rendered the compound ineffective. Replacing the piperazine ring with the bioisosteric pyrrolidin-3-amine moiety also caused a decrease of antiseizure activity.

The next step of *in vivo* screening involved testing of all compounds in the 6 Hz (32 mA) model to demonstrate a broad spectrum of antiseizure activity. Consequently, the obtained data showed distinctly more potent protection against focal seizures in this model. Maximal (100% efficacy) was demonstrated with **24**, **26**, **28**, and **30** (Series 1), which was in general in line with MES test data. Weaker, but satisfying, activity was provided by **22**, **25**, **29** (75% protection), **33**–**35**, **47**, and **48** (50% protection). Limited activity (25% protection) was observed for **21**, **32**, and **36**. Notably, only five compounds were devoid of any protection in the 6 Hz (32 mA) test. Overall, compounds described herein were more potent in the aforementioned seizure model versus MES, and in each series, we were able to identify molecules producing at least 50% seizure protection.

Finally, selected compounds showing broad antiseizure protection, being the most effective substances in the MES and 6 Hz (32 mA) tests (efficacy >50% in both models), were also screened in the *sc*PTZ test. Surprisingly, the highest 75% protection was demonstrated for 3,4-diCl derivative **24**. In this test, **25** and **30** displayed 50% protection, whereas **22**, **26**, and **28** protected only 25% of the animals. Only **29** showed no activity at a dose of 100 mg/kg.

In summary, based on the screening data, the most potent antiseizure activity was observed for compounds containing the acetyl moiety (Series 1) and especially for molecules containing electron-withdrawing groups at the 3-position of the phenylpiperazine fragment, such as –Cl, –CF_3_, –OCF_3_, –OC_6_H_5_, –SCF_3_, or disubstituted derivatives, 3,4- and 3,5-diCl. Replacing the acetyl fragment with a benzoyl group or urea fragment (Series 2), as well as bioisosteric replacement of piperazine with the pyrrolidin-3-amine moiety (Series 3), caused a decrease of activity. Thus, it may be concluded that only compounds from Series 1 showed expected potent and broad-spectrum antiseizure activity.

Based on the above screening data, in the next step of pharmacological studies, we determined the median effective doses (ED_50_) for all of the compounds protecting at least 75% of mice in each seizure model (MES or/and 6 Hz [32 mA] or/and *sc*PTZ). Moreover, the CNS tolerability of the compounds was quantified as the median toxic doses (TD_50_) in the chimney test 0.5 h post *i.p.* administration. These parameters were then used to calculate the protective indexes (PIs, PI = TD_50_/ED_50_), which describe the therapeutics window of potential drug candidates. The ED_50_, TD_50_, and PI values for the tested compounds together with data of their chemical prototypes **KA-11** and **KJ-5** and reference ASMs are summarized in Table 2.

**Table 2:** ED_50_, TD_50_, and PI Values in Mice after *i.p.* Administration of the Newly Obtained Compounds, Chemical Prototypes, and Reference ASMs in Mice^a^

cmpd	PT (h)^b^	ED_50_ MES (mg/kg)	ED_50_ 6 Hz (32 mA) (mg/kg)	TD_50_ (mg/kg)	PI (TD_50_/ED_50_)
**22**	0.5	122.2 (104–143.3)	77.3 (58.9–101.5)	213.2* (242.0–297.1)	1.7 (MES) 2.8 (6 Hz)
**24**	0.5	>150	41.4 (23.5–73.0)	138.9* (123.2–156.7)	3.4 (6 Hz)
**25**	0.5	52.9 (40.3–69.4)	56.1 (35.5–88.8)	199.6* (160.3–248.5)	3.8 (MES) 3.6 (6 Hz)
**26**	**0.5**	**64.3** (53.0–78.0)	**15.6** (7.1–34.4)	172.3* (140.3–211.6)	**2.7 (MES) 11.0** (6 Hz)
**28**	**0.5**	**34.9** (29.2–41.7)	**12.1** (6.8–21.8)	99.4* (83.0–119.0)	**2.8 (MES) 8.2** (6 Hz)
**29**	0.5	39.9 (32.1–49.7)	41.2 (29.9–56.8)	108.6* (100.6–117.3)	2.7 (MES) 2.6 (6 Hz)
**30**	0.5	45.3 (39.0–52.5)	39.1 (31.6–48.4)	134.8* (104.8–173.3)	3.0 (MES) 3.4 (6 Hz)
**KA-11**^c^	0.5	88.4 (73.2–106.8)	21.0 (12.4–35.7)	>1500*	>17.0 (MES) > 71.4 (6 Hz)
**KJ-5**^d^	0.5	89.7 (71.4–112.8)	29.9 (20.1–44.4)	179.7* (161.0–200.5)	2.0 (MES) 6.0 (6 Hz)
**LCS**^e^	0.5	9.2 (8.5–10.0)	5.3 (3.5–7.8)	46.2** (44.5–48.0)	5.0 (MES) 8.8 (6 Hz)
**VPA**^e^	0.5	252.7 (220.1–290.2)	130.6 (117.6–145.2)	430.7** (407.9–454.9)	1.7 (MES) 3.3 (6 Hz)

a The data for the most potent compounds **26** and **28** have been bolded for better visualization. Results are represented as mean ± standard error of the mean (SEM) at a 95% confidence limit determined by probit analysis. Acute neurological deficit (TD_50_) determined in the *chimney test or **rotarod test.

b Pretreatment time.

c Data for **KA-11**, see compound **11** in ref (14).

d Data for **KJ-5**, see compound **53** in ref (18).

e Reference ASMs: lacosamide (LCS) and valproic acid (VPA) tested in the same conditions; data from ref (16).

As expected, based on the initial screening results, the most potent protection in the MES and 6 Hz (32 mA) seizure tests was shown for **25**, **26**, **28**, **29**, and **30**. Weaker activity in those two seizure models was noted for **22**, whereas **24** demonstrated activity exclusively in the 6 Hz (32 mA) model (Table 2). In the *sc*PTZ test, only **24** produced dose-dependent protection against seizures (ED_50_ = 115.6 [100.6–132.8] mg/kg (data not shown in Table 2)); however, it was less active than its chemical prototype **KA-11** (ED_50_ = 59.9 [52.5–68.3] mg/kg, mice, *i.p.*).^14^ Relatively modest protection in the chemically induced seizure model by compounds reported herein indicates that the pyrrolidine-2,5-dione ring seems to be crucial for activity in the *sc*PTZ test, as it was described by our team in the previous studies.^14,16,29^ Furthermore, the decrease of efficacy in the *sc*PTZ test may also result from the presence of the 2-acetamidopropanamide fragment (marked in red in Figure 1) with structural resemblance to LCS, which is inactive in the *sc*PTZ model.

Among the aforementioned compounds, the most promising antiseizure properties and tolerability profile were revealed by compounds **26** (3-CF_3_) and **28** (3-OCF_3_), which showed a slightly better therapeutic window (PI values), especially in the 6 Hz (32 mA) seizure model than their close analogue **30** (3-SCF_3_) and other antiseizure compounds identified herein. Chemical predecessor **KA-11** showed weaker protection in the MES test and only slightly less potent activity in the 6 Hz (32 mA) seizure model. Unfortunately, the improvement of the antiseizure activity of **26** and **28** was accompanied by the simultaneous increase of neurotoxicity in the chimney test that resulted in the worsening of PIs compared to **KA-11**. In comparison to acetyl precursor **KJ-5**, compounds **26** and **28** reported herein showed more potent activity in the MES test and distinctly better potency (2-fold) in the 6 Hz (32 mA) test. It should also be emphasized that **26** and **28** demonstrated more potent antiseizure activity in both mentioned tests, as well as better PIs in each seizure model than VPA, which is still recognized as first-line ASM used for the treatment of different types of epilepsies. Compounds **26** and **28** exhibited lower potency in the MES and 6 Hz (32 mA) seizure models and also a lower therapeutic window in the MES test compared to LCS, but on the other hand, they showed better (**26**) or similar (**28**) PIs values compared to LCS in the 6 Hz (32 mA) seizure model.

Based on the promising pharmacological data for **26** and **28**, and especially potent protection in the 6 Hz (32 mA) seizure model, both compounds were tested subsequently in the 6 Hz seizure model, applying a higher current intensity of 44 mA (Table 3). Importantly, this test is recognized as one of the key animal models of pharmacoresistant seizures, utilized in the early stage of new ASMs’ development.

**Table 3:** Antiseizure Activity of **26**, **28**, Chemical Prototype **KJ-5**, and Reference ASMs in the 6 Hz (44 mA) Seizures in Mice *i.p.*^a^

cmpd	PT (h)^b^	ED_50_ 6 Hz (44 mA) (mg/kg)	TD_50_ (mg/kg)	PI (TD_50_/ED_50_)
**26**	0.5	29.9 (19.8–44.9)	172.3* (140.3–211.6)	5.8
**28**	0.5	29.5 (20.4–42.5)	99.4* (83.0–119.0)	3.4
**KJ-5**^c^	0.5	68.0 (57.2–80.9)	179.7* (161.0–200.5)	2.6
**LCS**^d^	0.5	6.9 (5.4–8.6)	46.2** (44.5–48.0)	6.7
**VPA**^d^	0.5	183.1 (143.5–233.7)	430.7** (407.9–454.9)	2.3

a Results are represented as mean ± SEM at a 95% confidence limit determined by probit analysis. Acute neurological deficit (TD_50_) determined in the *chimney test or the **rotarod test.

b Pretreatment time.

c Data for **KJ-5** from ref (18).

d Reference ASMs: lacosamide (LCS) and valproic acid (VPA) tested in the same conditions; data from ref (16).

The data obtained revealed potent protection for both tested compounds **26** and **28**. Importantly, these molecules were clearly more effective (>2-fold) than their chemical prototype **KJ-5**. Furthermore, it should be stressed that **26** and **28** were more potent and showed higher PI values than VPA. In this model, LCS, one of the chemical precursors for **26** and **28**, revealed excellent efficacy and the best PI value from all tested substances.

In conclusion, the most potent antiseizure properties among tested compounds were observed for **26** and **28**. These molecules show a broad spectrum of protection and were effective in the MES and 6 Hz (32 mA) tests and notably in the 6 Hz (44 mA) seizure model of DRE. The comparison of antiseizure potency and tolerability data for **26** and **28** to their chemical precursors, namely, **KA-11** and **KJ-5**, indicated that the pyrrolidine-2,5-dione ring (see **KA-11**) is essential for activity in the *sc*PTZ model,^14,16,29^ while its degradation to the acyclic acetyl fragment improves the activity of distinctly electrically induced model of seizures. A similar observation (including also activity in the 6 Hz [44 mA] model) was noted in the case of the exchange of the core phenylglycine fragment (see **KJ-5**) into the alanine residue (**26** and **28**). Unfortunately, structural modifications described in the current studies caused an increase of neurological toxicity as tested in the chimney test (especially compared to **KA-11**). Apart from this and taking into consideration more potent antiseizure activity and better tolerability profile of **26** and **28** compared to VPA, it can be postulated that these molecules may be promising candidates for further preclinical development.

### 2.4 Effect on the Seizure Threshold in the *iv*PTZ Test in Mice

**Figure 2:**
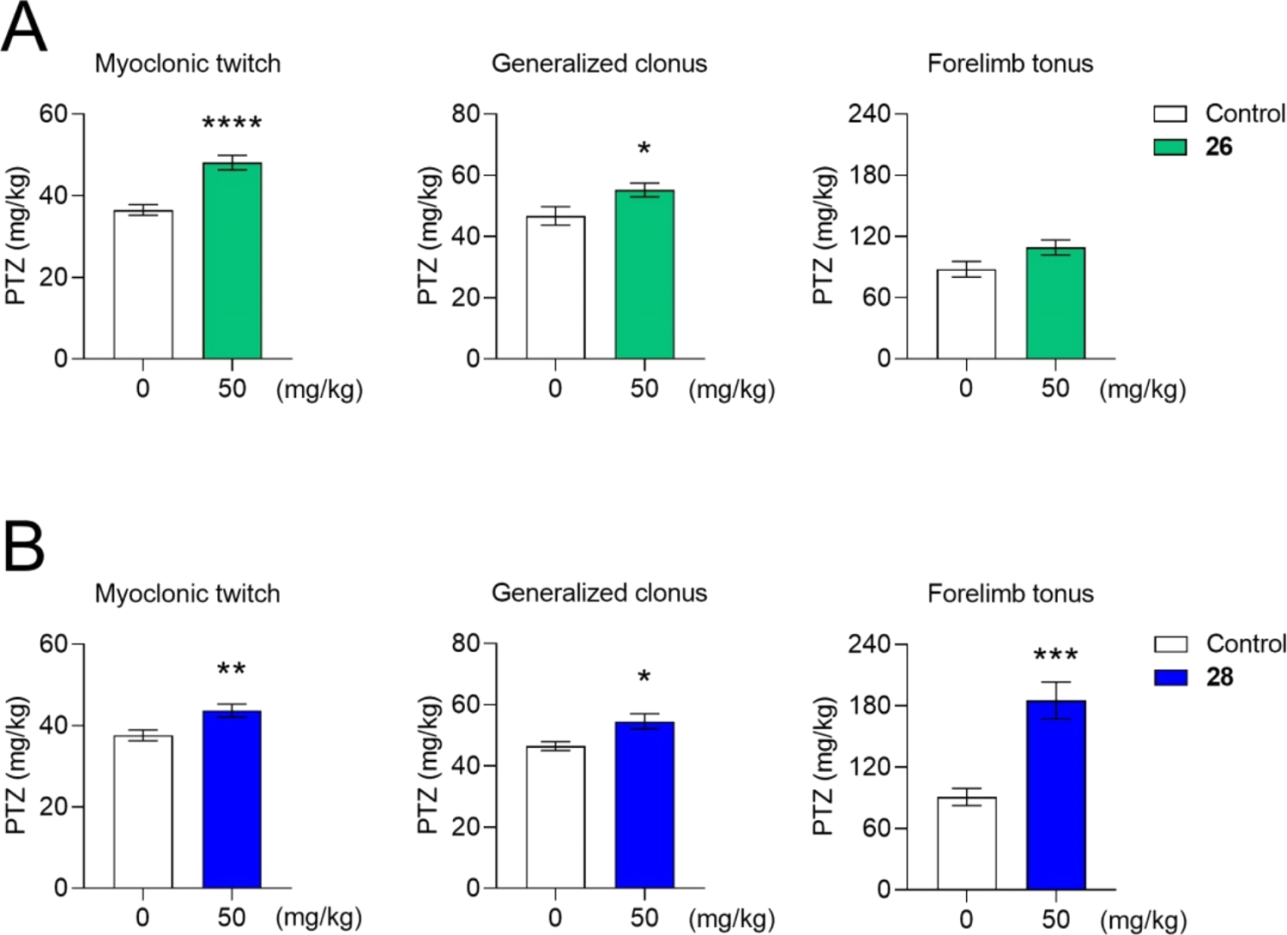
Acute effects of compounds **26** (A) and **28** (B) on seizure thresholds in the timed *iv*PTZ seizure test in mice. Both compounds were administered *i.p.* at a dose of 50 mg/kg, 30 min before the test. Control animals received vehicle. Data are presented as means (mg/kg PTZ) ± SEM (*n* = 9–13 animals). The statistical significance was evaluated by a Student’s *t-*test: *p < 0.05, **p < 0.01, ***p < 0.001, and ****p < 0.0001, as compared to the vehicle-treated group (GraphPad Prism 8).

The timed intravenous (*iv*) PTZ-induced seizure test was used to characterize the acute effect of compounds **26** and **28** on the thresholds for myoclonic, clonic, and tonic seizure in mice (Figure 2). It is noteworthy that the *iv*PTZ seizure test is a very sensitive method for determining the compounds’ effects on seizure threshold in rodents.^35^ Both compounds administered at a dose of 50 mg/kg significantly raised the seizure thresholds for the first myoclonic twitch (*p* < 0.0001 and *p* < 0.01 for compounds **26** and **28**, respectively) and generalized clonic seizure with loss of righting reflex (*p* < 0.05 for both compounds). In the *iv*PTZ seizure test, bilateral forelimb tonic extension is usually quickly followed by hindlimb tonic extension and death due to respiratory arrest. Although compound **26** did not significantly affect the threshold for forelimb tonus, it diminished the occurrence of hindlimb tonus in 10 out of 12 mice, whereas compound **28** completely inhibited both fore- and hindlimb tonus in 9 out of 12 mice and hindlimb tonus in 3 mice. This suggests that compound **28** may suppress the spread of seizure activity through the brain, which needs to be further investigated. In mice with no forelimb tonus, PTZ infusion was stopped after 180 s and the threshold dose of PTZ (in mg/kg) was calculated in those mice taking 180 s as infusion duration.

Based on the obtained results, we can conclude that compounds **26** and **28** were more efficient in the *iv*PTZ test than their chemical precursors, i.e., **KJ-5** and **KA-11**. In the present study, both compounds (at a dose of 50 mg/kg) either reduced (compound **26**) or almost completely inhibited (compound **28**) the occurrence of tonic seizures, while **KJ-5** did not affect the threshold for forelimb tonus^18^ and **KA-11** raised the tonic seizure threshold, but only when administered at a twice as high dose, i.e., 100 mg/kg.^15^

### 2.5 Acute Effect on the Neuromuscular Strength in Mice

The grip strength test was performed just before the *iv*PTZ test to assess the acute effect of compounds **26** and **28** on neuromuscular strength. Neither compound **26** nor **28** (50 mg/kg) significantly affect the neuromuscular strength in mice (Figure S1).

### 2.6 Effect on the PTZ-Induced Kindling in Mice

**Figure 3:**
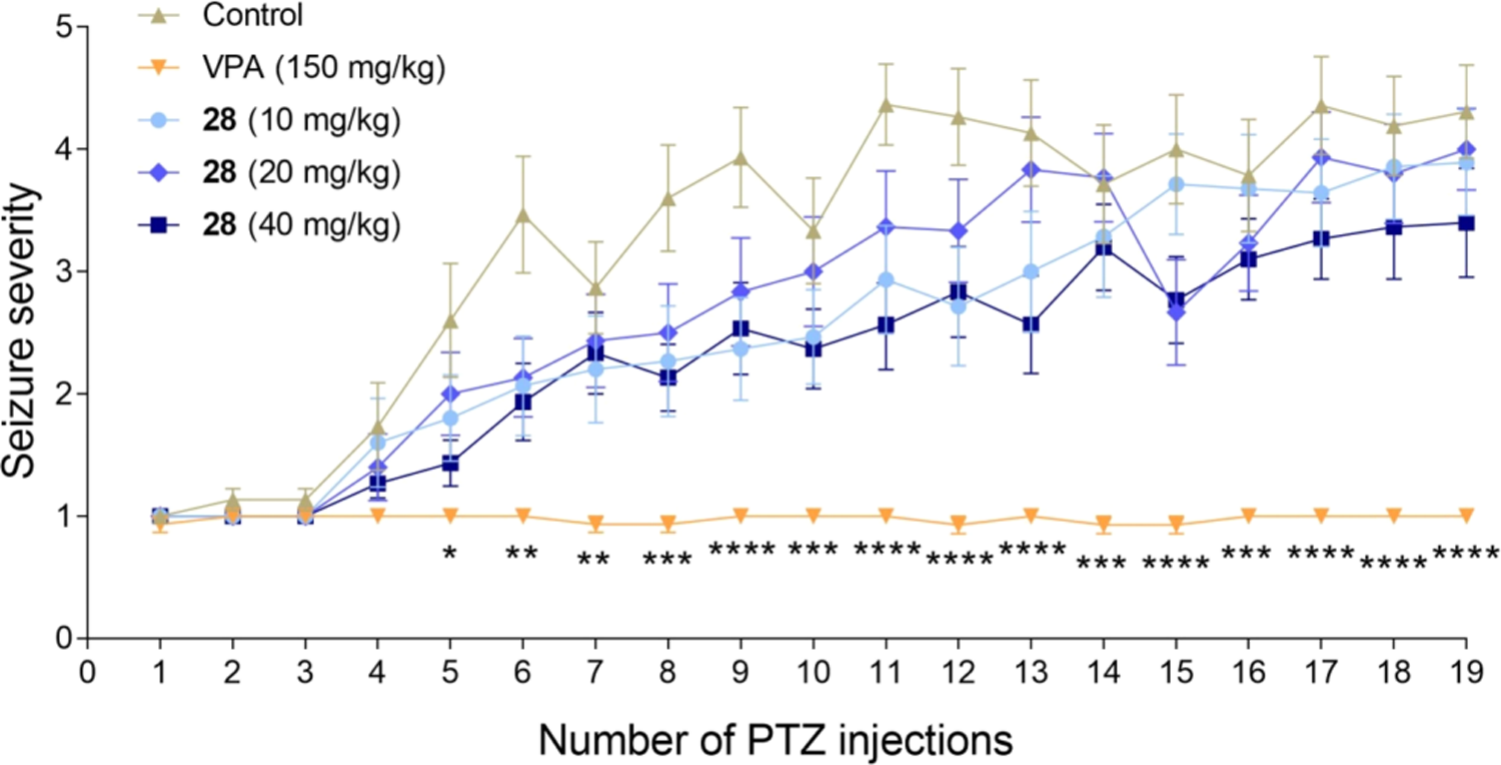
Effect of compound **28** on the progression of PTZ-induced kindling in mice. Compound **28**, VPA, or vehicle were administered *i.p*. every 24 h. PTZ (40 mg/kg, *i.p.*) was given three times a week, 30 min after administration of compound **28**, VPA, or vehicle. Data are shown as means of seizure severity ± SEM (*n* = 13–15 animals). The statistical significance was evaluated by a mixed-effect model for repeated measures followed by Tukey’s post hoc test: *p < 0.05, **p < 0.01, ***p < 0.001, and ****p < 0.0001 as compared to the control group (GraphPad Prism 8).

In the next step, we examined the effect of prolonged treatment with compound **28** on the progression of the PTZ-induced kindling in mice (Figure 3). Repeated injection of PTZ at a subconvulsive dose of 40 mg/kg three times per week gradually increased the mean seizure severity score in the control group from 1.00 ± 0.00 to 4.31 ± 0.38 (after the first and the last PTZ injection, respectively). The percentage of fully kindled mice in the control group was 77%. VPA (positive control) administered at a dose of 150 mg/kg completely suppressed kindling development (0% of fully kindled mice). In contrast, compound **28** administered at doses of 10 and 20 mg/kg did not significantly affect kindling progression and the percentage of fully kindled mice (71 and 73%, respectively). The mean seizure severity score in the group of mice treated with compound **28** at the highest dose (40 mg/kg) was 1.00 ± 0.00 after the first PTZ injection and 3.40 ± 0.45 after the last PTZ injection, while the percentage of fully kindled mice in this group was 47%. These slight differences were not statistically significant as compared to the PTZ control group.

Statistically significant reduction in the development of the PTZ-induced kindling was also noted for **KA-11** (chemical precursor of **28**) at a dose range from 25 to 100 mg/kg.^15^ Thus, weak protection in PTZ-induced kindling by the lead compound (**28**) is consistent with the very weak activity of these chemical derivatives in the *sc*PTZ model and probably too low doses used for the experiment (the best activity in the *sc*PTZ model for the compound with 2-acetamidopropanamide was observed with **24**, and it showed effective protection at doses higher than 100 mg/kg (ED_50_ = 115.6 [100.6–132.8] mg/kg, mice, *i.p.*).

24 h after the last PTZ injection, animals were subjected to behavioral tests to evaluate the effect of compound **28** on spontaneous locomotor activity and anxiety- and depressive-like behavior in PTZ-kindled mice. As compared to the nonkindled control group, there were no changes in locomotor activity, as well as anxiety- and depressive-like behavior in the PTZ-kindled control group. Likewise, repeated administration of VPA and compound **28** did not affect locomotor activity and anxiety-related behavior. In the forced swim test (FST), compound **28** at 10 and 20 mg/kg only slightly decreased (∼10%) the total immobility duration (*p* < 0.05 vs PTZ-kindled control group; Figure S2).

### 2.7 Effect on Glutamate and GABA Concentrations in the Hippocampus and Cortex of PTZ-Kindled Mice

**Figure 4:**
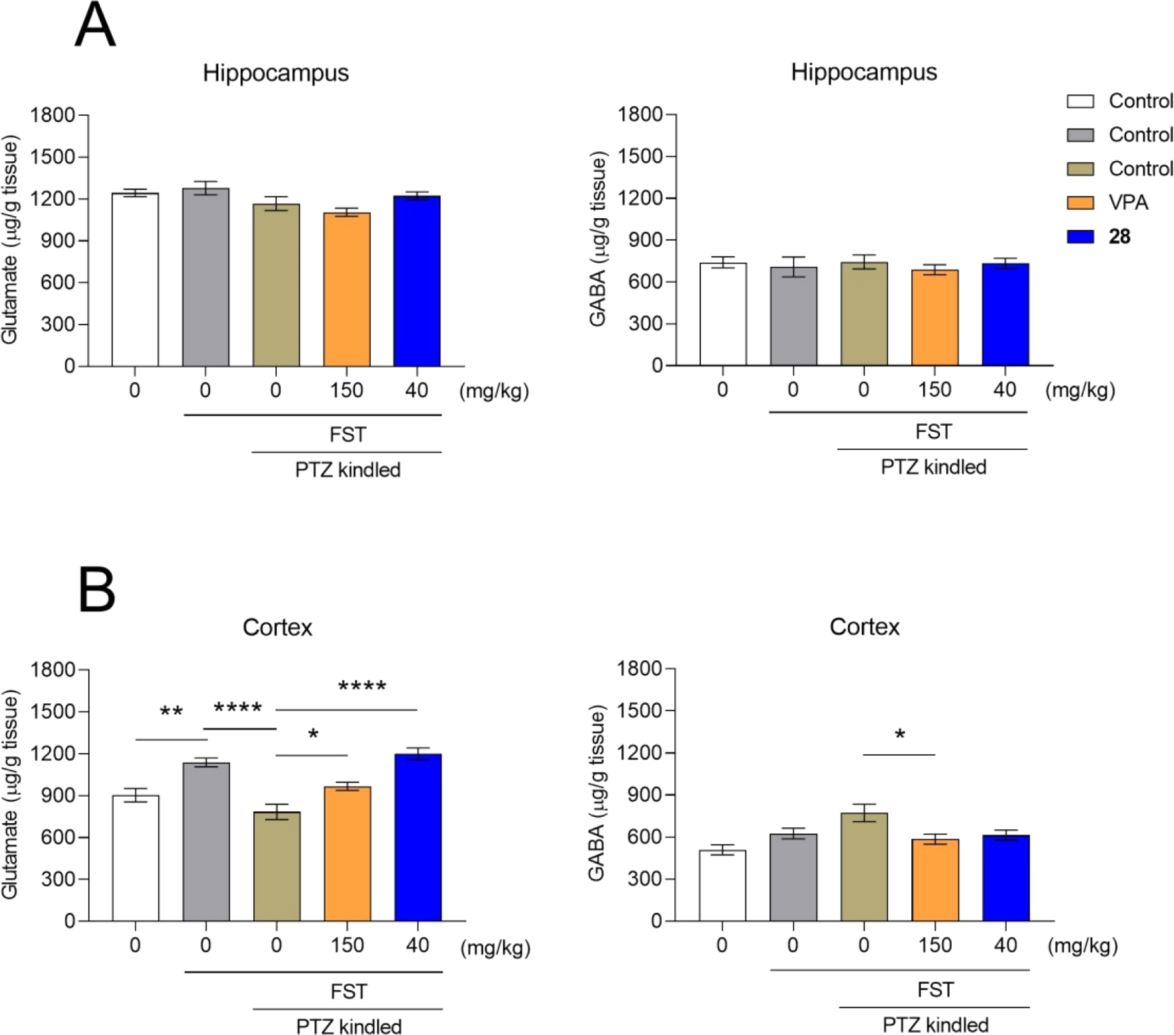
Effect of treatments on glutamate and GABA concentrations in the hippocampus (A) and cortex (B). Data are shown as means of seizure severity ± SEM (*n* = 9–15 animals). The statistical significance was evaluated by one-way analysis of variance (ANOVA) followed by Tukey’s post hoc test: *p < 0.05, **p < 0.01, and ****p < 0.0001 (GraphPad Prism 8).

After completion of behavioral tests, animals were sacrificed and the brains were collected for the analysis of glutamate and GABA concentrations in the hippocampus and cortex. No changes in glutamate and GABA concentrations were reported in the hippocampus (Figure 4A). However, statistically significant differences in the concentration of both glutamate and GABA (Figure 4B) were found in the cortex. The FST procedure increased cortical glutamate concentrations in this brain structure as compared to naive mice (*p* < 0.05), whereas PTZ-induced kindling caused a ∼30% decrease in glutamate concentrations in comparison to the nonkindled control group exposed to the FST test (*p* < 0.0001). Importantly, VPA and compound **28** (40 mg/kg) reversed this effect (*p* < 0.05 and *p* < 0.0001 vs the PTZ-kindled control group, respectively). VPA also decreased GABA concentrations as compared to the PTZ-kindled control animals (*p* < 0.05), while compound **28** did not affect GABA concentrations in the cortex of PTZ-kindled animals.

A decrease in cortical glutamate concentration following the kindling procedure may seem surprising as seizures are generally thought to be associated with increased glutamatergic neurotransmission. It should be however noted that samples were taken 24 h after the last PTZ injection, and concentrations of glutamate and GABA were determined in tissue homogenates, not in dialysates, which would be more adequate. Nevertheless, several animal studies showed that seizures could upregulate glutamate transporters’ expression and thereby in turn may affect glutamate levels. For example, Doi et al.^36^ found increased protein expression of hippocampal EAAT1 and EAAT2 within 24 h after the last seizure in the PTZ-induced kindling in rats. Increased expression of EAAT2 in the hippocampus was also observed 24 h after kainic acid-induced status epilepticus in mice.^37^ It is noteworthy that, similarly to our findings, increased glutamate concentrations were found in homogenates of the hippocampus, striatum, and prefrontal cortex of PTZ-kindled rats. In the same study, no changes in GABA concentrations were reported.^38^ As compound **28** restored the glutamate concentration in the cortex of kindled mice to the control level, it seems that this compound may, at least in part, affect the glutamatergic system. This possibility warrants further investigation.

### 2.8 BNDF and proNGF Expressions in the Hippocampus and Cortex of PTZ-Kindled Mice

Some evidence suggests that increased production of the brain-derived neurotrophic factor (BDNF) after brain injury or seizures may be a key mediator of cellular events underlying epileptogenesis. Indeed, overexpression of BDNF is frequently reported after seizure induction. However, there are also opposite findings, showing that BDNF may inhibit the process of epilepsy development.^39^

**Figure 5:**
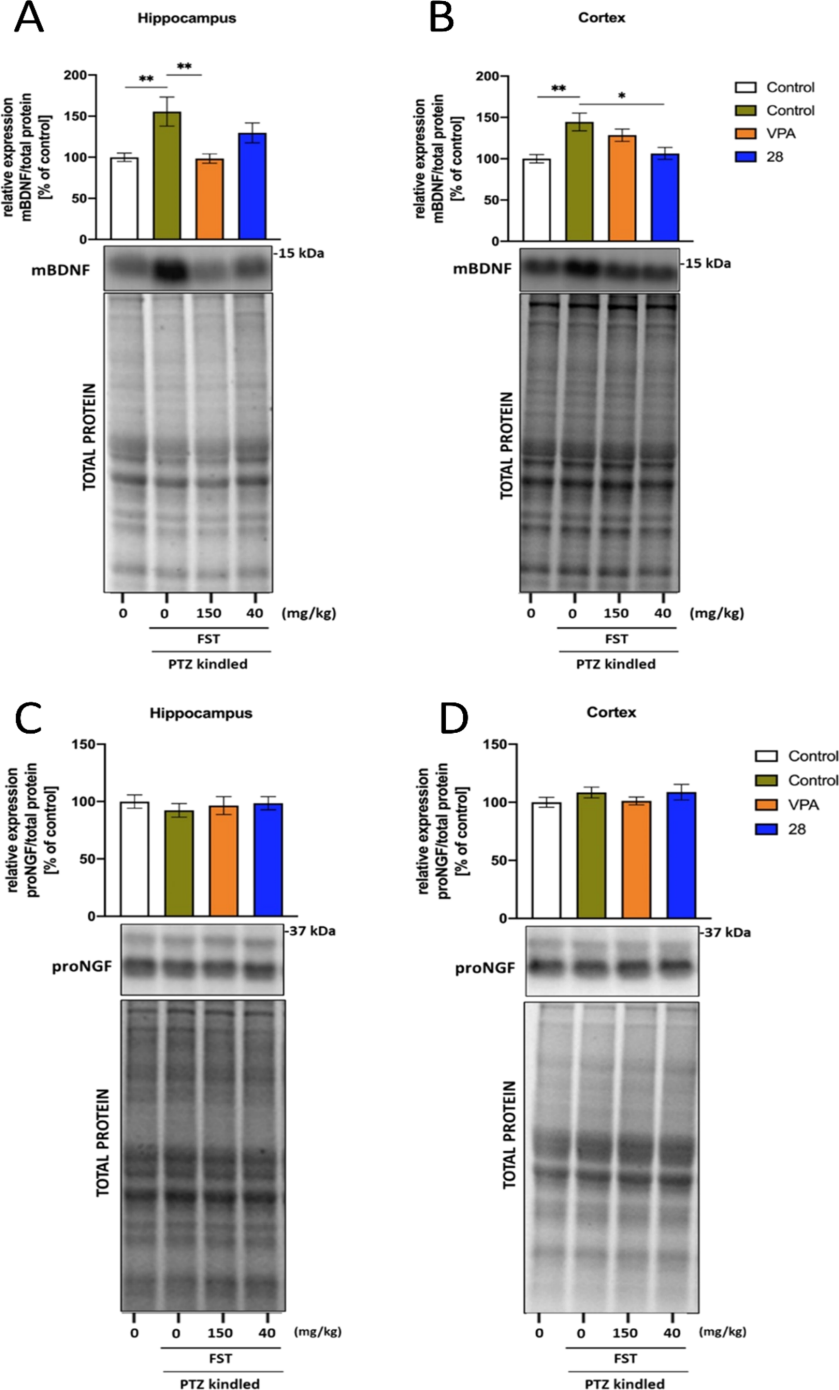
Effect of treatments on mature BDNF (mBDNF) expression in the hippocampus (A) and cortex (B) and on proNGF expression in the hippocampus (C) and cortex (D) with representative immunoblots, together with the total protein amount visualized by the stain-free technique. Data are shown as means of relative expressions ± SEM (*n* = 8 animals). The statistical significance was evaluated by one-way ANOVA followed by Tukey’s post hoc test: *p < 0.05 and **p < 0.01 (GraphPad Prism 8).

In the present study, we found an overexpression of the mature BDNF (mBDNF) protein in the hippocampus and cortex of PTZ-kindled control animals (*p* < 0.01; Figure 5A,B), indicating that BDNF may facilitate seizure progression in this experimental model. Prolonged treatment with compound **28** decreased the expression of mBDNF in the cortex but not in the hippocampus of the PTZ-kindled animals. Conversely, VPA restored the expression of mBDNF to a normal level in the hippocampus but not in the cortex. In addition, we determined the expression of the nerve growth factor (NGF), which can also be implicated in kindling progression, by enhancing mossy fiber sprouting.^40^ However, no changes in the NGF protein expression in the hippocampus and cortex of kindled animals were found (Figure 5C,D).

### 2.9 Antinociceptive Activity

ASMs originally designed to treat epilepsy are extensively used to treat a wide range of disorders other than epilepsy, such as pain, migraine, and bipolar disorder. Thus, future ASMs could also have the potential to treat other nonepileptic disorders. Neuropathic pain represents an important clinical problem due to its prevalence, chronic character, and limited therapeutic options. We used different experimental approaches to evaluate the analgesic activity of compound **28** in neurogenic, inflammatory, and neuropathic pain models.

One of the most useful screening methods for testing clinically relevant molecules is the formalin model. The local injection of formalin solution induces two distinct phases of the nociceptive response, which are associated with immediate activation of nociceptors (phase I) and sensitization of spinal reflex circuits (phase II).^41^ It is important to note that during the second phase, there is also an inflammatory response to tissue damage. Moreover, it has been suggested that formalin injection results in pathological changes that resemble those observed in nerve injury and neuropathic pain. Thus, this model has been successfully used to assess the analgesic efficacy of a variety of compounds, including ASMs.^42^

**Figure 6:**
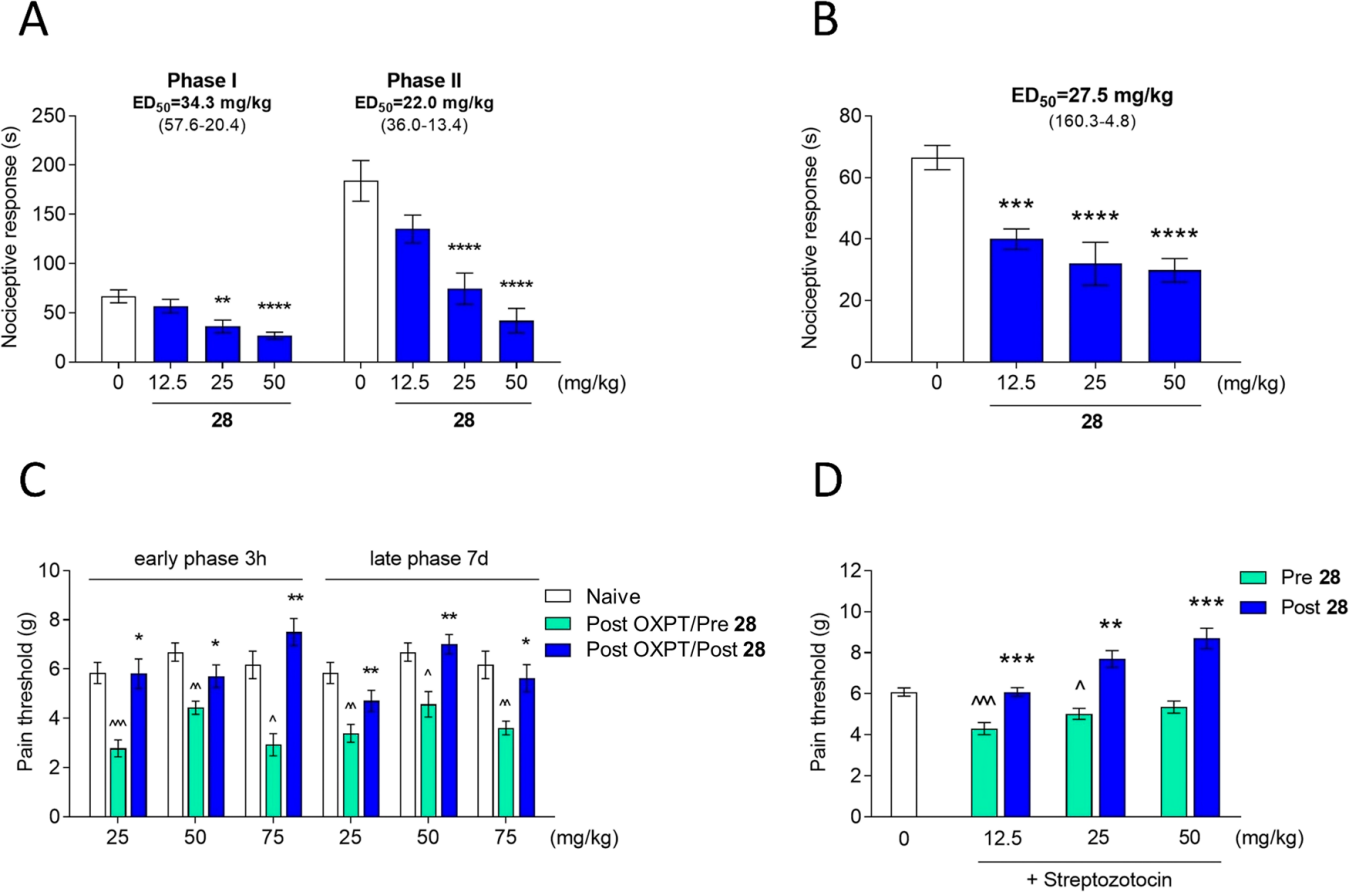
(A) Effect of compound **28** on the duration of licking/biting behavior in the acute phase (0–5 min after formalin injection and in the late phase and 15–30 min after formalin injection). The test compound or vehicle (1% Tween 80) was administered 30 min *i.p.* before the test. (B) The effect of compound **28** on the duration of the nociceptive response in capsaicin-induced pain. The test compound or vehicle (1% Tween 80) was administered 30 min (*i.p.*) before the capsaicin injection. The results are presented as bar plots showing the mean ± SEM. (C) Antiallodynic effects of compound **28** in the tactile allodynia in oxaliplatin (OXPT)-induced peripheral neuropathy. The compound was administered at the doses of 25, 50, and 75 mg/kg 30 min before the evaluation in the von Frey test carried out 3 h and 7 days after OXPT injection. (D) Antiallodynic effects of compound **28** in the tactile allodynia in streptozotocin (STZ)-induced peripheral neuropathy. The compound was administered at the doses of 12.5, 25, and 50 mg/kg 30 min before the evaluation in the von Frey test carried out 21 days after STZ injection. The statistical significance (A, B) was evaluated by one-way ANOVA followed by Dunnett’s post hoc test: **p < 0.01, ***p < 0.001, ****p < 0.0001, *n* = 8–10 mice per group. The statistical significance (C, D) was evaluated by repeated measures analysis of variance (ANOVA), followed by Dunnett’s post hoc comparison: *p < 0.05, **p < 0.01, and ***p < 0.01 when results compared to the OXPT-treated group (Post Oxali/Pre **28**) or STZ-treated group (Post **28**) and ^∧^*p* < 0.05, ^∧∧^*p* < 0.01, and ^∧∧∧^*p* < 0.001 when results compared to naive mice, *n* = 10 mice per group (GraphPad Prism 8).

Administration of compound **28** before the formalin injection significantly attenuated the nociceptive response in mice in both phases of the test. Its ED_50_ value in phase I was found to be 34.3 mg/kg, whereas the ED_50_ value in phase II was found to be 22.0 mg/kg (Figure 6A). Such results revealed the wide spectrum of analgesic properties of the tested compound.

Compound **28** was active in phase I of the formalin test, showing the potential to inhibit the acute pain of neurogenic origin. Since the formalin-induced pain is mainly dependent on the chemical stimulation of the TRPA1 receptor on somatosensory nerve endings, we decided to evaluate the influence of compound **28** on TRPV1-dependent pain using capsaicin, an agonist of those types of receptors.^43^ The compound significantly decreased the paw licking or biting behavior in that test at all administrated doses (12.5, 25, and 50 mg/kg). The ED_50_ value in that test was 27.5 mg/kg, which shows that the potency in inhibiting TRPV1-dependent pain is higher than the potency in attenuating TRPA1-dependent pain (Figure 6B).

In further studies, we used two models of neuropathic pain to evaluate the influence of compound **28** on pain resulting from neuronal tissue damage by oxaliplatin (OXPT), which induces central and peripheral sensitization.^44^ In diabetic neuropathy, impaired peripheral nerve function and neuronal damage result from a variety of vascular and metabolic abnormalities.^45^

We assessed the antiallodynic effect of compound **28** in the OXPT-induced neuropathy using the von Frey method. The method is based on measuring the mean force that caused the paw withdrawal reaction, i.e., pain threshold. In all tested groups, the administration of OXPT resulted in a significant decrease in the pain threshold measured 3 h and 7 days after OXPT injection, which corresponds with the early and late phases of neuropathic pain. In the group treated with **28** at a dose of 25 mg/kg (Figure 6C), the initial value of 5.84 ± 0.43 (baseline) was decreased to the value of 2.78 ± 0.34 (47.6% of the baseline) and 3.39 ± 0.36 (58.0% of the baseline) in the early and late phases, respectively. The single administration of the test compound significantly reversed the effect of OXPT in the early (99.5% of the baseline) and late (58.1% of the baseline) phases. In the group treated with **28** at a dose of 50 mg/kg, the initial value of 6.69 ± 0.37 decreased to the values of 4.43 ± 0.27 (66.2% of the baseline) and 4.57 ± 0.52 (68.3% of the baseline) in the early and the late phases, respectively. The single administration of compound **28** significantly attenuated the effect of OXPT in the early phase (85.4% of the baseline) and completely abolished allodynia in the late phase (104.8% of the baseline). In the group treated with the test compound at a dose of 75 mg/kg, the initial value of 6.17 ± 0.56 decreased to the values of 2.93 ± 0.45 (47.5% of the baseline) and 3.61 ± 0.28 (58.5% of the baseline) in the early and late phases, respectively. The single administration of **28** resulted in a higher pain threshold than the baseline (121.5% of the baseline) in the early phase. In the late phase, allodynia was also significantly reversed (91.3% of the baseline).

We also used the von Frey method to evaluate the influence of compound **28** on mechanical allodynia developed as the effect of streptozotocin-induced hyperglycemia. The single *i.p.* administration of streptozotocin (STZ) at a dose of 200 mg/kg resulted in allodynia observed as the decreased pain threshold. In the first group, the initial value of 6.08 ± 0.29 (baseline) decreased to the value of 4.30 ± 0.21 (70.7% of the baseline). The treatment with **28** at a dose of 12.5 mg/kg completely inhibited allodynia (100.0% of the baseline, Figure 6D). In the second group, the initial value of 6.08 ± 0.29 (baseline) decreased to the value of 5.02 ± 0.27 (82.6% of the baseline). The administration of **28** at a dose of 25 mg/kg elevated the pain threshold to the value of 7.69 ± 0.41, which corresponds with 126.5% of the baseline. In the third group, the baseline was decreased to the value of 5.35 ± 0.27 (88.0% of the baseline). The effect was not statistically significant. The administration of **28** at a dose of 50 mg/kg elevated the pain threshold to a value as high as 8.70 ± 0.41, which corresponds with 143.1% of the baseline.

We aimed to test the influence of the test compound on spontaneous locomotor activity to investigate its sedative properties. The strong sedative activity of new compounds is considered an undesirable property, which may lead to an incorrect or ambiguous interpretation of the *in vivo* results. Moreover, it may prove to be an additional limitation in the case of potential clinical application of new drug candidates. Tested compound **28** decreased spontaneous locomotor activity in mice at all tested doses (Figure S3). However, there was not a significant difference between this effect in mice receiving 50 and 100 mg/kg, which suggests that the sedative property of **28** has a ceiling effect, while the analgesic activity of the compound is dose-dependent.

Summing up the results of analgesic tests, we found out that compound **28** showed very broad spectra of activity attenuating acute, neurogenic, inflammatory, and neuropathic pain, which was similar to previously tested **KA-11** and **KA-104**. All of the above-mentioned compounds decreased the duration of the nociceptive response in the acute and late phases of the formalin test. However, **KA-104** showed the highest potency (a lower amount of a drug that is needed to produce a given effect), which is expressed as an ED_50_ value. The difference can be clearly observed in the acute phase, where the ED_50_ value was 71.7 mg/kg for **KA-11** and 34.3 mg/kg for compound **28**, while the value for **KA-104** was 28.5 mg/kg. The difference in potency was even higher in the late phase, where the ED_50_ value was 29.3 mg/kg for **KA-11**, 22.0 mg/kg for compound **28**, and 12.4 mg/kg for **KA-104**. All three compounds were also tested in the oxaliplatin-induced model of neuropathic pain. In the test, **KA-11** showed very high efficacy. The compound not only reversed the allodynic effect of oxaliplatin but, at a dose of 150 mg/kg, also elevated the pain threshold to 179% of the initial value. The efficacy of **KA-104** and compound **28** was not as high. However, the tested doses were also significantly lower. The highest tested dose for **KA-104** was 30 mg/kg, which elevated the pain threshold to 124% of the initial value.^15,17^ Considering all results, the analgesic activity of the compared structures can be ranked as follows: **KA-104** > compound **28** > **KA-11**.

### 2.10 Pharmacokinetic Studies

The data presented in Figures S4 and S5 and Table 4 indicated that **28** was very rapidly absorbed from the intraperitoneal cavity gastrointestinal tract of mice. The time to reach the maximum concentration (*t*_max_) was 5 min, which is the first sampling point in all cases. The elimination half-lives (*t*_1/2_) were similar in serum and brain after both doses and routes of administration (*i.p.* and *p.o.*), and they were relatively long, ranging from 58.62 to 111.39 min. A more than proportional increase in *C*_max_ and area under the curve (AUC) was observed with increasing *i.p.* doses. The 2-fold greater *i.p.* dose of **28** resulted in 3.41- and 4.11-fold increases in *C*_max_ and AUC, respectively, in serum, indicating the saturation of metabolism or elimination of the compound in this dose range. Consequently, CL/*F* (calculated as dose/AUC) was almost 2 times lower following the higher dose. A similar supraproportional increase in **28** exposure was observed in brain tissue. The volume of distribution (*V*_*z*_/*F*) was larger than mouse body water, and the value was almost 2 times lower for the higher dose. Administration of the studied compound orally led to quite similar values of AUC in both serum and brain in comparison to the *i.p.* administration of the same dose. However, the maximal concentration was about three times lower than after *i.p.* dosing. Compound **28** very well penetrated the blood–brain barrier. The brain-to-serum *C*_max_ and AUC ratio exceeded the value of 1 for both doses and routes of administration.

**Table 4:** Pharmacokinetic Parameters of **28** Estimated in Serum and Brain Following *i.p.* or *p.o.* Administration of This Compound to Mice

	estimate					
	serum (*i.p.*)		brain (*i.p.*)		serum (*p.o.*)	brain (*p.o.*)
pharmacokinetic parameter (unit)^a^	25 mg/kg	50 mg/kg	25 mg/kg	50 mg/kg	25 mg/kg	
*t*_max_ (min)	5	5	5	5	5	5
*C*_max_ (ng/mL (g))	11,033.3	37,566.7	13,683.3	39,800.0	3740.0	3799.4
λ_*z*_ [1/min]	0.007	0.008	0.006	0.007	0.012	0.007
*t*_0.5λ*z*_ [min]	94.29	85.42	111.39	106.52	58.62	97.20
AUC_0–*t*_ [ng·min/mL (g)]	254,384.6	1,045,787.2	347,914.8	1,284,194.3	297,899.7	333,360.7
AUC_0–∞_ [ng·min/mL (g)]	254,465.4	1,045,940.8	348,212.5	1,285,007.9	298,019.5	334,667.7
*V_z_*/*F* [L/kg]	13.36	5.89			7.10	
CL/*F* [L/min/kg]	0.098	0.048			0.084	
MRT [min]	22.01	24.40	23.65	26.43	100.68	110.45

a Pharmacokinetic parameters: *t*_max_, time to reach *C*_max_; *C*_max_, maximum serum/brain concentration; λ_*z*_, terminal slope; *t*_1/2λ*z*_, terminal half-life; AUC_0–∞_, area under the curve; *V*_*z*_/*F*, volume of distribution; CL/*F*, clearance; MRT, mean residence time.

### 2.11 *In Vitro* Radioligand Binding Studies and Functional Assays

Due to the structural similarities of the most potent compounds (**26** and **28**) to the previously reported chemical prototypes (**KA-11** and **KJ-5**), it is postulated that they may possess a similar multimodal mechanism of action, which include, among others, antagonism of sodium channels, Ca_v_1.2 calcium channels, and blockade of the transient receptor potential cation channel vanilloid type 1 (TRPV1).^15,16,18^ Importantly, sodium or calcium conductance is often associated with the pathophysiology of epilepsy as well as neuropathic pain.^46−50^ Therefore, both sodium and calcium ion channels are well-established molecular targets for structurally diverse ASMs such as LCS, lamotrigine, carbamazepine, oxcarbazepine, etc.^51^ Consequently, we carried out binding and/or functional studies toward the sodium channel (site 2) and calcium Ca_v_1.2 channel at high concentrations (100 μM) of **26** and **28** (Table 5). Surprisingly, none of the tested compounds (**26** and **28**), despite the strong structural similarities to **KA-11** and **KJ-5**, showed a significant effect on both sodium and Ca_v_1.2 channels. Next, **26** and **28** due to the presence of the phenylpiperazine moiety, which is a well-recognized pharmacophore for serotoninergic receptor (5-HT_*x*_R) ligands,^52^ were evaluated for their influence on 5-HT_2C_R, where stimulation plays a key role for the antiseizure activity of lorcaserin (one of the newest ASMs).^6^ Unfortunately, both **26** and **28** did not possess any 5-HT2CR agonist effect as tested at a concentration of 10 μM.

**Table 5:** *In Vitro* Binding and Functional Assays for **26** and **28**

		% inhibition of control specific binding (concentration [μM])^a^	
binding studies	source	**26**	**28**
Na^+^ channel (site 2)	rat cerebral cortex	4.5 (100)	–6.9 (100)

		% inhibition of control agonist response (concentration [μM])^a^	
functional studies	source	**26**	**28**
Ca_v_1.2 (h) calcium ion channel cell-based antagonist calcium flux assay	human recombinant HEK-293 cell	7.0 (100)	–7.9 (100)
5-HT_2C_ (h) (agonist effect)	human recombinant HEK-293 cell	0.5 (10)	0.1 (10)

a Results showing activity higher than 50% are considered to represent significant effects of the test compounds, results showing an inhibition between 25 and 50% are indicative of moderate effect, and results showing an inhibition lower than 25% are not considered significant and mostly attributable to the variability of the signal around the control level.

For many years, the TRPV1 channel, permeable to calcium and sodium ions, has been recognized as a promising molecular target for novel analgesics.^19^ More importantly, most recent neurobiological studies showed that TRPV1 channels are also located in the CNS (i.e., hippocampus, cortex), and their excessive activation may play an important role in the induction of seizures and thus may be involved in the epileptogenesis process.^53,54^ Notably, this hypothesis has been confirmed in our previous research.^16,18,55^ Therefore, bearing in mind the involvement of TRPV1 in pain sensation and potentially in seizure induction, in the next step of the *in vitro* studies, the TRPV1 antagonist activity was determined for all compounds obtained (Table 6).

**Table 6:** *In Vitro* TRPV1 Channel Antagonist Activity for Compounds **21**–**36** and **45**–**48** (Concentration of 10 μM)

TRPV1 (VR1) (h) (antagonist effect)^a^			
cmpd	% inhibition of control agonist response^b^	cmpd	% inhibition of control agonist response^b^
**21**	1.6	**31**	4.6
**22**	12.5	**32**	–16.1
**23**	–4.6	**33**	–11.7
**24**	51.5	**34**	–5.3
**25**	8.1	**35**	–11.9
**26**	–4.9	**36**	–13.6
**27**	–15.9	**45**	–11.6
**28**	6.5	**46**	–10.0
**29**	104.2	**47**	–8.3
**30**	12.0	**48**	4.2

a Source: human recombinant Chinese hamster ovary (CHO) cells.

b Results showing activity higher than 50% are considered to represent significant effects of the test compounds, results showing an inhibition between 25 and 50% are indicative of a weak effect, and results showing an inhibition lower than 25% are not considered significant and mostly attributable to the variability of the signal around the control level. Assays were performed commercially in Eurofins Laboratories (Poitiers, France).

Surprisingly, only two molecules, namely, **24** and **29**, showed activity higher than 50% (at 10 μM) and were characterized by IC_50_ = 35 μM and *K*_B_ = 4.5 μM for **24** and IC_50_ = 32 μM and *K*_B_ = 4.2 μM for **29**. Although both **24** and **29** revealed potent antiseizure activity, they were not the most effective compounds identified during *in vivo* characterization in seizure models. As a result, it is hard to clearly show the correlation between TRPV1 antagonist activity and antiseizure efficacy. Furthermore, structurally related phenylglycine derivative **KJ-5** showed distinctly stronger TRPV1 antagonist properties but, in contrast, revealed weaker antiseizure activity in all tests applied (compared to its direct alanine analogue—**26**).^18^ Unfortunately, none of the applied chemical modifications in Series 2 and Series 3, aiming to improve the TRPV1 antagonist properties, caused the desired effect on this molecular target.

In order to confirm or exclude additional targets, which may be responsible for antiseizure (or antinociceptive) activity, lead compound **28** was tested for interaction with a broader range of other receptors, ion channels, or the GABA transporter, which are known to be involved in the mechanism of action of several and clinically used ASMs (Table 7). Moreover, we also evaluated its influence on the potassium channel (hERG), which is known to be one of the most critical “off-targets” responsible for the harmful proarrhythmic activity of drugs and drug candidates.^56^ As a result, **28** did not interact with NMDA and AMPA receptors, Ca_v_2.2 calcium channels, GABA_A_ receptor, GABA_A_ transporter, GABA transaminase, and notably hERG channel at a concentration of 100 μM. The latter result is particularly important, since it minimizes the risk of potential proarrhythmic activity of **28**. Despite this positive result, more detailed off-target profiling is undoubtedly necessary in the next steps of preclinical development.

**Table 7:** Additional *In Vitro* Binding Assays for **28** (Concentration of 100 μM)

binding studies	source	% inhibition of control specific binding (concentration [μM])^a^
NMDA (antagonist radioligand)	rat cerebral cortex	5.0
AMPA (agonist radioligand)	rat cerebral cortex	–21.0
Ca_v_2.2 calcium ion channel (antagonist radioligand)	rat cerebral cortex	3.6
GABA_A_ transporter (antagonist radioligand)	rat cerebral cortex	15.0
GABA_A_ ion channel [^3^H]GABA (agonist radioligand)	rat hippocampus	10.0
GABA transaminase	rat brain	–1.9
potassium channel (hERG)	human recombinant HEK-293 cell	12.9

a Results showing activity higher than 50% are considered to represent significant effects of the test compounds, results showing an inhibition between 25 and 50% are indicative of a weak effect, and results showing an inhibition lower than 25% are not considered significant and mostly attributable to the variability of the signal around the control level. Binding studies were performed commercially in Eurofins Laboratories (Poitiers, France). All assays were performed in duplicate.

Due to the relatively high concentration of compounds used in the *in vitro* assays, as well as brain concentration of **28** (>10 μM after 25 mg/kg dose administration) determined in the pharmacokinetic studies (see Section 2.10), it seems unlikely that compounds described herein act by any of mechanisms tested in the aforementioned binding and functional studies. The potent protection of **28** in electrically induced seizure, which is characteristic of sodium channel blockers, may suggest its influence on sodium conductance. Thus, in further *in vitro* assays, **28** was also evaluated for its influence on fast voltage-gated sodium channels in rat prefrontal cortex pyramidal neurons using the patch-clamp technique (maximal currents were evoked by rectangular voltage steps to −10 mV).

**Figure 7:**
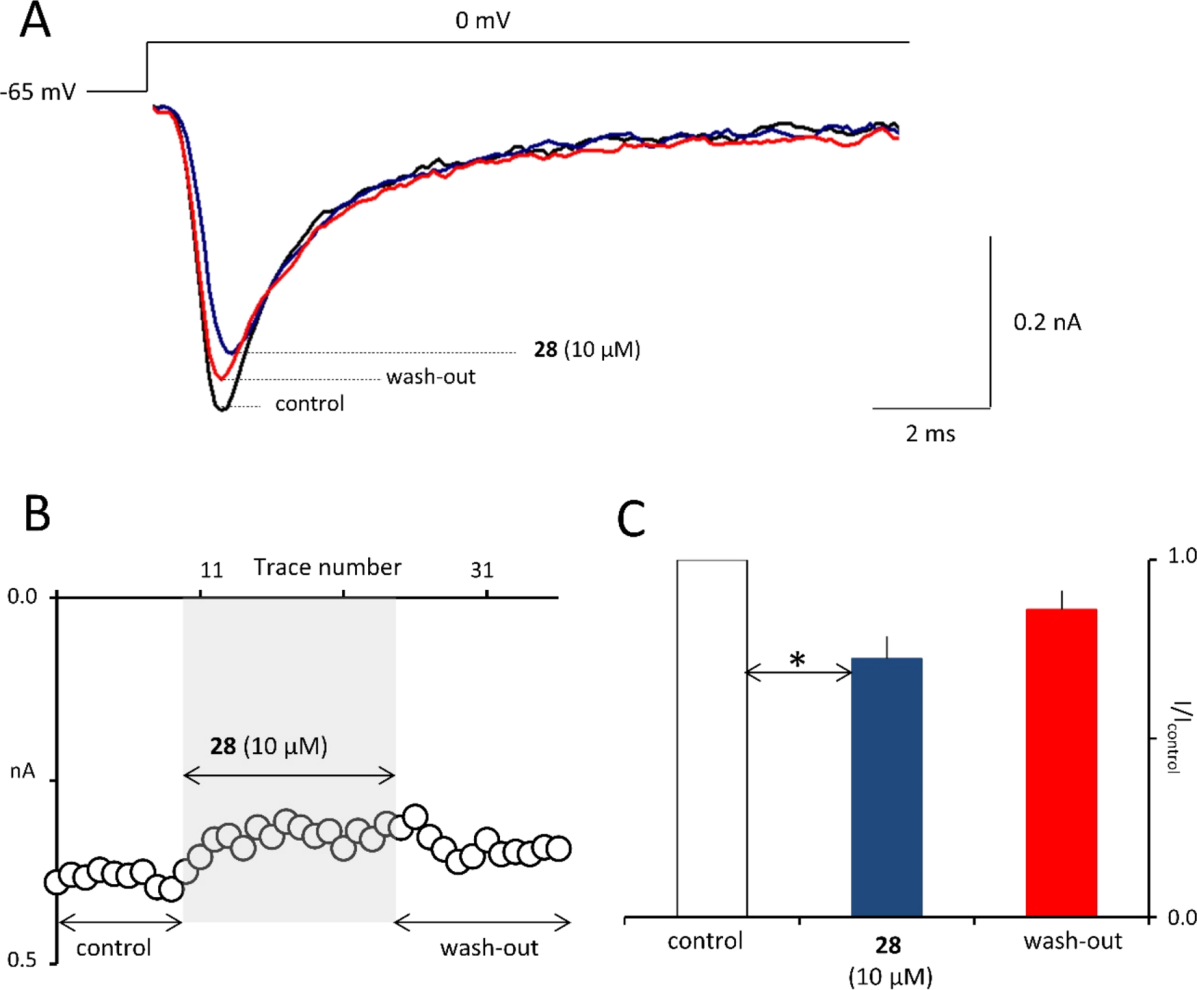
Compound **28** inhibits fast voltage-gated sodium currents in prefrontal cortex pyramidal neurons. (A) Sodium current recordings in control (black trace), after application of **28** (blue trace), and after wash-out (red trace). Current traces were evoked by a rectangular voltage step. (B) Influence of **28** on sodium current is shown on an example neuron. Current traces were evoked once every 10 s. The vertical axis shows maximal current amplitudes (white circles) in control, in the presence of **28**, and after wash-out. The horizontal axis shows the trace number. (C) Averaged normalized maximal sodium current amplitudes in control, in the presence of **28**, and after wash-out. The statistical significance was evaluated by one-way ANOVA with Tukey’s post hoc test (*n* = 5): *p < 0.05 (GraphPad Prism 8).

Consequently, as shown in Figure 7, **28** at a concentration of 10 μM decreased significantly voltage-gated sodium currents, and this effect was partially reversible after wash-out. The averaged, maximal normalized amplitudes of voltage-gated sodium currents were 1.0 in control, 0.72 ± 0.06 after application of **28**, and 0.86 ± 0.05 after wash-out (control vs **28***p* < 0.05). These results suggest that the antiseizure activity of **28** and structurally related compounds may be mediated (among others) by inhibition of CNS sodium conductance, leading to the decrease of neuronal firing.

### 2.12 *In Vitro* ADME-Tox Assays

Several *in vitro* assays were applied in order to evaluate the most crucial ADME-Tox parameters of compounds **26** and **28**. The obtained results were compared to the selected reference drugs and are included in Table 8.

**Table 8:** ADME-Tox Parameters Determined *In Vitro* for **26** and **28**

ADME-Tox assay *in vitro*	**26**	**28**	reference drug
PAMPA—*P*_e_ (10^–6^ cm/s) ± SD	4.51 ± 0.61	5.95 ± 0.51	9.78 ± 1.75 (caffeine)
plasma protein binding—fraction bound *f*_b_ (% ±SD)	78.6 ± 7.72	83 ± 1.73	98.3 ± 7.72 (warfarin)
plasma protein binding—*K*_D_ (μM)	1670.0	1260.0	104.0 (warfarin)
metabolic stability (% remaining after the reaction with HLMs)	93.5	90.6	30.8 (verapamil)
CYP3A4 activity (% of control ± SD at 1 μM)	114.1 ± 8.7	91.3 ± 12.7	5.05 ± 0.16 (ketoconazole)
CYP2D6 activity (% of control ± SD at 1 μM)	122.8 ± 11.7	115.0 ± 4.5	4.38 ± 0.66 (quinidine)
HepG2 viability (% of control ± SD)	103.8 ± 8.8 at 1.56 μM	88.2 ± 16.1 at 1.56 μM	10.8 ± 0.5 at 1.74 μM (doxorubicin)

The precoated parallel artificial membrane permeability assay (PAMPA) was used to determine the passive mechanism of permeability. Both tested compounds showed a higher permeability coefficient (*P*_e_) than 1.5 × 10^–6^ cm/s—recommended by the PAMPA Plate System manufacturer value for permeable compounds. However, the *P*_e_ values of **26** and **28** were lower than that estimated for the reference high-permeable caffeine (9.78 × 10^–6^ cm/s). Furthermore, **26** showed slightly worse ability to cross the artificial membrane in comparison to **28** (4.51 vs 5.95 × 10^–6^ cm/s; Table 8).

The plasma protein binding: human serum albumin (HSA) and α_1_-acid glycoprotein (AGP) was evaluated by the commercial TRANSIL^XL^ PPB assay. Both compounds showed similar binding to the mixture of HSA and AGP, much lower than the affinity of the highly bound reference warfarin (*f*_b_ 98.3%, *K*_D_ 104 μM).

Both tested compounds were found metabolically stable in comparison to the reference drug verapamil after incubation with human liver microsomes (HLMs). The remaining fractions of **26** and **28** after reaction with HLMs were similar (93.5 and 90.6%, respectively) and were much higher than the remaining fraction of verapamil (30.8%) incubated with microsomes under the same conditions (see details in Table 8 and Figures S8 and S9).^57−59^ Moreover, two main metabolites of **26** were found, whereas three metabolites were observed for **28**. The most probable metabolic pathways were identified in MS spectra supported by in silico data (MetaSite 6.0.1 software) as dehydrogenation, oxidation, or hydroxylation (Table S3 and Figures S6, S7, S10, and S11).

**Figure 8:**
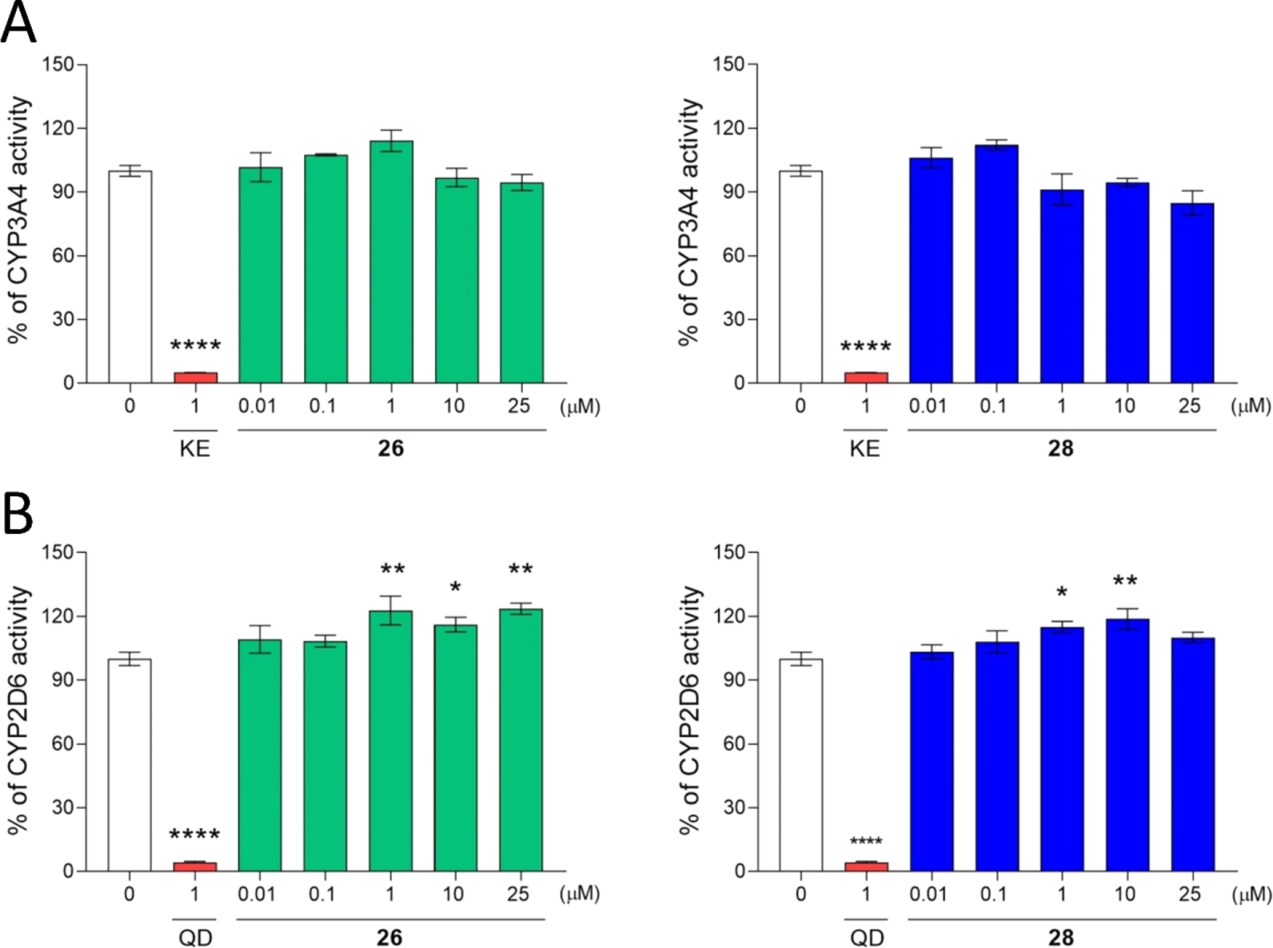
Effect of **26**, **28**, and the reference drugs ketoconazole (KE) and quinidine (QD) on CYP3A4 (A) and CYP2D6 (B) activity. Statistical significance (*p < 0.05, **p < 0.01, ****p < 0.0001) was analyzed by GraphPad Prism 8.0.1 software using one-way ANOVA and Bonferroni’s multiple comparison post test.

The interaction with the two most common drug metabolism CYP (cytochrome P450) isoforms, CYP3A4 and CYP2D6, were investigated to assess the drug–drug interaction (DDI) potential of **26** and **28**. The results were compared to 1 μM of the reference CYP inhibitors: ketoconazole (KE, CYP3A4) and quinidine (QD, CYP2D6). Both tested compounds did not show any influence on CYP3A4 activity (Table 8 and Figure 8A). A modest but statistically significant induction of CYP2D6 was observed (Table 8 and Figure 8B). This effect was consistent with our previous studies using pyrrolidine-2,5-dione compounds and their acetyl analogues.^16−18,55^

**Figure 9:**
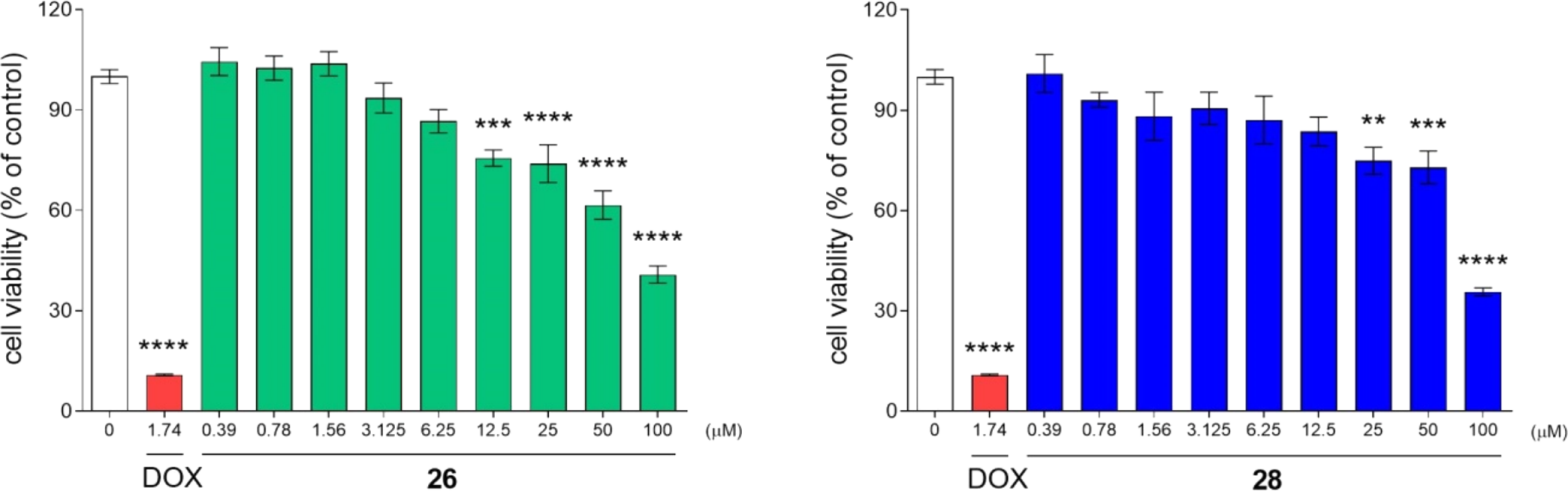
Effect of **26**, **28**, and the reference drug doxorubicin (DOX) on the HepG2 cell viability. Statistical significance (**p < 0.01, ***p < 0.001, ****p < 0.0001) was analyzed by GraphPad Prism 8.0.1 software using one-way ANOVA and Bonferroni’s multiple comparison post test.

The *in vitro* safety tests were performed with the use of a hepatoma HepG2 cell line to estimate the hepatotoxic potential of the tested compounds. A statistically significant, although rather modest, decrease of cell viability was observed from 12.5 μM (**26**) or 25 μM (**28**). However, approximately 40% cell survival was noted after exposure to a high concentration (100 μM) of **26** and **28**. The hepatotoxicity of both tested compounds is predicated as modest in comparison to the activity of the reference cytotoxic compound, doxorubicin (DOX) (only 10.8% of HepG2 viability at 1.74 μM; Table 8 and Figure 9).

## 3 Conclusions

In the present study, based on the combinatorial chemistry approach, we obtained a series of 20 chemically original compounds, and several of them showed robust antiseizure and antinociceptive properties. They were effective *in vivo* in the standard seizure models, i.e., the maximal electroshock (MES) test, the psychomotor 6 Hz (32 mA) seizure model, and, importantly, the 6 Hz (44 mA) model of DRE. The most active compounds, **26** and **28**, were also effective in the *iv*PTZ seizure threshold test and did not affect the neuromuscular strength. Moreover, lead compound **28** was tested in the PTZ-induced kindling model of epileptogenesis, and its effect on glutamate and GABA levels in the hippocampus and cortex was also measured. In addition, **28** revealed potent efficacy in formalin-induced tonic pain, capsaicin-induced pain, and OXPT-induced peripheral neuropathy, as well as STZ-induced peripheral neuropathy. Finally, pharmacokinetic and *in vitro* studies indicated acceptable drug-like ADME-Tox properties, making it an interesting candidate for further preclinical development. Compounds described herein are likely sodium channel blockers as tested for **28** applying the patch-clamp technique; nevertheless, more elaborate electrophysiological studies including Na_v_*x* subtypes could be the next step in the mechanism of action investigations.

Since the compounds reported in the current studies are racemates, further pharmacological characterization and development should include asymmetric synthesis yielding specific enantiomers that will allow better structure–activity relationship evaluation.

In summary, the obtained results indicate that **28** may be a promising candidate for further development with potential therapeutic utility in epilepsy and neuropathic pain.

## 4 Methods

### 4.1 Chemistry

All chemicals and solvents were purchased from commercial suppliers and were used without further purification. Melting points (mp) were determined in open capillaries on a Büchi 353 melting point apparatus (Büchi Labortechnik, Flawil, Switzerland). Thin-layer chromatography (TLC) and gradient UPLC chromatography were used to assess the purity and homogeneity of the compounds. TLC was carried out on silica gel 60 F_254_-precoated aluminum sheets (Macherey-Nagel, Düren, Germany), using the following developing systems: S_1_—DCM/MeOH (9:0.3; v/v) and S_2_—DCM/MeOH (9:0.5; v/v). Spots detection: UV light (λ = 254 nm). The UPLC and mass spectra (LC-MS) were obtained on a Waters ACQUITY TQD system (Waters, Milford, CT) with an MS-TQ detector and a UV–vis-DAD eλ detector. The ACQUITY UPLC BEH C18, 1.7 μm (2.1 × 100 mm^2^) column was used with the VanGuard Acquity UPLC BEH C18, 1.7 μm (2.1 × 5 mm^2^, Waters, Milford, CT). Standard solutions (1 mg/mL) of each compound were prepared in an analytical grade MeCN/water mixture (1:1; v/v). Conditions applied were as follows: eluent A (water/0.1% HCOOH), eluent B (MeCN/0.1% HCOOH), a flow rate of 0.3 mL/min, a gradient of 5–100% B over 10 min, and an injection volume of 10 μL. The UPLC analyses and high-resolution mass spectra (LC-HRMS) were obtained on a Waters ACQUITY I-Class PLUS SYNAPT XS High-Resolution Mass Spectrometer (Waters, Milford, CT) with an MS-Q-TOF detector and a UV–vis-DAD eλ detector. The ACQUITY UPLC BEH C18, 1.7 μm (2.1 × 100 mm^2^) column was used with the VanGuard Acquity UPLC BEH C18, 1.7 μm (2.1 × 5 mm^2^) (Waters, Milford, CT). Standard solutions (1 mg/mL) of each compound were prepared in analytical grade MeCN/water mixture (1:1; v/v). Conditions applied were as follows: eluent A (water/0.1% HCOOH), eluent B (MeCN/0.1% HCOOH), a flow rate of 0.3 mL/min, a gradient of 5–100% B over 13 min, and an injection volume of 10 μL. The UPLC retention times (*t*_R_) were given in minutes. The purity of target compounds determined by the use of the chromatographic UPLC method was ≥97.5%. Preparative column chromatography was performed using silica gel 60 (particle size 0.063–0.200; 70–230 mesh ATM) purchased from Merck (Darmstadt, Germany). ^1^H NMR and ^13^C NMR spectra were obtained in a JEOL-500 spectrometer (JEOL USA, Inc. MA) in CDCl_3_ operating at 500 MHz (^1^H NMR) and 126 MHz (^13^C NMR). Chemical shifts are reported in δ values (ppm) relative to tetramethylsilane (TMS) δ = 0 (^1^H) as an internal standard (IS). The *J* values are expressed in Hertz (Hz). Signal multiplicities are represented by the following abbreviations: s (singlet), br s (broad singlet), d (doublet), dd (double doublet), t (triplet), td (triple doublet), q (quartet), and m (multiplet).

#### General Method for the Preparation of Compounds **1**–**10**

Carbonyldiimidazole (0.51 g, 3.1 mmol, 1.2 equiv) was dissolved in 10 mL of dichloromethane (DCM). Afterward, this solution was added to Boc-dl-alanine (0.5 g, 2.6 mmol, 1 equiv) and dissolved in 10 mL of DCM (while stirring). After 0.5 h, the respective commercial or noncommercial (**A5**–**A8**) piperazine derivatives (2.6 mmol, 1 equiv) dissolved in 5 mL of DCM were added dropwise. The mixture was stirred for approximately 3 h at room temperature and evaporated to dryness. Column chromatography was applied for purification of crude products using developing system S_1_. The desired compounds were obtained as light oils, followed by the concentration of organic solvents under reduced pressure.

#### *tert*-Butyl (1-oxo-1-(4-Phenylpiperazin-1-yl)propan-2-yl)carbamate (**1**)

Light yellow oil. Yield 88% (0.78 g); TLC: *R*_f_ = 0.81 (S_1_); UPLC (purity >99%): *t*_R_ = 6.29 min. LC-MS (ESI): *m*/*z* calcd for C_18_H_27_N_3_O_3_ (M + H)^+^ 334.21, found 334.4.

#### *tert*-Butyl (1-(4-(3-Chlorophenyl)piperazin-1-yl)-1-oxopropan-2-yl)carbamate (**2**)

Light yellow oil. Yield 85% (0.82 g); TLC: *R*_f_ = 0.85 (S_1_); UPLC (purity >99%): *t*_R_ = 7.17 min. LC-MS (ESI): *m*/*z* calcd for C_18_H_26_N_3_O_3_Cl (M + H)^+^ 368.17, found 368.2.

#### *tert*-Butyl (1-(4-(4-Chlorophenyl)piperazin-1-yl)-1-oxopropan-2-yl)carbamate (**3**)

Light yellow oil. Yield 82% (0.79 g); TLC: *R*_f_ = 0.83 (S_1_); UPLC (purity >99%): *t*_R_ = 7.20 min. LC-MS (ESI): *m*/*z* calcd for C_18_H_26_N_3_O_3_Cl (M + H)^+^ 368.17, found 368.4.

#### *tert*-Butyl (1-(4-(3,4-Dichlorophenyl)piperazin-1-yl)-1-oxopropan-2-yl)carbamate (**4**)

Light yellow oil. Yield 86% (0.91 g); TLC: *R*_f_ = 0.88 (S_1_); UPLC (purity >99%): *t*_R_ = 7.74 min. LC-MS (ESI): *m*/*z* calcd for C_18_H_25_N_3_O_3_Cl_2_ (M + H)^+^ 403.13, found 403.1.

#### *tert*-Butyl (1-(4-(3,5-Dichlorophenyl)piperazin-1-yl)-1-oxopropan-2-yl)carbamate (**5**)

Light yellow oil. Yield 89% (0.95 g); TLC: *R*_f_ = 0.86 (S_1_); UPLC (purity = 98.5%): *t*_R_ = 8.09 min. LC-MS (ESI): *m*/*z* calcd for C_18_H_25_N_3_O_3_Cl_2_ (M + H)^+^ 403.13, found 403.3.

#### *tert*-Butyl (1-oxo-1-(4-(3-(Trifluoromethyl)phenyl)piperazin-1-yl)propan-2-yl)carbamate (**6**)

Light yellow oil. Yield 90% (0.95 g); TLC: *R*_f_ = 0.88 (S_1_); UPLC (purity >99%): *t*_R_ = 7.41 min. LC-MS (ESI): *m*/*z* calcd for C_19_H_26_N_3_O_3_F_3_ (M + H)^+^ 402.20, found 402.1.

#### *tert*-Butyl (1-oxo-1-(4-(4-(Trifluoromethyl)phenyl)piperazin-1-yl)propan-2-yl)carbamate (**7**)

Light yellow oil. Yield 87% (0.92 g); TLC: *R*_f_ = 0.86 (S_1_); UPLC (purity >99%): *t*_R_ = 7.46 min. LC-MS (ESI): *m*/*z* calcd for C_19_H_26_N_3_O_3_F_3_ (M + H)^+^ 402.20, found 402.3.

#### *tert*-Butyl (1-oxo-1-(4-(3-(Trifluoromethoxy)phenyl)piperazin-1-yl)propan-2-yl) carbamate (**8**)

Light yellow oil. Yield 88% (0.97 g); TLC: *R*_f_ = 0.89 (S_1_); UPLC (purity >99%): *t*_R_ = 7.69 min. LC-MS (ESI): *m*/*z* calcd for C_19_H_26_N_3_O_4_F_3_ (M + H)^+^ 418.19, found 418.3.

#### *tert*-Butyl (1-oxo-1-(4-(3-Phenoxyphenyl)piperazin-1-yl)propan-2-yl)carbamate (**9**)

Light yellow oil. Yield 87% (0.97 g); TLC: *R*_f_ = 0.90 (S_1_); UPLC (purity = 98.2%): *t*_R_ = 7.88 min. LC-MS (ESI): *m*/*z* calcd for C_24_H_31_N_3_O_4_ (M + H)^+^ 426.23, found 426.2.

#### *tert*-Butyl (1-oxo-1-(4-(3-((Trifluoromethyl)thio)phenyl)piperazin-1-yl)propan-2-yl)carbamate (**10**)

Yellow oil. Yield 86% (0.98 g); TLC: *R*_f_ = 0.87 (S_1_); UPLC (purity >99%): *t*_R_ = 8.02 min. LC-MS (ESI): *m*/*z* calcd for C_19_H_26_N_3_O_3_SF_3_ (M + H)^+^ 434.17, found 434.3.

#### General Method for the Preparation of Compounds **11**–**20**

The solution of **1**–**10** (2.2 mmol, 1 equiv) in DCM (10 mL) was treated with TFA (3 equiv) and stirred at room temperature for 3 h. Afterward, the organic solvents were evaporated to dryness. The resulting oil residue was dissolved in water (20 mL), and then 25% ammonium hydroxide was carefully added to pH = 8. The aqueous layer was extracted with DCM (3 × 20 mL), dried over Na_2_SO_4_, and concentrated to give **11**–**20** as yellow oils. Intermediates **11**–**20** were advanced as substrates without purification for the last reaction.

#### 2-Amino-1-(4-phenylpiperazin-1-yl)propan-1-one (**11**)

Yellow oil. Yield 98% (0.50 g); TLC: *R*_f_ = 0.20 (S_2_); UPLC (purity >99%): *t*_R_ = 2.62 min. LC-MS (ESI): *m*/*z* calcd for C_13_H_19_N_3_O (M + H)^+^ 234.16, found 234.2.

#### 2-Amino-1-(4-(3-chlorophenyl)piperazin-1-yl)propan-1-one (**12**)

Yellow oil. Yield 96% (0.57 g); TLC: *R*_f_ = 0.22 (S_2_); UPLC (purity >99%): *t*_R_ = 3.67 min. LC-MS (ESI): *m*/*z* calcd for C_13_H_18_N_3_OCl (M + H)^+^ 268.12, found 268.2.

#### 2-Amino-1-(4-(4-chlorophenyl)piperazin-1-yl)propan-1-one (**13**)

Yellow oil. Yield 97% (0.57 g); TLC: *R*_f_ = 0.21 (S_2_); UPLC (purity >99%): *t*_R_ = 4.01 min. LC-MS (ESI): *m*/*z* calcd for C_13_H_18_N_3_OCl (M + H)^+^ 268.12, found 268.4.

#### 2-Amino-1-(4-(3,4-dichlorophenyl)piperazin-1-yl)propan-1-one (**14**)

Yellow oil. Yield 97% (0.64 g); TLC: *R*_f_ = 0.24 (S_2_); UPLC (purity >99%): *t*_R_ = 4.26 min. LC-MS (ESI): *m*/*z* calcd for C_13_H_17_N_3_OCl_2_ (M + H)^+^ 303.07, found 303.3.

#### 2-Amino-1-(4-(3,5-dichlorophenyl)piperazin-1-yl)propan-1-one (**15**)

Yellow oil. Yield 95% (0.63 g); TLC: *R*_f_ = 0.23 (S_2_); UPLC (purity >99%): *t*_R_ = 4.84 min. LC-MS (ESI): *m*/*z* calcd for C_13_H_17_N_3_OCl_2_ (M + H)^+^ 303.07, found 303.3.

#### 2-Amino-1-(4-(3-(trifluoromethyl)phenyl)piperazin-1-yl)propan-1-one (**16**)

Yellow oil. Yield 98% (0.65 g); TLC: *R*_f_ = 0.24 (S_2_); UPLC (purity >99%): *t*_R_ = 4.05 min. LC-MS (ESI): *m*/*z* calcd for C_14_H_18_N_3_OF_3_ (M + H)^+^ 302.14, found 302.0.

#### 2-Amino-1-(4-(4-(trifluoromethyl)phenyl)piperazin-1-yl)propan-1-one (**17**)

Yellow oil. Yield 96% (0.64 g); TLC: *R*_f_ = 0.25 (S_2_); UPLC (purity >99%): *t*_R_ = 4.49 min. LC-MS (ESI): *m*/*z* calcd for C_14_H_18_N_3_OF_3_ (M + H)^+^ 302.14, found 302.2.

#### 2-Amino-1-(4-(3-(trifluoromethoxy)phenyl)piperazin-1-yl)propan-1-one (**18**)

Yellow oil. Yield 97% (0.68 g); TLC: *R*_f_ = 0.28 (S_2_); UPLC (purity >99%): *t*_R_ = 4.59 min. LC-MS (ESI): *m*/*z* calcd for C_14_H_18_N_3_O_2_F_3_ (M + H)^+^ 318.13, found 318.4.

#### 2-Amino-1-(4-(3-phenoxyphenyl)piperazin-1-yl)propan-1-one (**19**)

Yellow oil, yield 96% (0.69 g); TLC: *R*_f_ = 0.27 (S_2_); UPLC (purity >99%): *t*_R_ = 4.89 min. LC-MS (ESI): *m*/*z* calcd for C_19_H_23_N_3_O_2_ (M + H)^+^ 326.18, found 326.3.

#### 2-Amino-1-(4-(3-((trifluoromethyl)thio)phenyl)piperazin-1-yl)propan-1-one (**20**)

Yellow oil, yield 97% (0.71 g); TLC: *R*_f_ = 0.27 (S_2_); UPLC (purity >99%): *t*_R_ = 4.87 min. LC-MS (ESI): *m*/*z* calcd for C_14_H_18_N_3_OSF_3_ (M + H)^+^ 334.12, found 334.3.

#### General Method for the Preparation of Final Compounds **21**–**30**

Intermediates **11**–**20** (2 mmol, 1 equiv) were dissolved in 10 mL of DCM. Afterward, triethylamine (TEA) (6 mmol, 3 equiv) was added while stirring at 0 °C to the solution. Final compounds **21**–**30** were prepared by dropwise adding acetyl chloride (3 mmol, 1.5 equiv) at 0 °C on an ice bath. The reaction mixture was allowed to warm up to room temperature and was stirred for an additional 2 h and evaporated to dryness. Next, the crude product was purified by applying column chromatography using developing system S_2_. The desired compounds were obtained as white solids, followed by the concentration of organic solvents under reduced pressure and wash-up by diethyl ether.

#### *N*-(1-oxo-1-(4-Phenylpiperazin-1-yl)propan-2-yl)acetamide (**21**)

White solid. Yield: 92% (0.51 g); mp 113.6–114.9 °C; TLC: *R*_f_ = 0.40 (S_2_); UPLC (purity >99%): *t*_R_ = 3.90 min. LC-MS (ESI): *m*/*z* calcd for C_15_H_21_N_3_O_2_ (M + H)^+^ 276.17 found 276.3. ^1^H NMR (500 MHz, CDCl_3_) δ 1.33 (d, *J* = 6.9 Hz, 3H, CH_3_), 1.99 (s, 3H, CH_3_), 3.05–3.25 (m, 4H, piperazine), 3.56–3.65 (m, 1H, piperazine), 3.67–3.76 (m, 2H, piperazine), 3.78–3.91 (m, 1H, piperazine), 4.92 (quin, *J* = 7.0 Hz, 1H, CH), 6.70 (br d, *J* = 6.9 Hz, 1H, NH) 6.85–6.99 (m, 3H, ArH) 7.27 (t, *J* = 7.7 Hz, 2H, ArH). ^13^C NMR (126 MHz, CDCl_3_) δ 19.3, 23.4, 42.1, 45.1, 45.5, 49.5, 49.8, 116.9, 120.9, 129.4, 150.8, 169.4, 171.0.

#### *N*-(1-(4-(3-Chlorophenyl)piperazin-1-yl)-1-oxopropan-2-yl)acetamide (**22**)

White solid. Yield: 95% (0.59 g); mp 125.8–126.5 °C; TLC: *R*_f_ = 0.42 (S_2_); UPLC (purity >99%): *t*_R_ = 5.08 min. LC-MS (ESI): *m*/*z* calcd for C_15_H_20_N_3_O_2_Cl (M + H)^+^ 310.13 found 310.3. UPLC/HRMS (purity >99%): *t*_R_ = 5.48 min. HRMS (ESI-QTOF): *m*/*z* calcd for 310.1278, found 310.1310. ^1^H NMR (500 MHz, CDCl_3_) δ 1.32 (d, *J* = 6.9 Hz, 3H, CH_3_), 1.99 (s, 3H, CH_3_), 3.06–3.24 (m, 4H, piperazine), 3.55–3.65 (m, 1H, piperazine), 3.66–3.74 (m, 2H, piperazine), 3.77–3.88 (m, 1H, piperazine), 4.92 (quin, *J* = 6.9 Hz, 1H, CH), 6.66 (br d, *J* = 6.9 Hz, 1H, NH), 6.74–6.80 (m, 1H, ArH), 6.83–6.89 (m, 2H, ArH), 7.16 (t, *J* = 8.0 Hz, 1H, ArH). ^13^C NMR (126 MHz, CDCl_3_) δ 19.2, 23.4, 41.9, 45.1, 45.2, 49.0, 49.3, 114.6, 116.6, 120.5, 130.3, 135.2, 151.8, 169.4, 171.1.

#### *N*-(1-(4-(4-Chlorophenyl)piperazin-1-yl)-1-oxopropan-2-yl)acetamide (**23**)

White solid. Yield: 90% (0.56 g); mp 144.9–145.8 °C; TLC: *R*_f_ = 0.44 (S_2_); UPLC (purity >99%): *t*_R_ = 5.17 min. LC-MS (ESI): *m*/*z* calcd for C_15_H_20_N_3_O_2_Cl (M + H)^+^ 310.13 found 310.5. ^1^H NMR (500 MHz, CDCl_3_) δ 1.32 (d, *J* = 6.9 Hz, 3H, CH_3_), 1.99 (s, 3H, CH_3_), 2.96–3.24 (m, 4H, piperazine), 3.57–3.65 (m, 1H, piperazine), 3.66–3.73 (m, 2H, piperazine), 3.83 (dd, *J* = 6.3, 3.4 Hz, 1H, piperazine), 4.91 (quin, *J* = 7.02 Hz, 1H, CH), 6.64 (br d, *J* = 7.5 Hz, 1H, NH), 6.78–6.87 (m, 2H, ArH), 7.17–7.23 (m, 2H, ArH). ^13^C NMR (126 MHz, CDCl_3_) δ 19.3, 23.4, 42.0, 45.1, 45.3, 49.4, 49.8, 118.1, 125.8, 129.2, 149.4, 169.3, 171.0.

#### *N*-(1-(4-(3,4-Dichlorophenyl)piperazin-1-yl)-1-oxopropan-2-yl)acetamide (**24**)

White solid. Yield: 92% (0.63 g); mp 152.1–152.9 °C; TLC: *R*_f_ = 0.46 (S_2_); UPLC (purity >99%): *t*_R_ = 5.74 min. LC-MS (ESI): *m*/*z* calcd for C_15_H_19_N_3_O_2_Cl_2_ (M + H)^+^ 344.09 found 344.1. UPLC/HRMS (purity >99%): *t*_R_ = 6.09 min. HRMS (ESI-QTOF): *m*/*z* calcd for 344.0888, found 344.0915. ^1^H NMR (500 MHz, CDCl_3_) δ 1.32 (d, *J* = 6.9 Hz, 3H, CH_3_), 1.98 (s, 3H, CH_3_), 3.06–3.22 (m, 4H, piperazine), 3.56–3.64 (m, 1H, piperazine), 3.64–3.74 (m, 2H, piperazine), 3.82 (dd, *J* = 6.3, 3.4 Hz, 1H, piperazine), 4.91 (quin, *J* = 7.0 Hz, 1H, CH), 6.63 (br d, *J* = 7.5 Hz, 1H, NH), 6.69–6.76 (m, 1H, ArH), 6.94 (d, *J* = 2.3 Hz, 1H, ArH), 7.27 (d, *J* = 8.6 Hz, 1H, ArH). ^13^C NMR (126 MHz, CDCl_3_) δ 19.2, 23.4, 41.8, 45.1, 45.1, 48.9, 49.3, 116.1, 118.1, 123.5, 130.7, 133.1, 150.2, 169.4, 171.1.

#### *N*-(1-(4-(3,5-Dichlorophenyl)piperazin-1-yl)-1-oxopropan-2-yl)acetamide (**25**)

White solid. Yield: 91% (0.62 g); mp 164.6–165.4 °C; TLC: *R*_f_ = 0.48 (S_2_); UPLC (purity = 98.5%): *t*_R_ = 6.16 min. LC-MS (ESI): *m*/*z* calcd for C_15_H_19_N_3_O_2_Cl_2_ (M + H)^+^ 344.09 found 344.5. UPLC/HRMS (purity = 98.4%): *t*_R_ = 6.36 min. HRMS (ESI-QTOF): *m*/*z* calcd for 344.0888, found 344.0915. ^1^H NMR (500 MHz, CDCl_3_) δ 1.32 (d, *J* = 6.9 Hz, 3H, CH_3_), 1.99 (s, 3H, CH_3_), 3.10–3.26 (m, 4H, piperazine), 3.56–3.64 (m, 1H, piperazine), 3.64–3.74 (m, 2H, piperazine), 3.82 (dd, *J* = 6.4, 3.4 Hz, 1H, piperazine), 4.91 (quin, *J* = 7.0 Hz, 1H, CH), 6.59 (br d, *J* = 7.5 Hz, 1H, NH), 6.73 (d, *J* = 1.72 Hz, 2H, ArH), 6.84 (t, *J* = 1.7 Hz, 1H, ArH). ^13^C NMR (126 MHz, CDCl_3_) δ 19.2, 23.4, 41.8, 45.0, 45.1, 48.5, 48.8, 114.6, 120.1, 135.7, 152.1, 169.4, 171.1.

#### *N*-(1-oxo-1-(4-(3-(Trifluoromethyl)phenyl)piperazin-1-yl)propan-2-yl)acetamide (**26**)

White solid. Yield: 93% (0.64 g); mp 127.9–128.7 °C; TLC: *R*_f_ = 0.50 (S_2_); UPLC (purity >99%): *t*_R_ = 5.49 min. LC-MS (ESI): *m*/*z* calcd for C_16_H_20_N_3_O_2_F_3_ (M + H)^+^ 344.15 found 344.2. UPLC/HRMS (purity >99%): *t*_R_ = 5.86 min. HRMS (ESI-QTOF): *m*/*z* calcd for 344.1541, found 344.1565. ^1^H NMR (500 MHz, CDCl_3_) δ 1.33 (d, *J* = 6.9 Hz, 3H, CH_3_), 1.99 (s, 3H, CH_3_), 3.05–3.33 (m, 4H, piperazine), 3.61–3.68 (m, 1H, piperazine), 3.68–3.78 (m, 2H, piperazine), 3.80–3.89 (m, 1H, piperazine), 4.93 (quin, *J* = 7.0 Hz, 1H, CH), 6.64 (br d, *J* = 7.5 Hz, 1H, NH), 7.05 (br d, *J* = 8.0 Hz, 1H, ArH), 7.08–7.15 (m, 2H, ArH), 7.36 (t, *J* = 8.0 Hz, 1H, ArH). ^13^C NMR (126 MHz, CDCl_3_) δ 19.3, 23.4, 41.9, 45.1, 45.3, 49.0, 49.3, 113.0 (br d, *J* = 3.6 Hz), 117.1 (br d, *J* = 3.6 Hz), 119.6, 124.2 (q, *J* = 272.0 Hz) 129.9, 131.7 (q, *J* = 31.39 Hz), 151.0, 169.4, 171.1.

#### *N*-(1-oxo-1-(4-(4-(Trifluoromethyl)phenyl)piperazin-1-yl)propan-2-yl)acetamide (**27**)

White solid. Yield: 90% (0.62 g); mp 132.8–133.3 °C; TLC: *R*_f_ = 0.51 (S_2_); UPLC (purity >99%): *t*_R_ = 5.67 min. LC-MS (ESI): *m*/*z* calcd for C_16_H_20_N_3_O_2_F_3_ (M + H)^+^ 344.15 found 344.4. ^1^H NMR (500 MHz, CDCl_3_) δ 1.34 (d, *J* = 6.9 Hz, 3H, CH_3_), 2.00 (s, 3H, CH_3_), 3.17–3.36 (m, 4H, piperazine), 3.60–3.67 (m, 1H, piperazine), 3.68–3.77 (m, 2H, piperazine), 3.86 (dd, *J* = 6.6, 3.7 Hz, 1H, piperazine), 4.93 (quin, *J* = 7.0 Hz, 1H, CH), 6.58 (br d, *J* = 6.9 Hz, 1H, NH), 6.92 (d, *J* = 8.6 Hz, 2H, ArH), 7.50 (d, *J* = 8.6 Hz, 2H, ArH). ^13^C NMR (126 MHz, CDCl_3_) δ 19.3, 23.4, 41.8, 45.1, 48.2, 48.5, 115.3, 121.78 (q, *J* = 32.6 Hz), 124.6 (q, *J* = 270.4 Hz), 126.7 (d, *J* = 3.6 Hz), 152.8, 169.4, 171.1.

#### *N*-(1-oxo-1-(4-(3-(Trifluoromethoxy)phenyl)piperazin-1-yl)propan-2-yl)acetamide (**28**)

White solid. Yield: 94% (0.67 g); mp 113.6–114.4 °C; TLC: *R*_f_ = 0.54 (S_2_); UPLC (purity = 97.6%): *t*_R_ = 5.85 min. LC-MS (ESI): *m*/*z* calcd for C_16_H_20_N_3_O_3_F_3_ (M + H)^+^ 360.15 found 360.3. UPLC/HRMS (purity = 97.7%): *t*_R_ = 6.08 min. HRMS (ESI-QTOF): *m*/*z* calcd for 360.1490, found 360.1589. ^1^H NMR (500 MHz, CDCl_3_) δ 1.33 (d, *J* = 6.9 Hz, 3H, CH_3_), 1.99 (s, 3H, CH_3_), 3.11–3.25 (m, 4H, piperazine), 3.58–3.66 (m, 1H, piperazine), 3.67–3.75 (m, 2H, piperazine), 3.84 (dd, *J* = 6.4, 3.4 Hz, 1H, piperazine), 4.92 (quin, *J* = 7.0 Hz, 1H, CH) 6.62 (br d, *J* = 7.5 Hz, 1H, NH), 6.70 (s, 1H, ArH), 6.73 (br d, *J* = 8.0 Hz, 1H, ArH), 6.80 (dd, *J* = 8.6, 2.3 Hz, 1H, ArH), 7.24–7.29 (m, 1H, ArH). ^13^C NMR (126 MHz, CDCl_3_) δ 19.3, 23.4, 41.9, 45.1, 45.2, 48.8, 49.2, 109.1, 112.4, 114.5, 120.5 (q, *J* = 257.1 Hz), 130.3, 150.4, 152.1, 169.4, 171.1.

#### *N*-(1-oxo-1-(4-(3-Phenoxyphenyl)piperazin-1-yl)propan-2-yl)acetamide (**29**)

White solid. Yield: 91% (0.67 g); mp 133.6–134.3 °C; TLC: *R*_f_**=** 0.55 (S_2_); UPLC (purity >99%): *t*_R_ = 6.02 min. LC-MS (ESI): *m*/*z* calcd for C_21_H_25_N_3_O_3_ (M + H)^+^ 368.19 found 368.3. UPLC/HRMS (purity >99%): *t*_R_ = 6.39 min. HRMS (ESI/TOF): *m*/*z* calcd for 368.1929, found 368.1994. ^1^H NMR (500 MHz, CDCl_3_) δ 1.32 (d, *J* = 6.9 Hz, 3H, CH_3_), 1.99 (s, 3H, CH_3_), 3.06–3.23 (m, 4H, piperazine), 3.55–3.65 (m, 1H, piperazine), 3.65–3.74 (m, 2H, piperazine), 3.77–3.87 (m, 1H, piperazine), 4.91 (quin, *J* = 7.0 Hz, 1H, CH), 6.51 (dd, *J* = 8.0, 2.3 Hz, 1H, NH), 6.58 (t, *J* = 2.0 Hz, 1H, ArH), 6.62–6.69 (m, 2H, ArH), 6.96–7.02 (m, 2H, ArH), 7.09 (t, *J* = 7.5 Hz, 1H, ArH), 7.20 (t, *J* = 8.0 Hz, 1H, ArH), 7.28–7.36 (m, 2H, ArH). ^13^C NMR (126 MHz, CDCl_3_) δ 19.3, 23.4, 42.0, 45.1, 45.3, 49.1, 49.5, 107.4, 110.9, 111.4, 119.0, 123.4, 129.8, 130.3, 152.2, 157.2, 158.4, 169.3, 171.0.

#### *N*-(1-oxo-1-(4-(3-((Trifluoromethyl)thio)phenyl)piperazin-1-yl)propan-2-yl)acetamide (**30**)

White solid. Yield: 93% (0.7 g); mp 110.9–111.7 °C; TLC: *R*_f_**=** 0.57 (S_2_); UPLC (purity = 98.6%): *t*_R_ = 6.11 min. LC-MS (ESI): *m*/*z* calcd for C_16_H_20_N_3_O_2_SF_3_ (M + H)^+^ 376.13 found 376.3. UPLC/HRMS (purity = 98.7%): *t*_R_ = 6.45 min. HRMS (ESI/TOF): *m*/*z* calcd for 376.1262, found 376.1325. ^1^H NMR (500 MHz, CDCl_3_) δ 1.33 (d, *J* = 6.9 Hz, 3H, CH_3_), 1.99 (s, 3H, CH_3_), 3.12–3.27 (m, 4H, piperazine), 3.60–3.68 (m, 1H, piperazine), 3.68–3.76 (m, 2H, piperazine), 3.84 (dd, *J* = 6.4, 3.7 Hz, 1H, piperazine), 4.92 (quin, *J* = 7.0 Hz, 1H, CH), 6.63 (br d, *J* = 7.5 Hz, 1H, NH), 7.00 (dd, *J* = 8.31, 2.43 Hz, 1H, ArH), 7.11–7.18 (m, 2H, ArH), 7.27–7.33 (m, 1H, ArH).^13^C NMR (126 MHz, CDCl_3_) δ 19.3, 23.4, 41.9, 45.1, 45.2, 48.9, 49.3, 118.8, 123.9, 125.4, 129.7 (q, *J* = 308.0 Hz), 128.0, 130.2, 151.4, 169.3, 171.1.

#### General Method for the Preparation of Compounds **31** and **32**

Carbonyldiimidazole (0.39 g, 2.4 mmol, 1.2 equiv) was dissolved in 10 mL of tetrahydrofuran (THF). Afterward, this solution was added to benzoic acid (0.24 g, 2 mmol, 1 equiv) and dissolved in 10 mL of THF (while stirring). After 0.5 h, the appropriate amine derivatives (**12** or **16**, 2 mmol, 1 equiv) dissolved in 5 mL of DCM were added dropwise. The mixture was stirred for approximately 12 h at room temperature and evaporated to dryness. Column chromatography was applied for the purification of crude products using developing system S_2_. The desired compound was obtained as light oil, followed by the concentration of organic solvents under reduced pressure.

#### *N*-(1-(4-(3-Chlorophenyl)piperazin-1-yl)-1-oxopropan-2-yl)benzamide (**31**)

White solid. Yield: 88% (0.65 g); mp 177.8–178.5 °C; TLC: *R*_f_ = 0.55 (S_2_); UPLC (purity >99%): *t*_R_ = 6.71 min. LC-MS (ESI): *m*/*z* calcd for C_20_H_22_N_3_O_2_Cl (M + H)^+^ 372.14, found 372.5. ^1^H NMR (500 MHz, CDCl_3_) δ 1.45 (d, *J* = 6.9 Hz, 3H, CH_3_), 3.12–3.27 (m, 4H, piperazine), 3.60–3.81 (m, 3H, piperazine), 3.86 (dd, *J* = 6.4, 3.4 Hz, 1H, piperazine), 5.12 (quin, *J* = 7.0 Hz, 1H, CH), 6.74–6.81 (m, 1H, ArH), 6.84–6.90 (m, 2H, ArH), 7.18 (t, *J* = 8.0 Hz, 1H, ArH), 7.34 (br d, *J* = 7.45 Hz, 1H, NH), 7.39–7.46 (m, 2H, ArH), 7.46–7.53 (m, 1H, ArH), 7.77–7.86 (m, 2H, ArH). ^13^C NMR (126 MHz, CDCl_3_) δ 19.3, 42.0, 45.3, 45.6, 49.0, 49.3, 114.7, 116.6, 120.5, 127.1, 128.6, 130.3, 131.8, 134.1, 135.2, 151.8, 166.5, 171.1.

#### *N*-(1-oxo-1-(4-(3-(Trifluoromethyl)phenyl)piperazin-1-yl)propan-2-yl)benzamide (**32**)

White solid. Yield: 86% (0.7 g); mp 165.8–166.5 °C; TLC: *R*_f_ = 0.48 (S_2_); UPLC (purity >99%): *t*_R_ = 6.98 min. LC-MS (ESI): *m*/*z* calcd for for C_21_H_22_N_3_O_2_F_3_ (M + H)^+^ 406.17, found 406.3. ^1^H NMR (500 MHz, CDCl_3_) δ 1.46 (d, *J* = 6.9 Hz, 3H, CH_3_), 3.16–3.33 (m, 4H, piperazine), 3.65–3.83 (m, 3H, piperazine), 3.89 (dd, *J* = 6.4, 3.7 Hz, 1H, piperazine), 5.14 (quin, *J* = 7.0 Hz, 1H, CH), 7.06 (dd, *J* = 8.3, 2.6 Hz, 1H, ArH), 7.10–7.16 (m, 2H, ArH), 7.33 (br d, *J* = 7.45 Hz, 1H, NH), 7.35–7.39 (m, 1H, ArH), 7.40–7.45 (m, 2H, ArH), 7.47–7.52 (m, 1H, ArH), 7.79–7.85 (m, 2H, ArH). ^13^C NMR (126 MHz, CDCl_3_) δ 19.3, 42.0, 45.3, 45.6, 49.0, 49.3, 113.1 (br d, *J* = 3.62 Hz), 117.1 (br d, *J* = 3.62 Hz), 119.6 124.3 (q, *J* = 272.0 Hz) 127.1, 128.7, 129.9, 131.7 (q, *J* = 32.0 Hz) 131.8, 134.1, 151.0, 166.5, 171.1.

#### General Method for the Preparation of Final Compounds **33**–**36**

Intermediate compound **12** or **16** (2 mmol, 1 equiv) was dissolved in 10 mL of DCM. Afterward, to the solution was added dropwise appropriate isocyanate derivatives (2 mmol, 1 equiv) at 0 °C in an ice bath. The reaction mixture was allowed to warm up to room temperature, stirred for 2 h, and evaporated to dryness. Then, column chromatography was applied for purification of crude products using developing system S_2_. The desired compounds were obtained as white powder, followed by the concentration of organic solvents under reduced pressure and wash-up by diethyl ether.

#### 1-(1-(4-(3-Chlorophenyl)piperazin-1-yl)-1-oxopropan-2-yl)-3-ethylurea (**33**)

White solid. Yield: 84% (0.56 g); mp 145.1–146.2 °C; TLC: *R*_f_ = 0.54 (S_2_); UPLC (purity >99%): *t*_R_ = 5.53 min. LC-MS (ESI): *m*/*z* calcd for C_16_H_23_N_4_O_2_Cl (M + H)^+^ 339.15, found 339.7. ^1^H NMR (500 MHz, CDCl_3_) δ 1.09 (t, *J* = 7.2 Hz, 3H, CH_3_), 1.30 (d, *J* = 6.9 Hz, 3H, CH_3_), 3.09–3.28 (m, 6H, CH_2_, piperazine), 3.59–3.72 (m, 2H, piperazine), 3.75–3.86 (m, 2H, piperazine), 4.86–4.96 (m, 1H, CH), 5.07 (br t, *J* = 5.2 Hz, 1H, NH), 5.90 (d, *J* = 8.0 Hz, 1H, NH), 6.73–6.80 (m, 1H, ArH), 6.82–6.89 (m, 2H, ArH), 7.16 (t, *J* = 8.3 Hz, 1H, ArH). ^13^C NMR (126 MHz, CDCl_3_) δ 15.6, 19.7, 35.2, 42.0, 45.4, 45.4, 48.9, 49.3, 114.6, 116.5, 120.4, 130.3, 135.2, 151.9, 157.6, 172.9.

#### 1-Ethyl-3-(1-oxo-1-(4-(3-(trifluoromethyl)phenyl)piperazin-1-yl)propan-2-yl)urea (**34**)

White solid. Yield: 88% (0.65 g); mp 136.4–137.3 °C; TLC: *R*_f_ = 0.56 (S_2_); UPLC (purity >99%): *t*_R_ = 5.90 min. LC-MS (ESI): *m*/*z* calcd for for C_17_H_23_N_4_O_2_F_3_ (M + H)^+^ 373.18, found 373.2. ^1^H NMR (500 MHz, CDCl_3_) δ 1.04–1.15 (m, 3H, CH_3_), 1.31 (d, *J* = 6.9 Hz, 3H, CH_3_), 3.11–3.32 (m, 6H, CH_2_, piperazine), 3.64–3.76 (m, 2H, piperazine), 3.77–3.90 (m, 2H, piperazine), 4.87–4.97 (m, 1H, CH), 5.03 (br t, *J* = 5.2 Hz, 1H, NH), 5.87 (d, *J* = 8.0 Hz, 1H, NH), 7.05 (dd, *J* = 8.0, 2.3 Hz, 1H, ArH), 7.08–7.15 (m, 2H, ArH), 7.36 (t, *J* = 7.7 Hz, 1H, ArH). ^13^C NMR (126 MHz, CDCl_3_) δ 15.6, 19.7, 35.2, 42.0, 45.4, 45.4, 48.9, 49.3, 112.9 (br d, *J* = 3.6 Hz), 117.0 (br d, *J* = 3.6 Hz) 119.5, 124.2 (q, *J* = 272.2 Hz), 129.8, 131.7 (q, *J* = 31.6 Hz), 151.0, 157.6, 172.9.

#### 1-(1-(4-(3-Chlorophenyl)piperazin-1-yl)-1-oxopropan-2-yl)-3-isopropylurea (**35**)

White solid. Yield: 85% (0.59 g); mp 147.4–148.2 °C; TLC: *R*_f_ = 0.56 (S_2_); UPLC (purity >99%): *t*_R_ = 5.72 min. LC-MS (ESI): *m*/*z* calcd for for C_17_H_25_N_4_O_2_Cl (M + H)^+^ 353.17, found 353.8. ^1^H NMR (500 MHz, CDCl_3_) δ 1.09 (d, *J* = 6.3 Hz, 3H, CH_3_), 1.12 (d, *J* = 6.3 Hz, 3H, CH_3_), 1.30 (d, *J* = 6.9 Hz, 3H, CH_3_), 3.08–3.27 (m, 4H, CH, piperazine), 3.63–3.73 (m, 2H, piperazine), 3.75–3.88 (m, 3H, piperazine), 4.86–4.95 (m, 2H, CH, NH) 5.82, (d, *J* = 8.0 Hz, 1H, NH), 6.74–6.80 (m, 1H, ArH), 6.82–6.88 (m, 2H, ArH), 7.17 (t, *J* = 8.02 Hz, 1H, ArH). ^13^C NMR (126 MHz, CDCl_3_) δ 19.8, 23.6 (d, *J* = 4.2 Hz,) 42.0, 42.1, 45.3, 45.4, 49.0, 49.3, 114.6, 116.5, 120.4, 130.3, 135.2, 151.9, 156.9, 172.9.

#### 1-Isopropyl-3-(1-oxo-1-(4-(3-(trifluoromethyl)phenyl)piperazin-1-yl)propan-2-yl)urea (**36**)

White solid. Yield: 90% (0.69 g); mp 144.5–145.4 °C; TLC: *R*_f_ = 0.56 (S_2_); UPLC (purity >99%): *t*_R_ = 6.09 min. LC-MS (ESI): *m*/*z* calcd for for C_18_H_25_N_4_O_2_F_3_ (M + H)^+^ 387.20, found 387.5. ^1^H NMR (500 MHz, CDCl_3_) δ 1.09 (d, *J* = 6.3 Hz, 3H, CH_3_), 1.13 (d, *J* = 6.3 Hz, 3H, CH_3_), 1.31 (d, *J* = 6.9 Hz, 3H, CH_3_), 3.15–3.30 (m, 4H, CH, piperazine), 3.66–3.76 (m, 2H, piperazine), 3.78–3.89 (m, 3H, piperazine), 4.86–4.96 (m, 2H, CH, NH), 5.82 (d, *J* = 8.0 Hz, 1H, NH), 7.05 (dd, *J* = 8.6, 2.3 Hz, 1H, ArH), 7.08–7.15 (m, 2H, ArH), 7.30–7.39 (m, 1H, ArH). ^13^C NMR (126 MHz, CDCl_3_) δ 19.7, 23.6 (d, *J* = 4.8 Hz) 42.0, 42.1, 45.3, 45.4, 49.0, 49.3, 112.9 (br d, *J* = 3.6 Hz), 117.0 (br d, *J* = 3.6 Hz), 119.5, 124.2 (q, *J* = 272.2 Hz), 129.8, 131.7 (q, *J* = 32.0 Hz), 151.0, 157.0, 173.0.

#### General Method for the Preparation of Intermediates **37**–**40**

Carbonyldiimidazole (0.51 g, 3.1 mmol, 1.2 equiv) was dissolved in 10 mL of DCM. Afterward, this solution was added to Boc-dl-alanine (0.5 g, 2,6 mmol, 1 equiv) and dissolved in 10 mL of DCM (while stirring). After 0.5 h, the respective pyrrolidin-3-amine derivatives (2.6 mmol, 1 equiv) dissolved in 5 mL of DCM were added dropwise. The mixture was stirred for approximately 3 h at room temperature and evaporated to dryness. The column chromatography was applied for purification of crude products using developing systems S_1_. The desired compounds were obtained as light oils, followed by the concentration of organic solvents under reduced pressure.

#### *tert*-Butyl (1-oxo-1-((1-Phenylpyrrolidin-3-yl)amino)propan-2-yl)carbamate (**37**)

Light yellow oil. Yield 88% (0.76 g); TLC: *R*_f_ = 0.81 (S_1_); UPLC (purity >99%): *t*_R_ = 6.67 min. LC-MS (ESI): *m*/*z* calcd for C_18_H_27_N_3_O_3_ (M + H)^+^ 334.21, found 334.3.

#### *tert*-Butyl (1-oxo-1-((1-(3-(Trifluoromethyl)phenyl)pyrrolidin-3-yl)amino)propan-2-yl) carbamate (**38**)

Light yellow oil. Yield 85% (0.9 g); TLC: *R*_f_ = 0.87 (S_1_); UPLC (purity >99%): *t*_R_ = 7.58 min. LC-MS (ESI): *m*/*z* calcd for C_19_H_26_N_3_O_3_F_3_ (M + H)^+^ 402.20, found 402.3.

#### *tert*-Butyl (1-oxo-1-((1-(3-(Trifluoromethoxy)phenyl)pyrrolidin-3-yl)amino)propan-2-yl)carbamate (**39**)

Light yellow oil. Yield 90% (0.97 g); TLC: *R*_f_ = 0.89 (S_1_); UPLC (purity >99%): *t*_R_ = 7.81 min. LC-MS (ESI): *m*/*z* calcd for C_19_H_26_N_3_O_4_F_3_ (M + H)^+^ 418.19, found 418.2.

#### *tert*-Butyl (1-oxo-1-((1-(3-((Trifluoromethyl)thio)phenyl)pyrrolidin-3-yl)amino)propan-2-yl)carbamate (**40**)

Yellow oil. Yield 87% (0.97 g); TLC: *R*_f_ = 0.89 (S_1_); UPLC (purity >99%): *t*_R_ = 8.22 min. LC-MS (ESI): *m*/*z* calcd for C_19_H_26_N_3_O_3_SF_3_ (M + H)^+^ 434.17, found 434.3.

#### General Method for the Preparation of Compounds **41**–**44**

The solution of **37**–**40** (2.2 mmol, 1 equiv) in DCM (10 mL) was treated with TFA (3 equiv) and stirred at room temperature for 3 h. Afterward, the organic solvents were evaporated to dryness. The resulting oil residue was dissolved in water (20 mL), and then 25% ammonium hydroxide was carefully added to pH = 8. The aqueous layer was extracted with DCM (3 × 20 mL), dried over Na_2_SO_4_, and concentrated to give **41**–**44** as yellow oils. Intermediates **41**–**44** were advanced as substrates without purification for the last reaction.

#### 2-Amino-*N*-(1-phenylpyrrolidin-3-yl)propanamide (**41**)

Yellow oil. Yield 96% (0.49 g); TLC: *R*_f_ = 0.20 (S_2_); UPLC (purity >99%): *t*_R_ = 3.15 min. LC-MS (ESI): *m*/*z* calcd for C_13_H_19_N_3_O (M + H)^+^ 234.16, found 234.2.

#### 2-Amino-*N*-(1-(3-(trifluoromethyl)phenyl)pyrrolidin-3-yl)propanamide (**42**)

Yellow oil. Yield 98% (0.65 g); TLC: *R*_f_ = 0.24 (S_2_); UPLC (purity >99%): *t*_R_ = 4.51 min. LC-MS (ESI): *m*/*z* calcd for C_14_H_18_N_3_OF_3_ (M + H)^+^ 302.14, found 302.2.

#### 2-Amino-*N*-(1-(3-(trifluoromethoxy)phenyl)pyrrolidin-3-yl)propanamide (**43**)

Yellow oil. Yield 96% (0.67 g); TLC: *R*_f_ = 0.25 (S_2_); UPLC (purity >99%): *t*_R_ = 5.02 min. LC-MS (ESI): *m*/*z* calcd for C_14_H_18_N_3_O_2_F_3_ (M + H)^+^ 318.13, found 318.2.

#### 2-Amino-*N*-(1-(3-((trifluoromethyl)thio)phenyl)pyrrolidin-3-yl)propanamide (**44**)

Yellow oil. Yield 97% (0.71 g); TLC: *R*_f_ = 0.25 (S_2_); UPLC (purity >99%): *t*_R_ = 5.40 min. LC-MS (ESI): *m*/*z* calcd for C_14_H_18_N_3_OSF_3_ (M + H)^+^ 334.12, found 334.1.

#### General Method for the Preparation of Final Compounds **45**–**48**

Intermediates **41**–**44** (2 mmol, 1 equiv) were dissolved in 10 mL of DCM. Afterward, triethylamine (TEA, 6 mmol, 3 equiv) was added while stirring at 0 °C to the solution. Final compounds **45**–**48** were prepared by dropwise adding acetyl chloride (3 mmol, 1.5 equiv) at 0 °C in an ice bath. The reaction mixture was allowed to warm up to room temperature, stirred for an additional 2 h, and evaporated to dryness. Next, the crude product was purified by applying column chromatography using developing system S_2_. The desired compounds were obtained as white solids, followed by the concentration of organic solvents under reduced pressure and wash-up by diethyl ether.

#### 2-Acetamido-*N*-(1-phenylpyrrolidin-3-yl)propanamide (**45**)

White solid. Yield: 92% (0.51 g); mp 176.6–177.8 °C; TLC: *R*_f_ = 0.46 (S_2_); UPLC (purity = 98.1%): *t*_R_ = 4.29 min. LC-MS (ESI): *m*/*z* calcd for C_15_H_21_N_3_O_2_ (M + H)^+^ 276.17 found 276.3. ^1^H NMR (500 MHz, CDCl_3_) δ 1.31 (t, *J* = 7.3 Hz, 3H, CH_3_), 1.85–1.92 (m, 3H, CH_3_), 1.93–2.01 (m, 1H, pyrrolidine), 2.24 (m, 1H, pyrrolidine), 3.16 (td, *J* = 9.0, 3.7 Hz, 1H, pyrrolidine), 3.25–3.36 (m, 1H, pyrrolidine), 3.38–3.47 (m, 1H, pyrrolidine), 3.54 (dd, *J* = 6.1, 3.6 Hz, 1H, pyrrolidine), 4.45–4.58 (m, 2H, pyrrolidine, CH), 6.48–6.58 (m, 3H, NH, ArH), 6.68 (td, *J* = 7.2, 4.2 Hz, 1H, ArH), 7.17–7.24 (m, 2H, ArH), 7.27 (br t, *J* = 8.3 Hz, 1H, ArH). ^13^C NMR (126 MHz, CDCl_3_) δ 18.8, 23.2, 31.6, 45.9, 48.8, 49.5, 53.4, 111.9, 116.3, 129.3, 147.6, 170.2, 172.4.

#### 2-Acetamido-*N*-(1-(3-(trifluoromethyl)phenyl)pyrrolidin-3-yl)propanamide (**46**)

White solid. Yield: 91% (0.62 g); mp 162.8–163.7 °C; TLC: *R*_f_ = 0.54 (S_2_); UPLC (purity = 98.7%): *t*_R_ = 5.61 min. LC-MS (ESI): *m*/*z* calcd for C_16_H_20_N_3_O_2_F_3_ (M + H)^+^ 344.15 found 344.3. ^1^H NMR (500 MHz, CDCl_3_) δ 1.28–1.33 (m, 3H, CH_3_), 1.81–1.91 (m, 3H, CH_3_), 1.94–2.07 (m, 1H, pyrrolidine), 2.21–2.32 (m, 1H, pyrrolidine), 3.19 (td, *J* = 10.6, 3.7 Hz, 1H, pyrrolidine), 3.28–3.39 (m, 1H, pyrrolidine), 3.40–3.48 (m, 1H, pyrrolidine), 3.58 (dt, *J* = 9.9, 6.2 Hz, 1H, pyrrolidine), 4.43–4.61 (m, 2H, pyrrolidine, CH), 6.50 (br dd, *J* = 15.9, 7.6 Hz, 1H, NH), 6.61–6.67 (m, 1H, ArH), 6.69 (br d, *J* = 6.6 Hz, 1H, ArH), 6.87–6.94 (m, 1H, ArH), 7.25–7.30 (m, 1H, ArH), 7.43 (br t, *J* = 6.9 Hz, 1H, NH). ^13^C NMR (126 MHz, CDCl_3_) δ 18.8, 23.1, 31.4, 45.9, 48.8, 49.5, 53.4, 108.0, (br d, *J* = 4.2 Hz), 112.5 (br d, *J* = 3.6 Hz), 114.8, 124.5 (q, *J* = 272.2 Hz), 129.7, 131.5 (q, *J* = 32.2 Hz), 147.5, 170.2, 172.5.

#### 2-Acetamido-*N*-(1-(3-(trifluoromethoxy)phenyl)pyrrolidin-3-yl)propanamide (**47**)

White solid. Yield: 92% (0.66 g); mp 164.8–165.9 °C; TLC: *R*_f_ = 0.57 (S_2_); UPLC (purity >99%): *t*_R_ = 5.84 min. LC-MS (ESI): *m*/*z* calcd for C_16_H_20_N_3_O_3_F_3_ (M + H)^+^ 360.15 found 360.2. ^1^H NMR (500 MHz, CDCl_3_) δ 1.28–1.34 (m, 3H, CH_3_), 1.83–1.90 (m, 3H, CH_3_), 1.99 (dt, *J* = 12.7, 5.1 Hz, 1H, pyrrolidine), 2.20–2.31 (m, 1H, pyrrolidine), 3.15 (td, *J* = 10.2, 3.9 Hz, 1H, pyrrolidine), 3.26–3.35 (m, 1H, pyrrolidine), 3.37–3.45 (m, 1H, pyrrolidine), 3.54 (dt, *J* = 10.0, 6.2 Hz, 1H, pyrrolidine), 4.46–4.57 (m, 2H, pyrrolidine, CH), 6.32 (br d, *J* = 6.9 Hz, 1H, ArH), 6.41 (dd, *J* = 6.2, 2.0 Hz, 1H, ArH), 6.47 (br d, *J* = 7.7 Hz, 1H, NH), 6.49–6.55 (m, 1H, ArH), 7.17 (td, *J* = 8.2, 3.7 Hz, 1H, ArH), 7.42 (br t, *J* = 7.6 Hz, 1H, NH). ^13^C NMR (126 MHz, CDCl_3_) δ 18.8, 23.1, 31.4, 45.9, 48.8, 49.5, 53.4, 104.8, 108.0, 110.1, 120.6 (q, *J* = 256.1 Hz), 130.2, 148.7, 150.5, 170.2, 172.5.

#### 2-Acetamido-*N*-(1-(3-((trifluoromethyl)thio)phenyl)pyrrolidin-3-yl)propanamide (**48**)

White solid. Yield: 89% (0.67 g); mp 162.2–163.3 °C; TLC: *R*_f_ = 0.58 (S_2_); UPLC (purity >99%): *t*_R_ = 6.22 min. LC-MS (ESI): *m*/*z* calcd for C_16_H_20_N_3_O_2_SF_3_ (M + H)^+^ 376.13 found 376.3. ^1^H NMR (500 MHz, CDCl_3_) δ 1.27–1.34 (m, 3H, CH_3_), 1.82–1.89 (m, 3H, CH_3_), 1.93–2.05 (m, 1H, pyrrolidine), 2.21–2.32 (m, 1H, pyrrolidine), 3.17 (td, *J* = 10.5, 3.9 Hz, 1H, pyrrolidine), 3.32 (tt, *J* = 8.7, 5.6 Hz, 1H, pyrrolidine), 3.39–3.47 (m, 1H, pyrrolidine), 3.56 (dt, *J* = 10.0, 6.0 Hz, 1H, pyrrolidine), 4.47–4.58 (m, 2H, pyrrolidine, CH), 6.49 (br dd, *J* = 17.3, 7.6 Hz, 1H, NH), 6.57–6.63 (m, 1H, ArH), 6.75 (br d, *J* = 6.9 Hz, 1H, ArH), 6.93 (dd, *J* = 7.5, 3.4 Hz, 1H, ArH), 7.21 (td, *J* = 7.9, 3.4 Hz, 1H, ArH), 7.44 (br t, *J* = 7.45 Hz, 1H, NH). ^13^C NMR (126 MHz, CDCl_3_) δ 18.8, 23.1, 31.4, 45.9, 48.8, 49.5, 53.3, 114.0, 118.9, 123.6, 125.0, 129.9 (q, *J* = 308.0 Hz), 130.1, 148.0, 170.2, 172.5.

### 4.2 *In Silico* Studies

Lipinski’s rule of five (RO5) parameters, i.e., molecular weight (MW), lipophilicity (log *P*), number of hydrogen bond donors (NHD), and number of hydrogen bond acceptors (NHA), as well as Veber’s rule, i.e., number of rotatable bonds (NBR) and polar surface area (TPSA), were calculated using the SwissAdme software.^26^ Central Nervous System Multi-Parameter Optimization (CNS MPO) parameters were determined using Instant JChem 21.4.0 software (ChemAxon).

### 4.3 Antiseizure Activity

#### 4.3.1 Animals

The adult male Albino Swiss mice weighed between 22 and 26 g were used in the *in vivo* studies. They were housed under standardized housing conditions in colony cages and had free access to food as well as tap water. The animals were left to adapt under laboratory conditions for 7 days. All procedures involving animals and their care were performed in accordance with the current European Community and Polish legislation on animal experimentation. The studies were carried out under experimental protocols approved by the Local Ethics Committee in Lublin (144/2018, 85/2019, 25/2021, 122/2022, 29/2023 and 13/2021, 46/2021), in accordance with the European Communities Council Directive of 22 Sept 2010 (2010/63/EU).

#### 4.3.2 Antiseizure Activity and Acute Neurotoxicity

In the initial screening studies, four mice per group were randomly assigned to each experimental group (each mouse was used only once). To evaluate the ED_50_ or TD_50_ values, four groups consisting of eight animals were injected with various doses of tested compounds. The protective indexes (PIs) for the compounds investigated and reference ASMs were calculated by dividing the TD_50_ value, as determined in the chimney test, by the respective ED_50_ value, as determined in the MES, *sc*PTZ, or 6 Hz (32 or 44 mA) tests (PI = TD_50_/ED_50_). The PIs are a measure of the potential therapeutic window of the tested agent.

All substances were suspended in Tween 80 (1% aqueous solution) and administered *i.p.* as a single injection at a dose of 10 mL/kg. On each day of experimentation, fresh solutions were prepared. The detailed *in vivo* procedures are described elsewhere: the maximal electroshock seizure test (MES),^60^ the subcutaneous pentylenetetrazole seizure test (*sc*PTZ),^61^ the 6 Hz psychomotor seizure model (32 and 44 mA),^62^ and the chimney test.^63^ The reference ASMs were purchased from commercial suppliers: VPA (Sigma-Aldrich, St. Louis, MO), LCS, and LEV (UCB Pharma, Braine l’Alleud, Belgium).

#### 4.3.3 Timed *iv*PTZ Seizure Threshold and Grip Strength Tests

In studies assessing the acute effect of compounds **26** and **28** on the ivPTZ seizure threshold and neuromuscular strength, the compounds were suspended in a 1% solution of Tween 80 and administered *i.p.*, at a dose of 50 mg/kg of body weight, 30 min before the tests. The experimental procedures of the timed ivPTZ test and the grip strength test were described in detail elsewhere.^64^

Data from the *iv*PTZ test and the grip strength test were analyzed using Student’s *t*-test.

#### 4.3.4 PTZ-Induced Kindling in Mice

The procedure was performed as described in detail in our earlier studies.^15,65^ Briefly, compound **28** was suspended in 1% Tween 80, while VPA (sodium salt) and PTZ were dissolved in saline. Compound **28**, VPA, or vehicle were injected *i.p.* every 24 h. PTZ (40 mg/kg, *i.p.*) was given three times a week (Mon, Wed, Fri), 30 min after administration of compound **28**, VPA, or vehicle. The seizure severity was scored using the modified Racine’s scale. Experimental grouping was as follows: (a) 1% Tween + saline (nonkindled control), (b) 1% Tween + PTZ (PTZ-kindled control), (c) VPA at 150 mg/kg + PTZ (positive control), and (d)–(f) PTZ + compound **28** at 10, 20, and 40 mg/kg. 24 h after the last PTZ injection, animals were subjected to the locomotor activity test, the elevated plus maze test, and the forced swim test according to the methods described in detail elsewhere. After behavioral tests, animals were sacrificed and the brains were rapidly removed. Hippocampi and cortices were dissected, frozen, and stored at −80 °C until the assay of determination of GABA and glutamate concentration.

The mean seizure severity scores were calculated for all experimental groups after each PTZ injection and analyzed using a mixed-effect model for repeated measures with Tukey’s post hoc test. Fisher’s exact probability test was used to compare the percentage of fully kindled mice.

#### 4.3.5 Determination of GABA and Glutamate Concentrations in Murine Brain Structures

Concentrations of GABA and glutamate in the mouse prefrontal cortex and hippocampus were measured by the liquid chromatography-tandem mass spectrometry (LC-MS/MS) method. Standards of both analytes were purchased from Toronto Research Chemicals Inc. (Toronto, ON, Canada). The stock standard solutions of GABA and glutamate were prepared in methanol and deionized water, respectively, and stored at 4 °C. A series of solution mixtures of desired concentrations were prepared by suitable dilutions of the stock solutions. Before analysis, murine brains were homogenized in distilled water at the ratio of 50 μL/mg using a handheld pestle and a glass tube homogenizer (Potter–Elvehjem PTFE pestle and glass tube, Sigma-Aldrich). Homogenates were centrifuged at 8000*g* for 10 min at 4 °C, and the supernatant was diluted 10 times with 0.1% formic acid in MeCN. After addition of isotope-labeled GABA-*d*_6_ and glutamate-*d*_5_ (Toronto Research Chemicals Inc., Toronto, ON, Canada) as internal standards (10 μL at a concentration of 500 ng/mL), samples (10 μL) were deproteinized with 80 μL of 0.1% formic acid in MeCN by shaking for 10 min (IKA Vibrax VXR, Germany) and then centrifuged for 5 min at a speed of 8000*g* (Eppendorf miniSpin centrifuge). The obtained supernatants were transferred into the autosampler vials. Chromatographic separation was carried out on an XBridge HILIC analytical column (2.1 × 150 mm^2^, 3.5 μm, Waters, Ireland) with the oven temperature set at 25 °C using the Excion LC AC HPLC system. The autosampler temperature was maintained at 15 °C, and a sample volume of 2 μL was injected into the LC-MS/MS system. The mobile phase containing 0.1% formic acid in acetonitrile and 0.1% formic acid in water was mixed at a ratio of 70:30 and run at 0.3 mL/min. Mass spectrometric detection was performed on a Sciex QTRAP 4500 triple quadrupole mass spectrometer. Electrospray ionization (ESI) in the positive ion mode was used for ion production. The tandem mass spectrometer was operated at unit resolution in the selected reaction monitoring mode (SRM), monitoring the transition of the protonated molecular ions *m*/*z* 104–87 and *m*/*z* 104–69 for GABA and *m*/*z* 148–84 and *m*/*z* 148–102 for glutamate (the first pair was used as a quantifier and the second for the identity verification—qualifier). For isotope-labeled GABA-*d*_6_ and glutamate-*d*_5_ monitored pairs were *m*/*z* 110–93 and *m*/*z* 153–88, respectively. The mass spectrometric conditions were optimized for GABA and glutamate by continuous infusion of the standard solution at a rate of 7 μL/min using a Harvard infusion pump. The ion source temperature was maintained at 450 °C. The ion spray voltage was set at 5000 V. The curtain gas (CUR) was set at 40 psi and the collision gas (CAD) at Medium. Data acquisition and processing were accomplished using Applied Biosystems Analyst version 1.7 software. The calibration curves were constructed by plotting the ratio of the peak area of the studied compound to the internal standard versus drug concentration and generated by weighted (1/*x* × *x*) linear regression analysis. Due to the high endogenous concentrations of GABA and glutamate and the availability of stable isotope standards, calibration curves were constructed based on serial dilutions of the calibrators in water. The validated quantitation ranges for this method were within the expected concentration ranges, namely, from 100 to 5000 μg/g of brain tissue with accuracy from 90.89 to 108.43% and from 90.48 to 111.36% for GABA and glutamate, respectively. No significant matrix effect was observed, and there were no stability-related problems during the routine analysis of the samples.

Changes in glutamate and GABA concentration were analyzed using one-way ANOVA with Tukey’s post hoc test.

#### 4.3.6 BNDF and proNGF Expressions in the Hippocampus and Cortex of PTZ-Kindled Mice

The expression of mBDNF and proNGF in the mouse hippocampus and cortex was evaluated by the western blot technique. Half of mouse brain structures, cortex and hippocampus, were weighted and homogenized at the ratio of 9 μL/mg in 2% sodium dodecyl sulfate (SDS) supplemented with a cocktail of protease (Thermo Scientific) and phosphatese inhibitors (Sigma-Aldrich) using a bead homogenizer (Bead Ruptor Elite, Omni International). Then, the samples were denatured at 95 °C for 10 min and centrifuged at 10,000*g* for 10 min at 4 °C. The total protein concentration in the obtained supernatants was determined using a PierceTM BCA Protein Assay Kit (Thermo Scientific, Waltham, MA). After setting the proper protein concentration, the samples were mixed with loading buffer (containing 10% 2-mercaptoethanol) at a ratio of 3:1 and heated for 10 min at 95 °C. Equal amounts of the protein (45 μg/lane) were loaded on Any kD precast polyacrylamide gels (Criterion, TGX Stain-Free gel, Bio-Rad, Hercules, CA) and subjected to electrophoresis (170 V, 60 min). Separated proteins were transferred to the polyvinylidene difluoride (PVDF) membrane (Bio-Rad, Hercules, CA) and were blocked in a 5% solution of albumin in TBST. The membranes were incubated overnight at 4 °C with primary antibodies: rabbit monoclonal anti-BDNF (ab108319, Abcam, 1:1000) and rabbit polyclonal anti-NGF (ab6199, Abcam, 1:1000), followed by the secondary goat antirabbit IgG peroxidase-conjugated antibody (ab205718, Abcam, 1:5000). The proteins were detected using the electrochemiluminescence (ECL) method (Western Bright Quantum, Advansta Inc., San Jose, CA). The chemiluminescence of the membranes was imaged with the G-Box Imaging System (Syngene, Frederick, MD), and the protein expression was analyzed with Gene Tools software (Syngene, Frederick, MD) and expressed as relative to the total protein content in the membrane lane.

Changes in the relative expression of tested proteins were analyzed using one-way ANOVA with Tukey’s post hoc test.

### 4.4 Antinociceptive Activity

The experimental groups consisted of 10 adult male Albino Swiss mice (CD-1, 18–25 g). Each animal was tested only once. Immediately after the assay, the animals were sacrificed by cervical dislocation. Behavioral measurements were observed by trained observers. The *in vivo* antinociceptive assays were in accordance with Polish regulations and the European Union Directive of 22 Sept 2010 (2010/63/EU). All procedures were carried out according to the rules of the International Council on Laboratory Animal Science (ICLAS) and were approved by the Local Ethics Committee in Cracow, Poland (104/2015, 279/2019, and 614/2022). The tested substances were suspended in a 1% aqueous solution of Tween 80 and were injected *i.p.* 30 min prior to the test. Control group animals (negative control) were administered with an appropriate amount of vehicle (Tween 80, 1% aqueous solution, *i.p.*) 30 min prior to the test. The experimental *in vivo* procedures were previously reported for the formalin test;^66^ compound **28** was tested in three doses of 12.5, 25, and 50 mg/kg. Before formalin application, different groups of animals were injected *i.p.* with vehicle (10 mL/kg, negative control). The *in vivo* procedure for the model of capsaicin-induced nociception was previously reported;^67^ the animals were pretreated with vehicle (10 mL/kg, negative control), and the dose–response of investigated compounds was evaluated at 12.5, 25, and 50 mg/kg. The *in vivo* procedure for the model of OXPT-induced peripheral neuropathy was previously reported;^68^ the mice with developed tactile allodynia were pretreated *i.p.* with test compound **28** (25, 50, and 75 mg/kg) and vehicle. The *in vivo* procedure of streptozotocin-induced hyperglycemia was previously reported;^69^ the mice with developed mechanical allodynia were pretreated *i.p.* with test compound **28** (12.5, 25, and 50 mg/kg) and vehicle.

Data are presented as means ± standard error of the mean (SEM). GraphPad Prism Software (v.5) was used to analyze the vast majority of data. Statistically significant differences between groups were calculated using one-way analysis of variance (ANOVA) and the post hoc Dunnett’s multiple comparison test or two-way analysis of variance (ANOVA). The criterion for significance was set at *p* < 0.05. The log-probit method was applied to statistically determine the ED_50_ values with 95% confidence limits.

### 4.5 Pharmacokinetic Study

#### 4.5.1 Animals and Study Design

Male CD-1 mice weighing 28–33 g housed in conditions of constant temperature with the 12:12 h light–dark cycle with free access to food and water were used in this study. The investigated compound was suspended in 1% Tween in sterile water for injection (Polpharma, Poland) and administered *i.p.* at two doses of 25 and 50 mg/kg. Additionally, the compound dissolved in a mixture of dimethylsulfoxide (DMSO), poly(ethylene glycol), and sterile water (1:2:7, v/v/v) was given orally (*p.o.*) at a dose of 25 mg/kg. The mice were sacrificed by decapitation under isoflurane anesthesia. Blood samples were collected at 5, 15, 30, 60, 120, 240, and 720 min after dosing (*n* = 3–4), and brains were harvested at the same time points. Blood was allowed to clot at room temperature for 20 min, and serum was separated by centrifugation at 3000 rpm for 10 min. The samples were stored at −80 °C until analysis. All animal procedures were approved by the First Ethics Committee on Animal Experimentation in Kraków (license no. 270/2019).

#### 4.5.2 Analytical Method

Concentrations of compound **28** in murine serum and brain tissue were measured by the liquid chromatography–tandem mass spectrometry method (LC-MS/MS). Before LC-MS/MS analysis, mouse brains were homogenized in distilled water at the ratio of 1:4 (w/v) using the ULTRA-TURRAX T10 basic tissue homogenizer (IKA, Germany). Brain homogenates or serum samples (50 μL) were deproteinized with 150 μL of 0.1% formic acid in acetonitrile with addition of the internal standard (valsartan), shaken for 10 min (IKA Vibrax VXR, Germany), and then centrifuged for 5 min at a speed of 8000*g* (Eppendorf miniSpin centrifuge, Germany). Supernatants were transferred into autosampler vials, and a sample volume of 1 μL was injected into the LC-MS/MS system. The autosampler temperature was maintained at 15 °C. Analytes were separated on a Hypersil Gold C18 analytical column (3 × 50 mm^2^, 5 μm, Thermo Scientific) using an AB Sciex Exion LC AC HPLC system coupled with the triple quadrupole mass spectrometer (Sciex QTRAP 4500, both from the Danaher Corporation). The oven temperature was set at 30 °C. The initial mobile phase composition was 95% B (0.1% formic acid in water) and 5% A (0.1% formic acid in MeCN) for the first 2 min with a linear gradient to 5% B in the next 2 min and then isocratic mode for 2 min with the following rapid change back to 95% B in 0.1 min. The remaining time of elution was set at 95% B. The whole HPLC operation lasted 10 min, and the flow rate was set at 0.4 mL/min. Electrospray ionization (ESI) in the positive ion mode was used for ion production. The tandem mass spectrometer was operated at unit resolution in the selected reaction monitoring mode (SRM), monitoring the transition of the protonated molecular ions *m*/*z* 360–247 (CE = 25 eV) and *m*/*z* 360–204 (CE = 49 eV) for compound **28** (the first pair was used as a quantifier and the second for the identity verification—qualifier) and *m*/*z* 436–207 (CE = 42 eV) for an internal standard. The mass spectrometric conditions were optimized by continuous infusion of the standard solution at a rate of 7 μL/min using a Harvard infusion pump. The ion source temperature was maintained at 450 °C, and the ion spray voltage was set at 5500 V. The curtain gas (CUR) was set at 40 psi and the collision gas (CAD) at Medium. The calibration curves were constructed by plotting the ratio of the peak area of the studied compound to the internal standard (IS) versus drug concentration and generated by weighted (1/*x* × *x*) linear regression analysis. The stock solution of compound **28** was prepared in methanol at a concentration of 1 mg/mL. Working standard solutions were prepared in methanol by the serial dilution of the stock solution at the following concentrations: 0.01, 0.1, 0.25, 0.5, 1, 2.5, 5, 10, 20, 50, 100, and 200 μg/mL. To prepare samples for the calibration curve, 45 μL of the matrix (plasma or brain homogenate) was spiked with 5 μL of the standard working solution at an appropriate concentration level and vortexed for 10 s. For each matrix, there were two calibration curves constructed separately for lower and higher concentrations. Calibrators (as well as samples) from the upper concentration range were additionally 10 times diluted with the precipitating agent. The validated quantitation ranges for this method were from 0.001 to 5 μg/mL and from 0.1 to 20 μg/mL of serum, and from 0.005 to 25 μg/g and from 0.5 to 100 μg/g of the brain. Samples with the concentration of **28** above the upper limit of quantification were diluted 10 times with the blank matrix (serum or brain homogenate). Calculated values of accuracy and precision were within the limits set by the FDA guidelines for the validation of bioanalytical methods. No significant matrix effect was observed, and there were no stability-related problems during the routine analysis of the samples. Data acquisition and processing were accomplished using Analyst version 1.7 software. The extracted ion chromatogram (serum sample) of compound **28** (*m*/*z* 360/247 and 360/204) and internal standard (*m*/*z* 436/207) is presented in Figure S12.

#### 4.5.3 Pharmacokinetic Data Analysis

To assess pharmacokinetic parameters, the noncompartmental approach was used. The peak concentration (*C*_max_) and the time to reach peak concentration (*t*_max_) in serum and brain tissue were obtained directly from the concentration versus time data. The terminal elimination rate constant (λ_*z*_) was estimated by means of linear regression, and terminal half-life (*t*_0.5λ*z*_) was calculated as ln 2/λ_*z*_. Clearance (CL/*F*) was calculated as dose/AUC_0–∞_, where AUC_0–∞_ is the area under concentration versus time curve from the time of dosing to infinity calculated by the linear trapezoidal rule. The extrapolated terminal area was defined as *C_n_*/λ_*z*_, where *C_n_* is the last data point. The volume of distribution based on the terminal phase (*V*_*z*_/*F*) was estimated as *D*/(λ_*z*_ × AUC_0–∞_), where *F* is fraction absorbed, and mean residence time (MRT) as AUC_0–∞_/AUMC_0–∞_, where AMUC_0–∞_ is the area under the first moment curve from the time of dosing to infinity.

### 4.6 *In Vitro* ADME-Tox Studies

All assays and protocols used for evaluation of compounds **26** and **28** ADME-Tox parameters were described previously.^16−18,70−74^ Precoated PAMPA Plate System Gentest used for estimation of passive permeability was provided by Corning, (Tewksbury, MA). The metabolic stability assay was performed on human liver microsomes (HLMs) purchased from Sigma-Aldrich (St. Louis, MO). The studies with microsomes were supported by MetaSite 6.0.1 software provided by Molecular Discovery Ltd. (Hertfordshire, U.K.), which allowed for determination of the most probable sites of metabolism. The plasma protein binding (PPB) studies were performed with the use of the commercial TRANSIL^XL^ PPB Assay (Sovicell, Leipzig, Germany). To predict potential drug–drug interactions (DDIs), the influence on recombinant human cytochromes CYP3A4 and CYP2D6 were carried with the use of CYP3A4 and CYP2D6 P450-Glo kits provided by Promega (Madison, WI). Cell-based safety tests were performed with a hepatoma HepG2 (ATCC HB-8065) cell line obtained directly from ATCC (American Type Culture Collection, Manassas, VA). The CellTiter 96 AQueous Non-Radioactive Cell Proliferation Assay (Promega, Madison, WI) was used for determination of cell viability after 48 h of incubation with serial dilutions of **26**, **28**, or the reference drug doxorubicin (DOX). The luminescence signal and the absorbances (measured at 490 nm) in DDIs and safety assays were measured by using a microplate reader EnSpire PerkinElmer (Waltham, MA). The LC/MS/MS analyses used in PAMPA, PPB, and metabolic stability assays were obtained on the Waters ACQUITY TQD system (Waters, Milford, CT). All reference drugs used (caffeine, ketoconazole, quinidine, doxorubicin, and verapamil) were purchased from Sigma-Aldrich (St. Louis, MO).

### 4.7 Binding/Functional Studies

#### Binding/Functional

Binding/functional studies were carried out commercially in Eurofins Laboratories (Poitiers, France) and Eurofins Panlabs Discovery Services Taiwan, Ltd. (New Taipei City, Taiwan) using testing procedures that were reported previously (for details, see Table S2).

#### Patch-Clamp

Patch-clamp studies in prefrontal cortex pyramidal neurons. The methodology of slice preparation, preparation of dispersed cortical neurons, and sodium current recording technique were the same as in our previous study.^75^ Compound **28** was tested at a concentration of 10 μM and was applied to the whole bath.

## Supporting Information Available

Synthetic procedure; physicochemical and spectral data for the starting amines; antiseizure screening data; binding/functional assay information; in silico metabolic biotransformation pathways; metabolism data (ion fragment analysis and structures of probable metabolites); ^1^H NMR and ^13^C NMR spectra for target compounds; and HRMS spectra for selected molecules (PDF)

## Abbreviations:

ADME-Tox: absorption, distribution, metabolism, excretion, toxicity
ASMs: antiseizure medications
BBB: blood–brain barrier
CDI: carbonyldiimidazole
CNS: central nervous system
DCM: dichloromethane
DDIs: drug–drug interactions
DRE: drug-resistant epilepsy
DOX: doxorubicin
GABA: γ-aminobutyric acid
HLMs: human liver microsomes
HRMS: high-resolution mass spectrometry
Six Hz: six-Hertz seizure test
KE: ketoconazole
LC-MS: liquid chromatography–mass spectrometry
LCS: lacosamide
MeCN: acetonitrile
MES: maximal electroshock seizure test
MeOH: methanol
OXPT: oxaliplatin
QD: quinidine
PI: protective index (TD_50_/ED_50_)
PTZ: pentylenetetrazole
*sc*PTZ: subcutaneous pentylenetetrazole seizure test
STZ: streptozotocin
TFA: trifluoroacetic acid
TRPV1: transient receptor potential cation channel vanilloid type 1
UPLC: ultraperformance liquid chromatography
VPA: valproic acid
