# Supplementary material for: Novel Alaninamide Derivatives with Drug-like Potential for Development as Antiseizure and Antinociceptive Therapies—In Vitro and In Vivo Characterization
Source: ACS Chem Neurosci. 2024 May 14;15(11):2198–222. doi: 10.1021/acschemneuro.4c00013 (PMC11157491; doi:10.1021/acschemneuro.4c00013)

Supporting Information for

Novel alaninamide derivatives with drug-like potential for development as antiseizure and antinociceptive therapies - *in vitro* and *in vivo* characterization

Marcin Jakubiec [1], Michał Abram [1], Mirosław Zagaja [2], Marta Andres-Mach [2], Joanna Szala-Rycaj [2], Gniewomir Latacz [3], Ewelina Honkisz-Orzechowska [3], Szczepan Mogilski [4], Monika Kubacka [4] Małgorzata Szafarz [5], Krzysztof Pociecha [5], Katarzyna Przejczowska-Pomierny [5], Elżbieta Wyska [5], Katarzyna Socała [6], Dorota Nieoczym [6], Bartłomiej Szulczyk [7], Piotr Wlaź [6], Cameron S. Metcalf [8], Karen Wilcox [8], Rafał M. Kamiński [1] and Krzysztof Kamiński [1,*]

[1] Department of Medicinal Chemistry, Faculty of Pharmacy, Jagiellonian University Medical College, Medyczna 9, 30-688 Krakow, Poland;

[2] Department of Experimental Pharmacology, Institute of Rural Health, Jaczewskiego 2, 20-950 Lublin, Poland;

[3] Department of Technology and Biotechnology of Drugs, Faculty of Pharmacy, Jagiellonian University Medical College, Medyczna 9, 30-688 Krakow, Poland;

[4] Department Pharmacodynamics, Faculty of Pharmacy, Jagiellonian University Medical College, Medyczna 9, 30-688 Krakow, Poland;

[5] Department of Pharmacokinetics and Physical Pharmacy, Faculty of Pharmacy, Jagiellonian University Medical College, Medyczna 9, 30-688 Krakow, Poland;

[6] Department of Animal Physiology and Pharmacology, Institute of Biological Sciences, Faculty of Biology and Biotechnology, Maria Curie-Skłodowska University, Akademicka 19, 20-033 Lublin, Poland;

[7] Chair and Department of Pharmacotherapy and Pharmaceutical Care, Centre for Preclinical Research and Technology, Medical University of Warsaw, Banacha 1B, 02-097 Warsaw, Poland

[8] Department of Pharmacology and Toxicology, University of Utah, Salt Lake City, UT 84112, USA

*Corresponding author:

Krzysztof Kamiński, Jagiellonian University Medical College, Faculty of Pharmacy, Department of Medicinal Chemistry, Medyczna 9, 30-688 Krakow, Poland.
E-mail: k.kaminski@uj.edu.pl

Table of contests

The procedure for synthesis, physicochemical and spectra data for A1-A8 3

The procedure for synthesis, physicochemical and spectra data for A9-A16 5

Table S1. Antiseizure activity screening data for compounds from **Series 1**, **2** and **3** in mice *i.p.* (100 mg/kg) 7

Table S2. Radioligand binding and functional assays 8

Table S3. The most probable metabolic pathways of **26** and **28** determined *in silico* and *in vitro* 9

Figure S1. Acute effects of compound **26** (**A**) and **28** (**B**) on grip strength in mice 10

Figure S2. Behavioral tests used to evaluate the effect of compound **28**. 10

Figure S3. The effect of compound **28** on the spontaneous locomotor activity in mice 11

Figure S4. Mean (±SD) serum and brain concentrations of **28** after *i.p.* administration of this compound at two doses 25 mg/kg (**A**) and 50 mg/kg (**B**) to mice (n=3-4) 12

Figure S5. Mean (±SD) serum and brain concentrations of **28** after *p.o.* administration at a dose of 25 mf/kg to mice (n=3-4) 13

Figure S6. MetaSite prediction of the most probable site of **26** metabolism 14

Figure S7. MetaSite prediction of the most probable site of **28** metabolism 14

Figure S8. UPLC of the reaction mixture after incubation of **26** with HLMs for 120 min 15

Figure S9. UPLC of the reaction mixture after incubation of **28** with HLMs for 120 min 15

Figure S10. MS spectra of compound **26** and its metabolites 16

Figure S11. MS spectra of compound **28** and its metabolites 17

Figure S12. Extracted ions chromatogram of compound **28** 18

References.. 19

HMRS traces for selected final compounds 20

^{1}H NMR and ^{13}C NMR spectra for the final compounds 34

The procedure for synthesis, physicochemical and spectra data for A1-A8

General method for the preparation of intermediates A1–A4. The starting (non-commercial) Boc-derivatives of 4-aryl piperazine were obtained by the *N*-arylation reaction of appropriate aryl bromide (10 mmol, 1 eq), Pd_2dba_3 (0.37 g, 0.4 mmol, 0.04 eq), BINAP (0.37 g, 0.6 mmol, 0.06 eq), sodium tert-butoxide (1.35 g, 14 mmol, 1.4 eq), and Boc-piperazine (3.74 g, 20 mmol, 2 eq) were suspended under an inert gas (nitrogen) atmosphere in 50 mL of dry toluene. The reaction mixture was refluxed for 12 h, subsequently cooled, and filtered through Celite 545 Merck (Darmstadt, Germany). The organic layer was washed with water and next with brine, dried over anhydrous Na_2SO_4, and finally concentrated in vacuo. The Boc protected amines **A1**–**A4** were purified by column chromatography using the following developing system S_1.

***Tert*-butyl 4-(3,5-dichlorophenyl)piperazine-1-carboxylate (A1).** Yellow oil, yield 65% (2.15 g); TLC: R_f = 0.83 (S_1); UPLC (purity 98.1%): t_R = 9.32 min. LC-MS (ESI): *m/z* calcd for $C_{15}H_{20}Cl_2N_2O_2$ $(M+H)^+$ 331.09, found 331.1.

***Tert*-butyl 4-(3-(trifluoromethoxy)phenyl)piperazine-1-carboxylate (A2)**. Yellow oil, yield 68% (2.36 g); TLC: R_f = 0.72 (S_1); UPLC (purity 98.4%): t_R = 8.73 min. LC-MS (ESI): *m/z* calcd for $C_{16}H_{21}F_3N_2O_3$ $(M+H)^+$ 347.16, found 347.2.

***Tert*-butyl 4-(3-phenoxyphenyl)piperazine-1-carboxylate (A3).** Yellow oil, yield 67% (2.37 g); TLC: R_f = 0.77 (S_1); UPLC (purity 92.3%): t_R = 9.07 min. LC-MS (ESI): *m/z* calcd for $C_{21}H_{26}N_2O_3$ $(M+H)^+$ 355.20, found 355.3.

***Tert*-butyl 4-(3-((trifluoromethyl)thio)phenyl)piperazine-1-carboxylate (A4).** Yellow oil, yield 71% (2.57 g); TLC: R_f = 0.74 (S_1); UPLC (purity 93.8%): t_R = 9.14 min. LC-MS (ESI): *m/z* calcd for $C_{16}H_{21}F_3N_2O_2S$ $(M+H)^+$ 363.13, found 363.2.

General method for the preparation of starting amines A5–A8. The DCM (5 mL) solution of **A1–A4** (5 mmol, 1 eq) was treated with TFA (1.71 g, 15 mmol, 3 eq) and stirred at room temperature for 3 h. Afterwards, the organic solvents were evaporated to dryness. The resulting oil residue was dissolved in water (20 mL), and then 25% ammonium hydroxide was carefully added to pH = 8. The aqueous layer was extracted with DCM (3 × 20 mL), dried over Na_2SO_4, and concentrated to give the **A5–A8** as yellow or bronze oils. Starting amines **A5–A8** were advanced as substrates for the next reactions without additional purification.

1-(3,5-Dichlorophenyl)piperazine (A5) Yellow oil, yield 97% (1.21 g); TLC: R_f = 0.48 (S_2); UPLC (purity 98.3%): t_R = 3.99 min. LC-MS (ESI): *m/z* calcd for $C_{10}H_{12}Cl_2N_2$ $(M+H)^+$ 231.04, found 231.0. ^{1}H NMR (500 MHz, $CDCl_3$) δ 2.02–2.18 (m, 1H, piperazine), 2.97–3.00 (m, 4H, piperazine), 3.11–3.14 (m, 4H, piperazine), 6.72 (s, 2H, ArH), 6.77 (s, 1H, ArH).

1-(3-(Trifluoromethoxy)phenyl)piperazine (A6). Yellow oil, yield 97% (1.20 g); TLC: R_f = 0.41 (S_2); UPLC (purity 95.7%): t_R = 3.99 min. LC-MS (ESI): *m/z* calcd for $C_{11}H_{13}F_3N_2O$ $(M+H)^+$ 247.10, found 247.1. ^{1}H NMR (300 MHz, $CDCl_3$) δ 1.23 (s, 1H, piperazine), 2.91–3.16 (m, 8H, piperazine), 6.59–6.85 (m, 3H, ArH), 7.19 (t, *J*=8.3 Hz, 1 H. ArH).

1-(3-Phenoxyphenyl)piperazine (A7) Yellow oil, yield 97% (1.23 g); TLC: R_f = 0.45 (S_2); UPLC (purity 93.6%): t_R = 4.61 min. LC-MS (ESI): *m/z* calcd for $C_{16}H_{18}N_2O$ $(M+H)^+$ 255.15, found 255.3. ^{1}H NMR (500 MHz, $CDCl_3$) δ 2.59–2.69 (m, 1 H, piperazine), 3.00 (s, 1 H, piperazine), 3.14–3.22 (m, 4 H, piperazine), 3.27–3.34 (m, 3 H, piperazine), 6.41–6.72 (m, 3 H, ArH), 6.96–7.39 (m, 6 H, ArH).

1-(3-((Trifluoromethyl)thio)phenyl)piperazine (A8) Yellow oil, yield 96% (1.25 g); TLC: R_f = 0.42 (S_2); UPLC (purity 93.9%): t_R = 3.69 min. LC-MS (ESI): *m/z* calcd for $C_{11}H_{13}N_2SF_3$ $(M+H)^+$ 263.08, found 263.1. ^{1}H NMR (500 MHz, $CDCl_3$) δ 2.65–2.71 (m, 1H, piperazine), 3.10–3.16 (m, 3H, piperazine), 3.18–3.31 (m, 5H, piperazine), 6.99–7.01 (m, 1H, ArH), 7.09–7.16 (m, 2H, ArH), 7.25–7.30 (m, 1H, ArH).

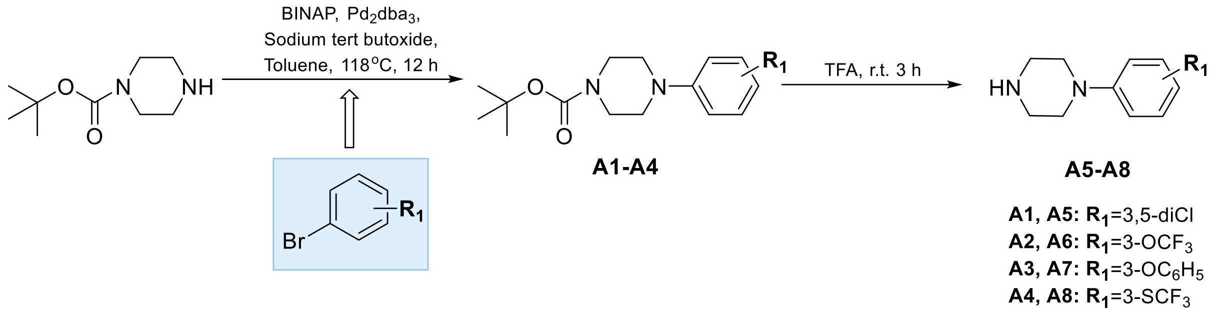

Scheme S1. Synthesis of Boc-protected intermediates (**A1**–**A4**) and non-commercial 4-arylpiperazine derivatives **A5**–**A8**.

The procedure for synthesis, physicochemical and spectra data for A9-A16

General method for the preparation of intermediates A9–A12. The starting (non-commercial) Boc-derivatives of 1-phenylpyrrolidin-3-amine were obtained by the *N*-arylation reaction. Appropriate aryl bromide (10 mmol, 1 eq), Pd_2dba_3 (0.37 g, 0.4 mmol, 0.04 eq), BINAP (0.37 g, 0.6 mmol, 0.06 eq), caesium carbonate (3.26 g, 10 mmol, 1 eq), and pyrrolidin-3-amine (3.74 g, 20 mmol, 2 eq) were suspended under an inert gas (nitrogen) atmosphere in 50 mL of dry toluene. The reaction mixture was refluxed for 12 h, subsequently cooled, and filtered through Celite 545 Merck (Darmstadt, Germany). The organic layer was washed with water and next with brine, dried over anhydrous $Na2SO4$, and finally concentrated in vacuo. The Boc protected intermediates **A9**–**A12** were purified by column chromatography using the following developing system S_1.

***Tert*-butyl (1-phenylpyrrolidin-3-yl)carbamate (A9)** Yellow oil, yield 66% (1.73 g); TLC: R_f = 0.76 (S_1); UPLC (purity 96.6%): t_R = 7.38 min. LC-MS (ESI): *m/z* calcd for $C_{15}H_{22}N_2O_2$ $(M+H)^+$ 263.17, found 263.2.

***Tert*-butyl (1-(3-(trifluoromethyl)phenyl)pyrrolidin-3-yl)carbamate (A10)** Yellow oil, yield 62% (2.05 g); TLC: R_f = 0.81 (S_1); UPLC (purity 95.3%): t_R = 8.20 min. LC-MS (ESI): *m/z* calcd for $C_{16}H_{21}F_3N_2O_2$ $(M+H)^+$ 331.16, found 331.3.

***Tert*-butyl (1-(3-(trifluoromethoxy)phenyl)pyrrolidin-3-yl)carbamate (A11)** Yellow oil, yield 68% (2.35 g); TLC: R_f = 0.83 (S_1); UPLC (purity 97.8%): t_R = 8.39 min. LC-MS (ESI): *m/z* calcd for $C_{16}H_{21}F_3N_2O_3$ $(M+H)^+$ 347.15, found 347.3.

***Tert*-butyl (1-(3-((trifluoromethyl)thio)phenyl)pyrrolidin-3-yl)carbamate (A12)** Yellow oil, yield 63% (2.28 g); TLC: R_f = 0.84 (S_1); UPLC (purity 95.1%): t_R = 8.78 min. LC-MS (ESI): *m/z* calcd for $C_{16}H_{21}F_3N_2O_2S$ $(M+H)^+$ 363.13, found 363.2.

General method for the preparation of starting amines A13–A16. The DCM (5 mL) solution of **A9–A12** (5 mmol, 1 eq) was treated with TFA (1.71 g, 15 mmol, 3 eq) and stirred at room temperature for 3 h. Afterwards, the organic solvents were evaporated to dryness. The resulting oil residue was dissolved in water (20 mL), and then 25% ammonium hydroxide was carefully added to pH = 8. The aqueous layer was extracted with DCM (3 × 20 mL), dried over Na_2SO_4, and concentrated to give the **A13–A16** as yellow or bronze oils. Starting amines **A13–A16** were advanced as substrates for the next reactions without additional purification.

1-phenylpyrrolidin-3-amine (A13) Yellow oil, yield 97% (0.81 g); TLC: R_f = 0.40 (S_2); UPLC (purity 98.3%): t_R = 2.73 min. LC-MS (ESI): *m/z* calcd for $C_{10}H_{14}N_2$ $(M+H)^+$ 163.12, found 163.3. ^1H NMR (500 MHz, $CDCl_3$) δ 1.46–1.93 (m, 3 H, pyrrolidin-3-amine), 2.14–2.33 (m, 1 H, pyrrolidin-3-amine), 3.01 (dd, *J*=9.3, 4.7 Hz, 1 H, pyrrolidin-3-amine), 3.22–3.80 (m, 4 H, pyrrolidin-3-amine), 6.43–6.60 (m, 2 H, ArH), 6.64–6.74 (m, 1 H, ArH), 7.07–7.37 (m, 2 H, ArH).

1-(3-(trifluoromethyl)phenyl)pyrrolidin-3-amine (A14) Yellow oil, yield 98% (1.11 g); TLC: R_f = 0.44 (S_2); UPLC (purity 96.8%): t_R = 4.06 min. LC-MS (ESI): *m/z* calcd for $C_{11}H_{13}F_3N_2$ $(M+H)^+$ 231.11, found 231.2. ^1H NMR (500 MHz, $CDCl_3$) δ 1.63–2.05 (m, 3 H, pyrrolidin-3-amine), 2.13–2.38 (m, 1 H, pyrrolidin-3-amine), 3.04 (dd, *J*=9.5, 4.6 Hz, 1 H, pyrrolidin-3-amine), 3.25–3.40 (m, 1 H, pyrrolidin-3-amine), 3.43–3.59 (m, 2 H, pyrrolidin-3-amine), 3.67–3.81 (m, 1 H, pyrrolidin-3-amine), 6.51–6.80 (m, 2 H, ArH), 6.84–6.95 (m, 1 H, ArH), 7.16–7.38 (m, 1 H, ArH).

1-(3-(trifluoromethoxy)phenyl)pyrrolidin-3-amine (A15) Bronze oil, yield 96% (1.18 g); TLC: R_f = 0.46 (S_2); UPLC (purity 97.7%): t_R = 4.27 min. LC-MS (ESI): *m/z* calcd for $C_{11}H_{13}F_3N_2O$ $(M+H)^+$ 247.10, found 247.1. ^1H NMR (500 MHz, $CDCl_3$) δ 1.70–2.39 (m, 4 H, pyrrolidin-3-amine), 2.88–3.12 (m, 1 H, pyrrolidin-3-amine), 3.19–3.81 (m, 4 H, pyrrolidin-3-amine), 6.20–6.64 (m, 3 H, ArH), 6.93–7.22 (m, 1 H, ArH).

1-(3-((trifluoromethyl)thio)phenyl)pyrrolidin-3-amine (A16) Bronze oil, yield 97% (1.18 g); TLC: R_f = 0.47 (S_2); UPLC (purity 96.2%): t_R = 4.63 min. LC-MS (ESI): *m/z* calcd for $C_{11}H_{13}F_3N_2S$ $(M+H)^+$ 263.08, found 263.1. ^1H NMR (500 MHz, $CDCl_3$) δ 1.49–2.03 (m, 3 H, pyrrolidin-3-amine), 2.07–2.42 (m, 1 H, pyrrolidin-3-amine), 2.93–3.80 (m, 5 H, pyrrolidin-3-amine), 6.61 (dd, *J*=8.3, 2.3 Hz, 1 H, ArH), 6.76 (s, 1 H, ArH), 6.91 (d, *J*=7.5 Hz, 1 H, ArH), 7.14–7.33 (m, 1 H, ArH).

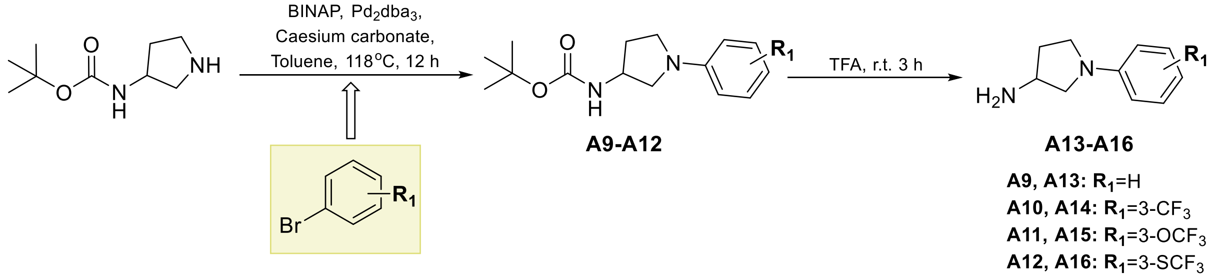

Scheme S2. Synthesis of Boc-protected intermediates (**A9**-**A12**) and 1-phenylpyrrolidin-3-amine derivatives **A13**–**A16**.

Table S1. Antiseizure activity screening data for compounds from **Series 1**, **2** and **3** in mice *i.p.* (100 mg/kg).

Cmpd	R_1	R_2	MES[a]	6 Hz (32 mA)[b]	*sc*PTZ[c]
**21**	H	-	0/4	1/4	-
**22**	3-Cl	-	4/4	3/4	1/4
**23**	4-Cl	-	0/4	0/4	-
**24**	3,4-diCl	-	3/4	4/4	3/4
**25**	3,5-diCl	-	3/4	3/4	2/4
**26**	3-CF_3	-	4/4	4/4	1/4
**27**	4-CF_3	-	1/4	0/4	-
**28**	3-OCF_3	-	4/4	4/4	1/4
**29**	3-OC_6H_5	-	4/4	3/4	0/4
**30**	3-SCF_3	-	4/4	4/4	2/4
**31**	3-Cl	C_6H_5	0/4	0/4	-
**32**	3-CF_3	C_6H_5	0/4	1/4	-
**33**	3-Cl	NHC_2H_5	0/4	2/4	-
**34**	3-CF_3	NHC_2H_5	0/4	2/4	-
**35**	3-Cl	NHC_3H_7	0/4	2/4	-
**36**	3-CF_3	NHC_3H_7	1/4	1/4	-
**45**	H	-	0/4	0/4	-
**46**	3-CF_3	-	0/4	0/4	-
**47**	3-OCF_3	-	0/4	2/4	-
**48**	3-SCF_3	-	0/4	2/4	-

Data indicate number of mice protected/number of mice tested. Dose of 100 mg/kg was administered *i.p.* The animals were examined at 0.5 h.. A dash indicates not tested. [a] MES – maximal electroshock seizure test; [b] 6 Hz – psychomotor seizure test, 32 mA; [c] *sc*PTZ – subcutaneous pentylenetetrazole seizure test. A dash indicate not tested.

Table S2. Radioligand binding and functional assays.

**Binding studies**	
Na^+ channel (site 2)	[1]
NMDA (antagonist radioligand)	[2]
AMPA (agonist radioligand)	[3]
$Ca_v2.2$ calcium ion channel (antagonist radioligand)	[4]
GABA transporter (antagonist radioligand)	[5]
$GABA_A$ ion channel [^{3}H]GABA (agonist radioligand)	[6]
GABA transaminase	[7]
Potassium channel (hERG)	[8]
**Functional studies**	
TRPV1 (VR1) (h) (antagonist effect)	[9]
$Cav_{1.2}$ (L-type) (h) calcium ion channel cell-based antagonist calcium flux assay	[10,11]
5-HT_{2C} (agonist effect)	[12]

Assays were performed commercially in Eurofins Laboratories (Poitiers, France) or Eurofins Panlabs Discovery Services Taiwan, Ltd. (New Taipei City, Taiwan).

Table S3. The most probable metabolic pathways of **26** and **28** determined *in silico* and *in vitro*.

Substrate	Molecular mass (m/z)	Molecular mass of the metabolite (m/z)	Metabolic pathway
**26**	344.27	356.24 (M1	*dehydrogenation and oxidation*
		356.51 (M2)	*dehydrogenation and oxidation*
**28**	360.23	356.51 (M1)	*double dehydrogenation*
		376.24 (M2)	*hydroxylation*
		356.24 (M3)	*double dehydrogenation*
**Verapamil***	455.31	441.35 (M1)	*demethylation*
		291.328 (M2)	*defragmentation*
		165.09 (M3)	*defragmentation*
		441.29 (M4)	*demethylation*
		427.33 (M5)	*double-demethylation*
		277.26 (M6)	*defragmentation*

* Reference unstable drug; metabolic stability determined using the same procedure published previously [13]

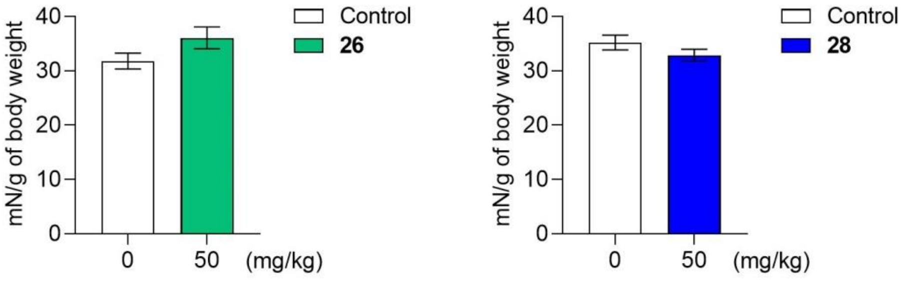

Figure S1. Acute effects of compound **26** (**A**) and **28** (**B**) on grip strength in mice. Both compounds were administered *i.p.* at a dose of 50 mg/kg, 30 min before the test. Control animals received vehicle. Data are presented as means ± SEM of grip strength expressed in mN/g of body weight (n = 10animals). The statistical significance was evaluated by a Student's t test (GraphPad Prism 8).

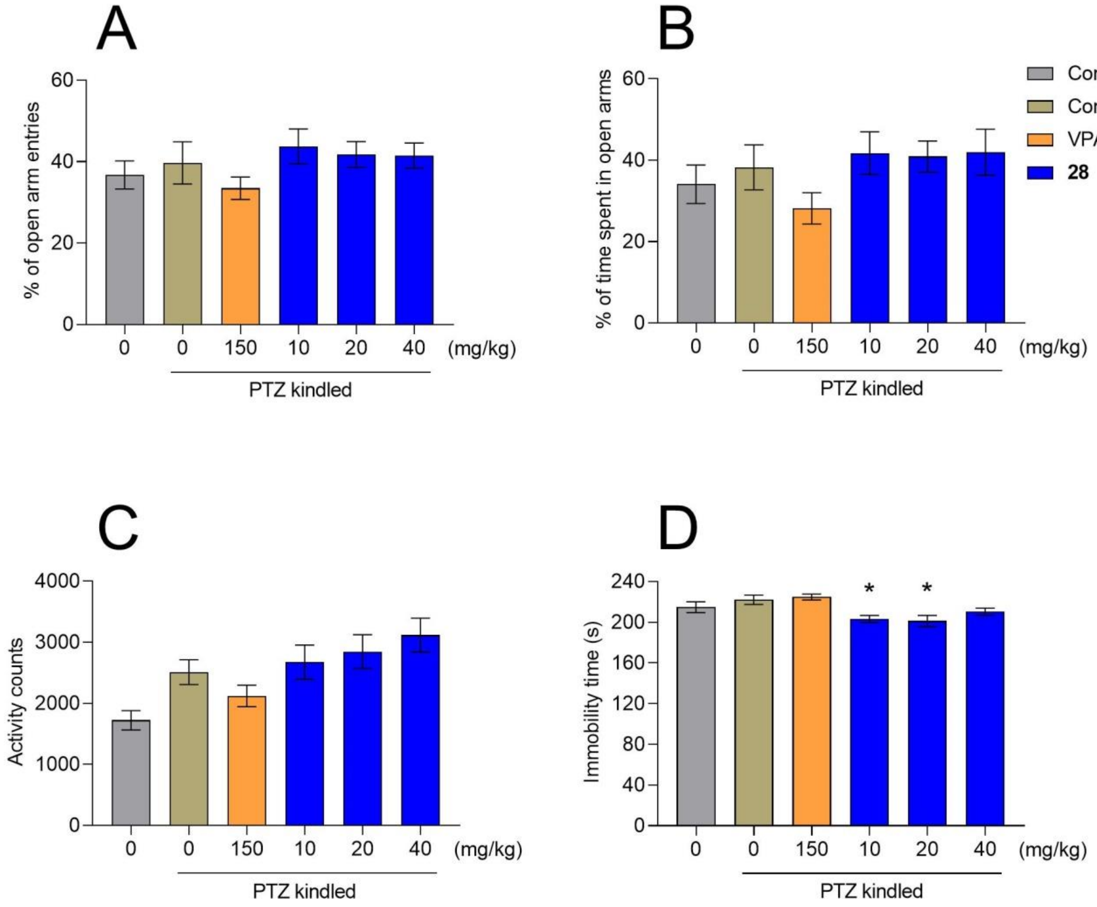

Figure S2. Behavioral tests used to evaluate the effect of compound **28** on: (**A**) the percentage of the open arms entries in the elevated plus maze test; (**B**) the percentage of the time spent in the open arms in the elevated plus maze test; (**C**) spontaneous locomotor activity; (**D**) total immobility duration in the forced swim test in mice subjected to the PTZ kindling procedure. PTZ (40 mg/kg, *i.p.*) was given three times a week, 30 min after administration of compound **28**, VPA, or vehicle. Behavioral tests were performed 24 h after the last PTZ injection. Data are shown as means ± SEM (n = 10–15 animals). The statistical significance was evaluated by one-way ANOVA followed by the Tukey’s post hoc test: *p < 0.05, (GraphPad Prism 8).

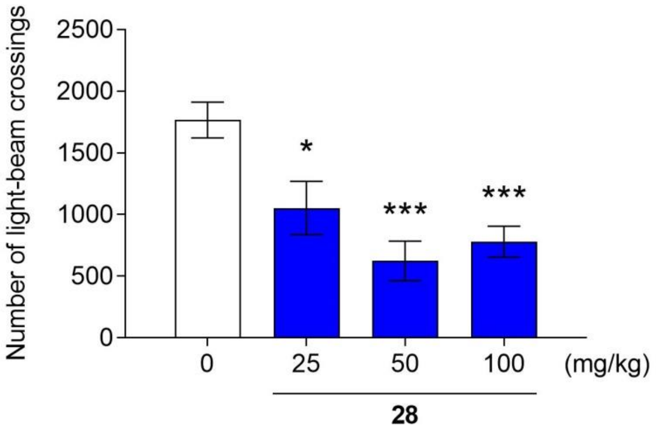

Figure S3. The effect of compound **28** on the spontaneous locomotor activity in mice. The test compound or vehicle (1% Tween 80 solution) were administered 30 min *i.p.* before the 30-min long evaluation of locomotor activity. The results are presented as bar plots showing the mean ± SEM. The statistical significance was evaluated by one-way ANOVA followed by Dunnett's post hoc test: * $p < 0.05$, *** $p < 0.001$, n = 8–10 mice per group.

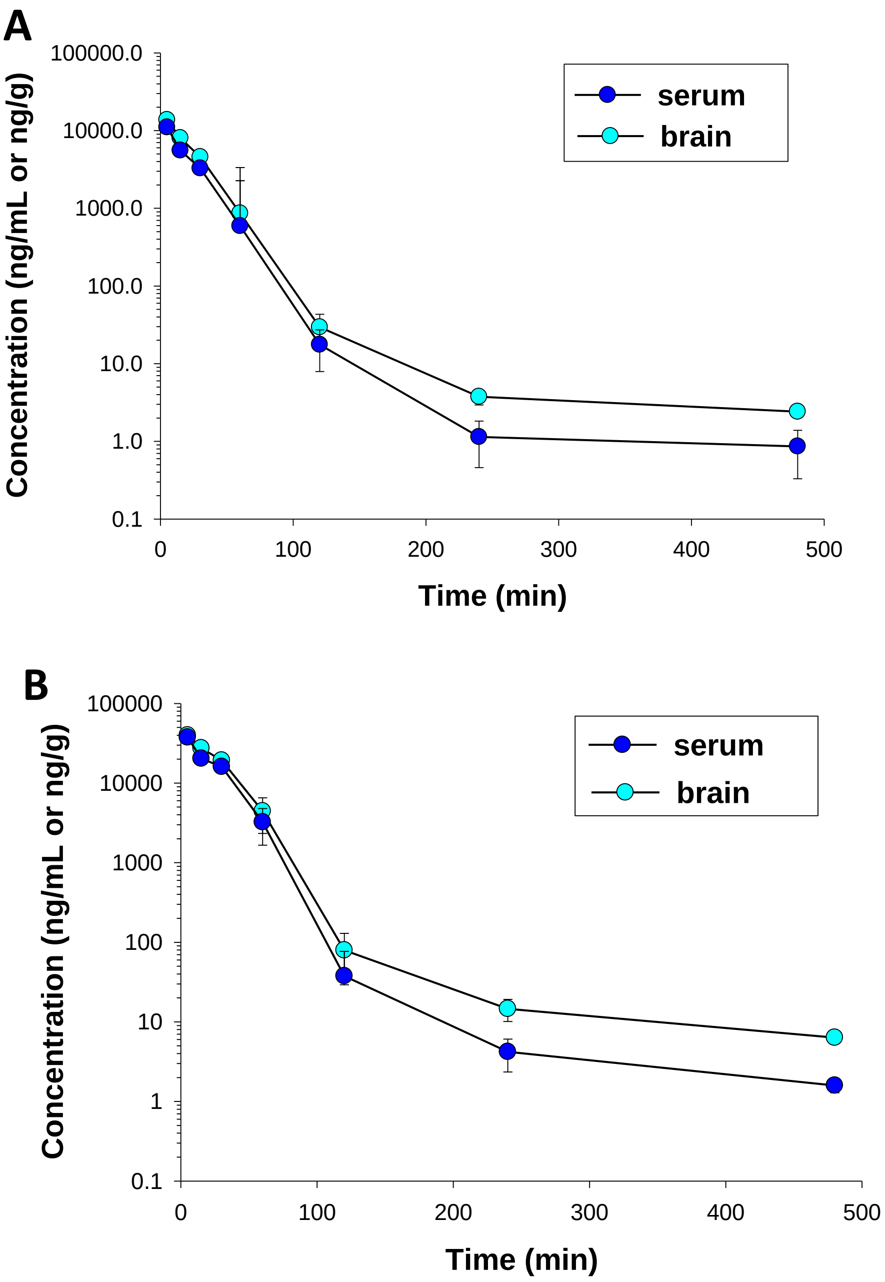

Figure S4. Mean (±SD) serum and brain concentrations of **28** after *i.p.* administration of this compound at two doses 25 mg/kg (A) and 50 mg/kg (B) to mice (n=3-4).

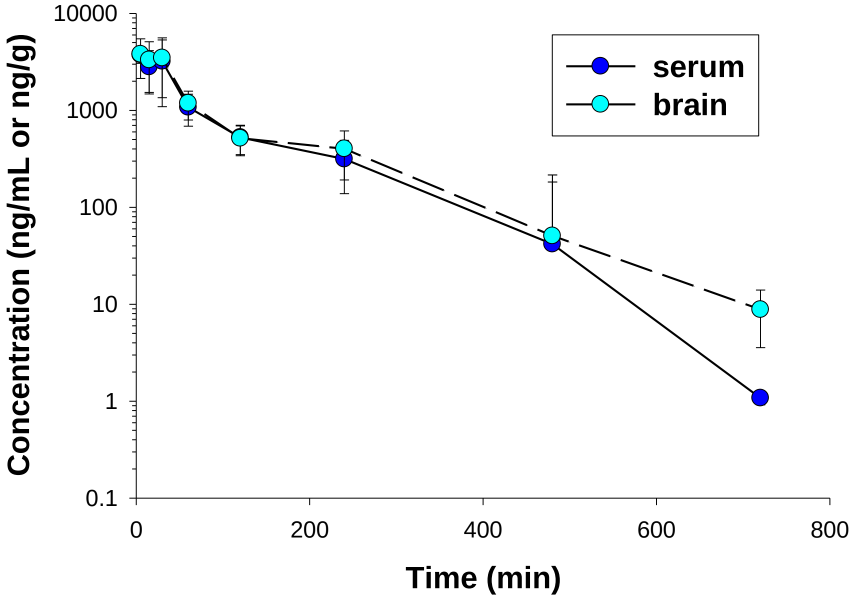

Figure S5. Mean (±SD) serum and brain concentrations of **28** after *p.o.* administration at a dose of 25 mf/kg to mice (n=3-4).

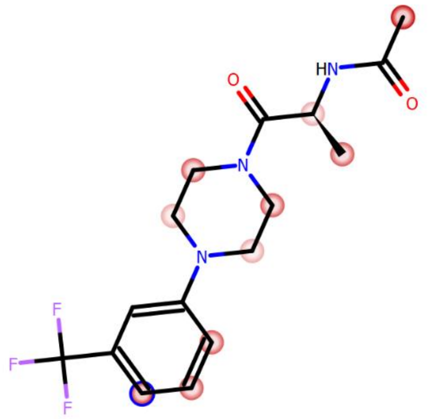

Figure S6. MetaSite prediction of the most probable site of **26** metabolism.

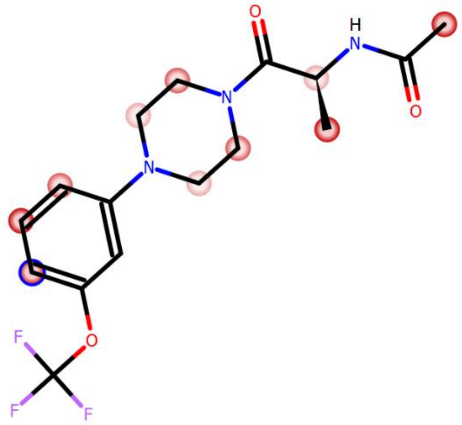

Figure S7. MetaSite prediction of the most probable site of **28** metabolism

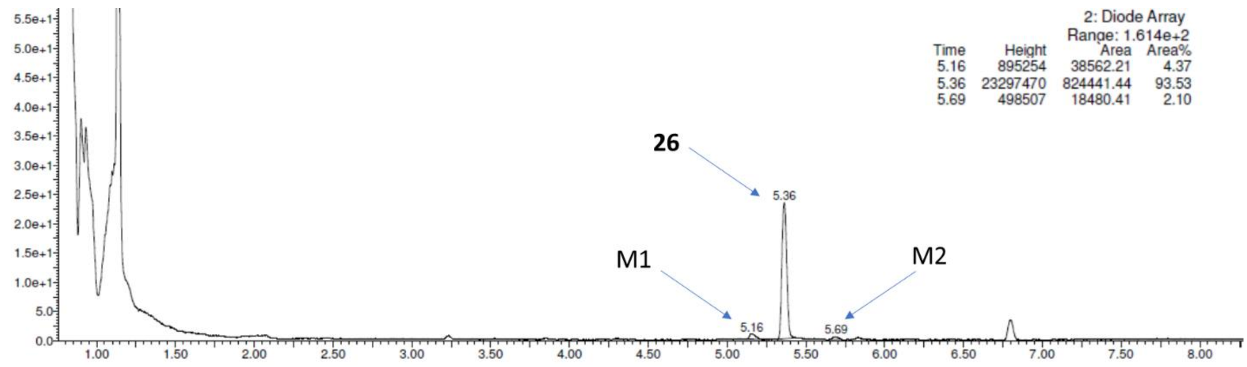

Figure S8. UPLC of the reaction mixture after incubation of **26** with HLMs for 120 min.

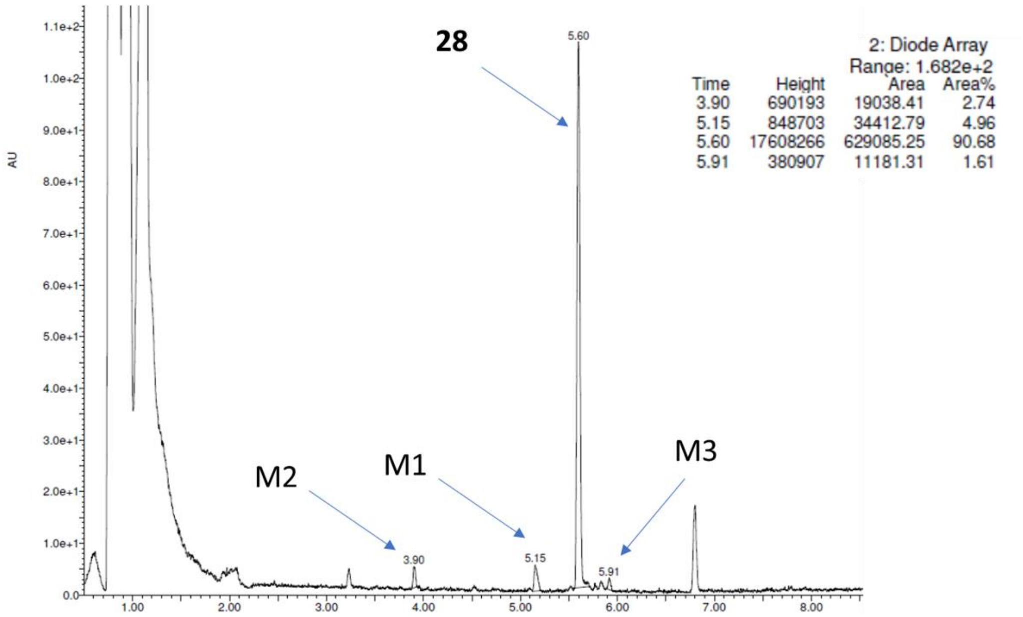

Figure S9. UPLC of the reaction mixture after incubation of **28** with HLMs for 120 min.

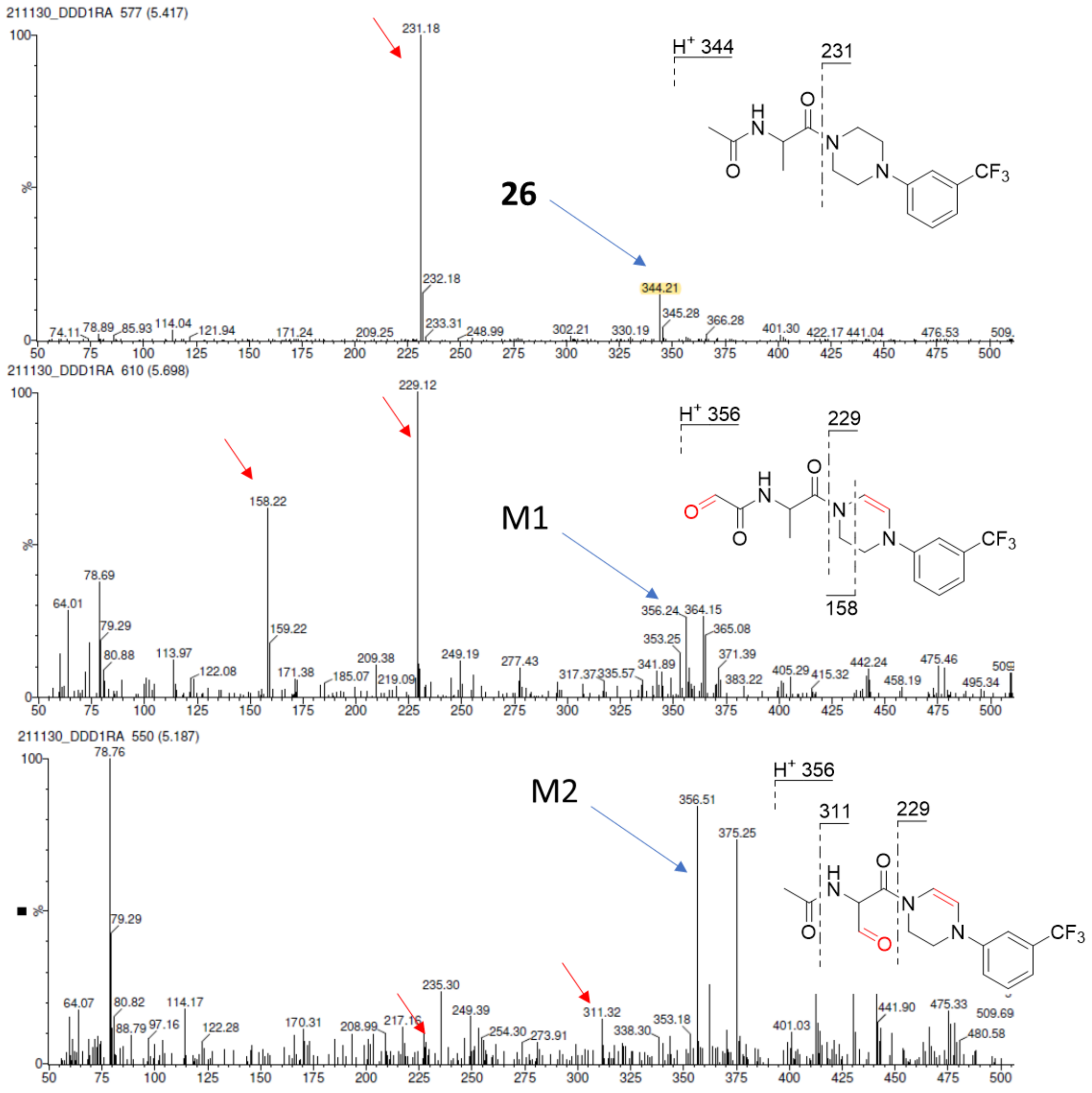

Figure S10. MS spectra of compound **26** and its metabolites.

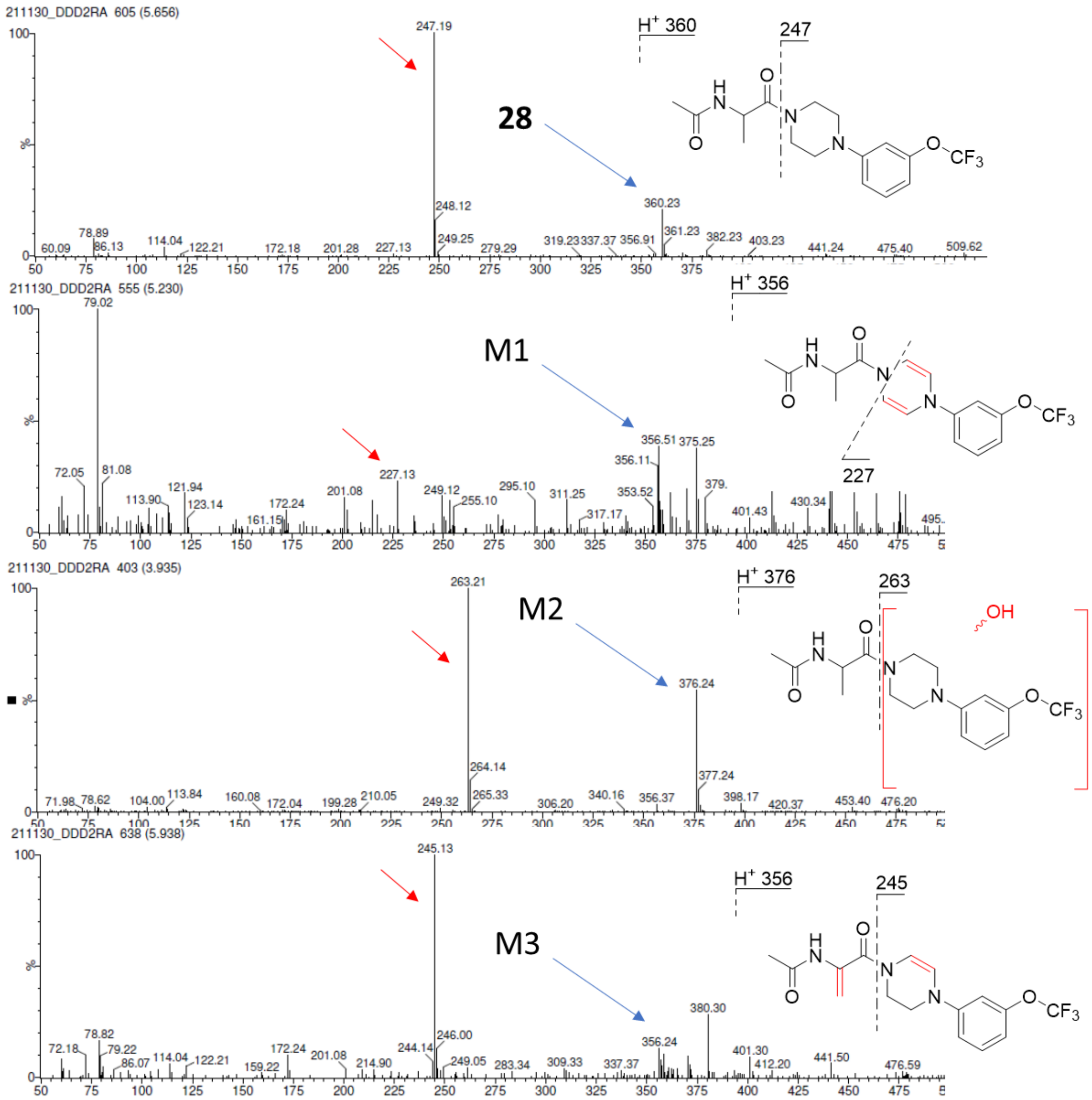

Figure S11. MS spectra of compound **28** and its metabolites.

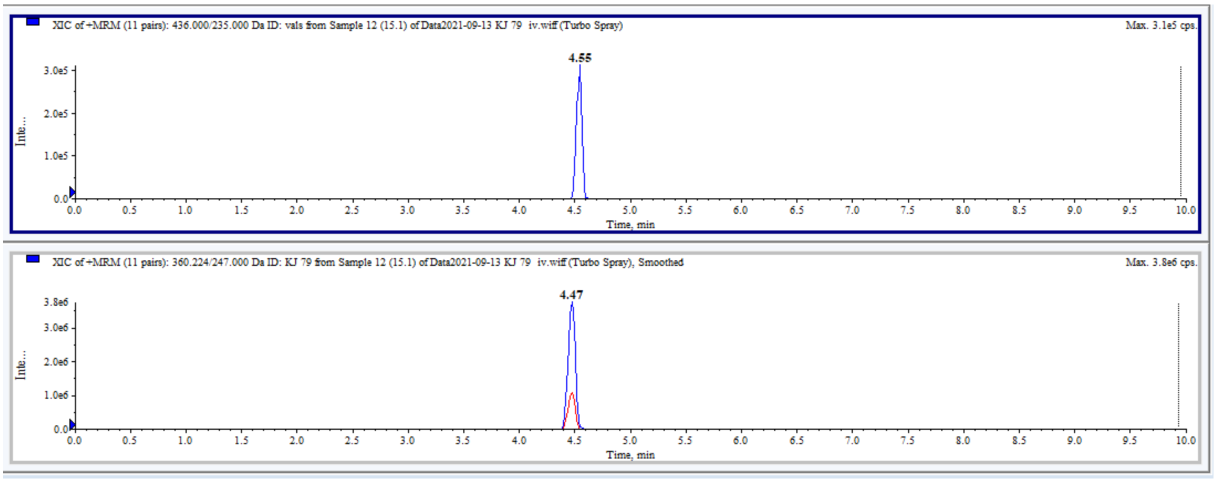

Figure S12. Extracted ions chromatogram of compound **28** (m/z 360/247 and 360/204) and internal standard (m/z 436/207). Serum sample collected 15 min after *i.v.* administration of the analyzed compound at the dose of 10 mg/kg.

References:

1. Brown, G.B. 3H-Batrachotoxinin-A Benzoate Binding to Voltage-Sensitive Sodium Channels: Inhibition by the Channel Blockers Tetrodotoxin and Saxitoxin. *J. Neurosci.* **1986**, *6*, 2064–2070, doi:10.1523/JNEUROSCI.06-07-02064.1986.
2. Sills, M.A.; Fagg, G.; Pozza, M.; Angst, C.; Brundish, D.E.; Hurt, S.D.; Jay Wilusz, E.; Williams, M. [3H]CGP 39653: A New N-Methyl-D-Aspartate Antagonist Radioligand with Low Nanomolar Affinity in Rat Brain. *Eur. J. Pharmacol.* **1991**, *192*, 19–24, doi:10.1016/0014-2999(91)90063-V.
3. Murphy, D.E.; Snowhill, E.W.; Williams, M. Characterization of Quisqualate Recognition Sites in Rat Brain Tissue usingDl-[3H]α-Amino-3-Hydroxy-5-Methylisoxazole-4-Propionic Acid (AMPA) and a Filtration Assay. *Neurochem. Res.* **1987**, *12*, 775–781, doi:10.1007/BF00971514.
4. Wagner, J.A.; Snowman, A.M.; Biswas, A.; Olivera, B.M.; Snyder, S.H. Omega-Conotoxin GVIA Binding to a High-Affinity Receptor in Brain: Characterization, Calcium Sensitivity, and Solubilization. *J. Neurosci.* **1988**, *8*, 3354–3359, doi:10.1523/JNEUROSCI.08-09-03354.1988.
5. Shank, R.P.; Baldy, W.J.; Mattucci, L.C.; Villani Jr., F.J. Ion and Temperature Effects on the Binding of γ-Aminobutyrate to Its Receptors and the High-Affinity Transport System. *J. Neurochem.* **1990**, *54*, 2007–2015, doi:10.1111/j.1471-4159.1990.tb04905.x.
6. McLeod, M.; Pralong, D.; Copolov, D.; Dean, B. The Heterogeneity of Central Benzodiazepine Receptor Subtypes in the Human Hippocampal Formation, Frontal Cortex and Cerebellum Using [3H]Flumazenil and Zolpidem. *Mol. Brain Res.* **2002**, *104*, 203–209, doi:10.1016/S0169-328X(02)00381-9.
7. Löscher, W. Effect of Inhibitors of GABA Aminotransferase on the Metabolism of GABA in Brain Tissue and Synaptosomal Fractions. *J. Neurochem.* **1981**, *36*, 1521–1527, doi:10.1111/j.1471-4159.1981.tb00595.x.
8. Huang, X.-P.; Mangano, T.; Hufeisen, S.; Setola, V.; Roth, B.L. Identification of Human Ether-à-Go-Go Related Gene Modulators by Three Screening Platforms in an Academic Drug-Discovery Setting. *ASSAY Drug Dev. Technol.* **2010**, *8*, 727–742, doi:10.1089/adt.2010.0331.
9. Phelps, P.T.; Anthes, J.C.; Correll, C.C. Cloning and Functional Characterization of Dog Transient Receptor Potential Vanilloid Receptor-1 (TRPV1). *Eur. J. Pharmacol.* **2005**, *513*, 57–66, doi:10.1016/j.ejphar.2005.02.045.
10. Sirenko, O.; Crittenden, C.; Callamaras, N.; Hesley, J.; Chen, Y.-W.; Funes, C.; Rusyn, I.; Anson, B.; Cromwell, E.F. Multiparameter In Vitro Assessment of Compound Effects on Cardiomyocyte Physiology Using iPSC Cells. *J Biomol Screen* **2013**, *18*, 39–53, doi:10.1177/1087057112457590.
11. Xia, M.; Imredy, J.P.; Koblan, K.S.; Bennett, P.; Connolly, T.M. State-Dependent Inhibition of L-Type Calcium Channels: Cell-Based Assay in High-Throughput Format. *Anal. Biochem.* **2004**, *327*, 74–81, doi:10.1016/j.ab.2004.01.003.
12. Porter, R.H.P.; Benwell, K.R.; Lamb, H.; Malcolm, C.S.; Allen, N.H.; Revell, D.F.; Adams, D.R.; Sheardown, M.J. Functional Characterization of Agonists at Recombinant Human 5-HT2A, 5-HT2B and 5-HT2C Receptors in CHO-K1 Cells. *Br. J. Pharmacol.* **1999**, *128*, 13–20, doi:10.1038/sj.bjp.0702751.
13. Więckowska, A.; Wichur, T.; Godyń, J.; Bucki, A.; Marcinkowska, M.; Siwek, A.; Więckowski, K.; Zaręba, P.; Knez, D.; Głuch-Lutwin, M.; et al. Novel Multitarget-Directed Ligands Aiming at Symptoms and Causes of Alzheimer's Disease. *ACS Chem. Neurosci.* **2018**, *9*, 1195–1214, doi:10.1021/acschemneuro.8b00024.

HMRS traces for selected final compounds

N-(1-(4-(3-chlorophenyl)piperazin-1-yl)-1-oxopropan-2-yl)acetamide (22)

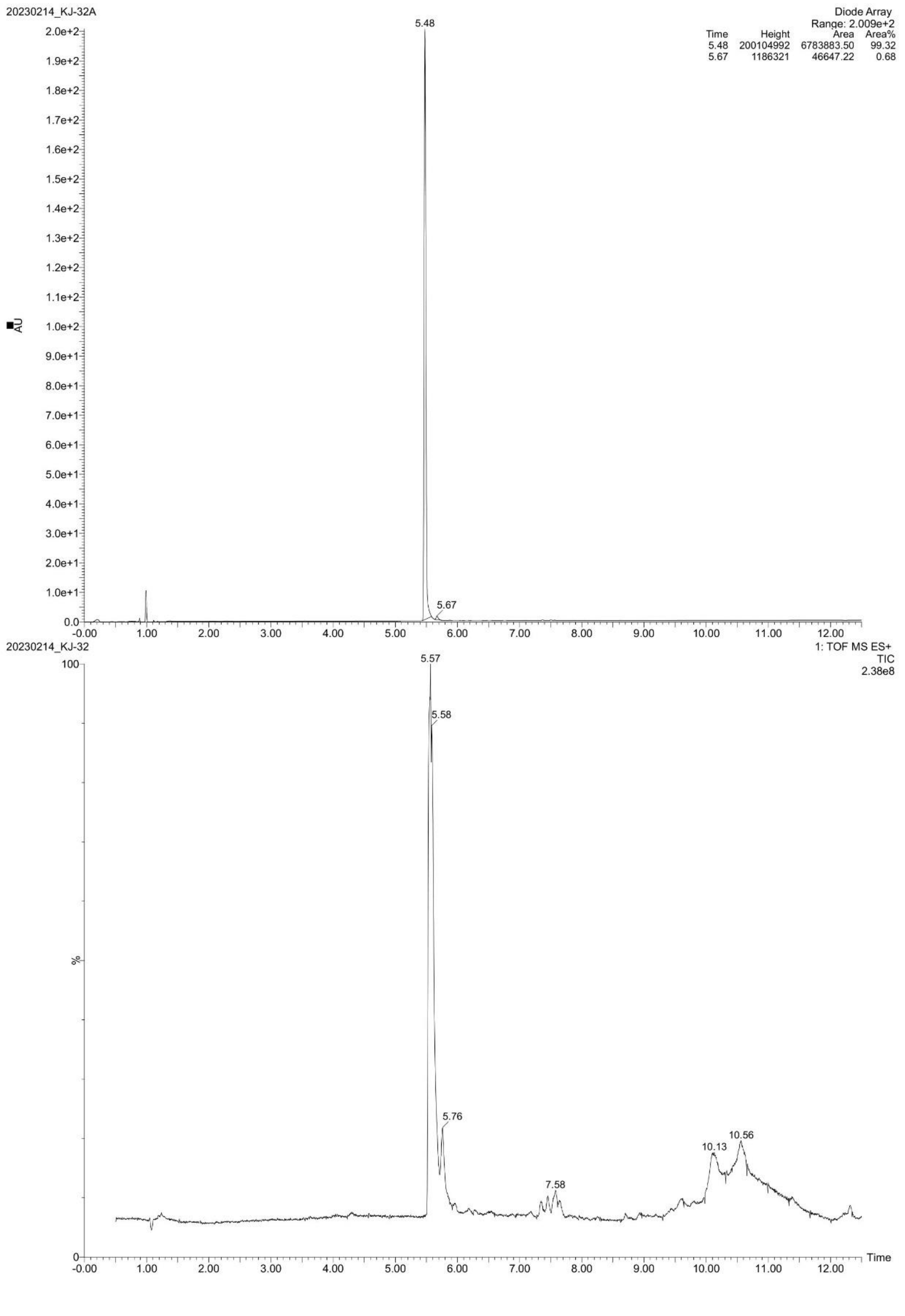

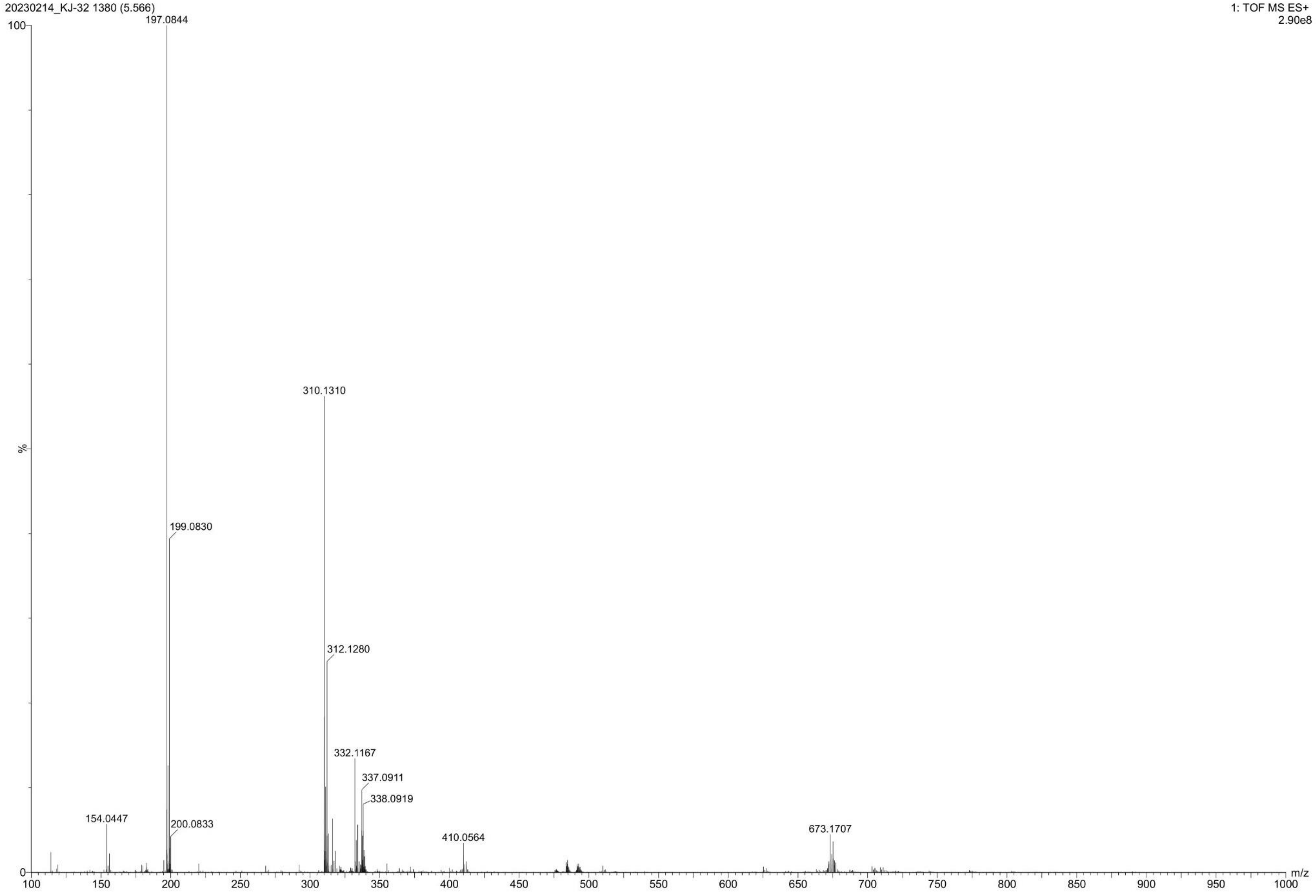
20230214_KJ-32 1380 (5.566)
1: TOF MS ES+
2.90e8
100
%
0
197.0844
310.1310
199.0830
312.1280
332.1167
337.0911
338.0919
154.0447
200.0833
410.0564
673.1707
m/z
100
150
200
250
300
350
400
450
500
550
600
650
700
750
800
850
900
950
1000

***N*-(1-(4-(3,4-dichlorophenyl)piperazin-1-yl)-1-oxopropan-2-yl)acetamide (24)**

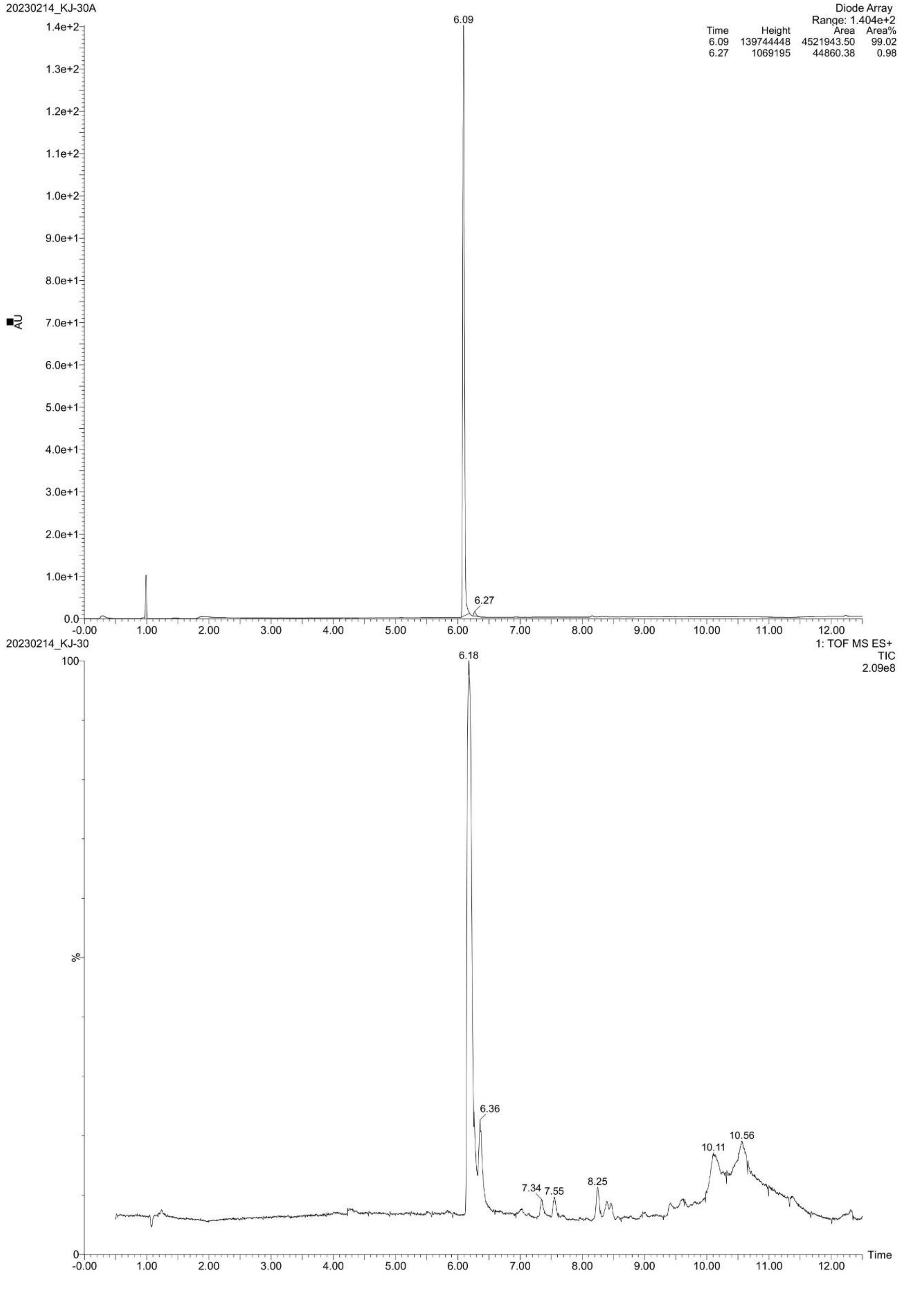

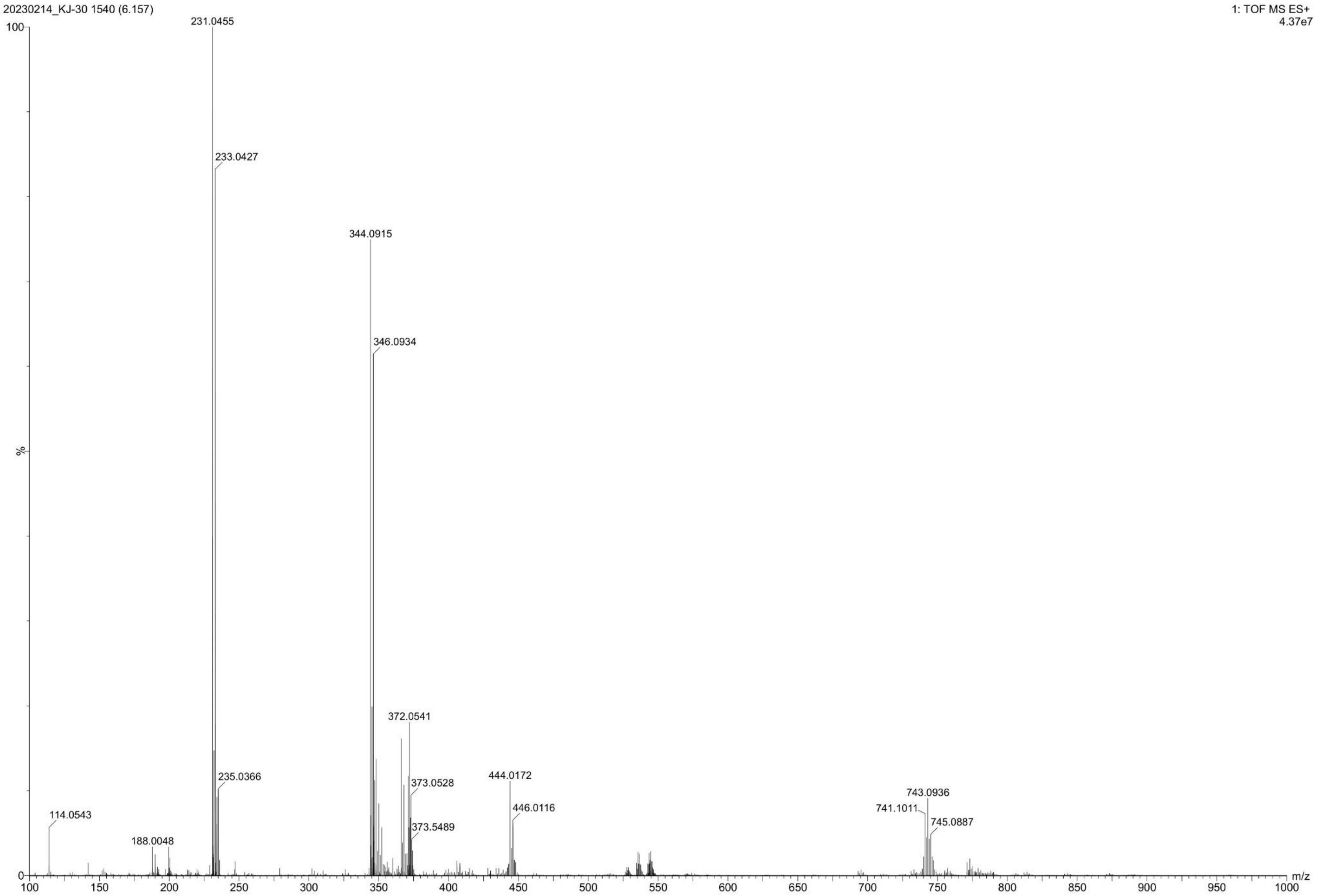
20230214_KJ-30 1540 (6.157)
1: TOF MS ES+
4.37e7
231.0455
233.0427
344.0915
346.0934
372.0541
373.0528
373.5489
235.0366
444.0172
446.0116
114.0543
188.0048
743.0936
741.1011
745.0887
%
100
0
m/z
100
150
200
250
300
350
400
450
500
550
600
650
700
750
800
850
900
950
1000

***N*-(1-(4-(3,5-dichlorophenyl)piperazin-1-yl)-1-oxopropan-2-yl)acetamide (25)**

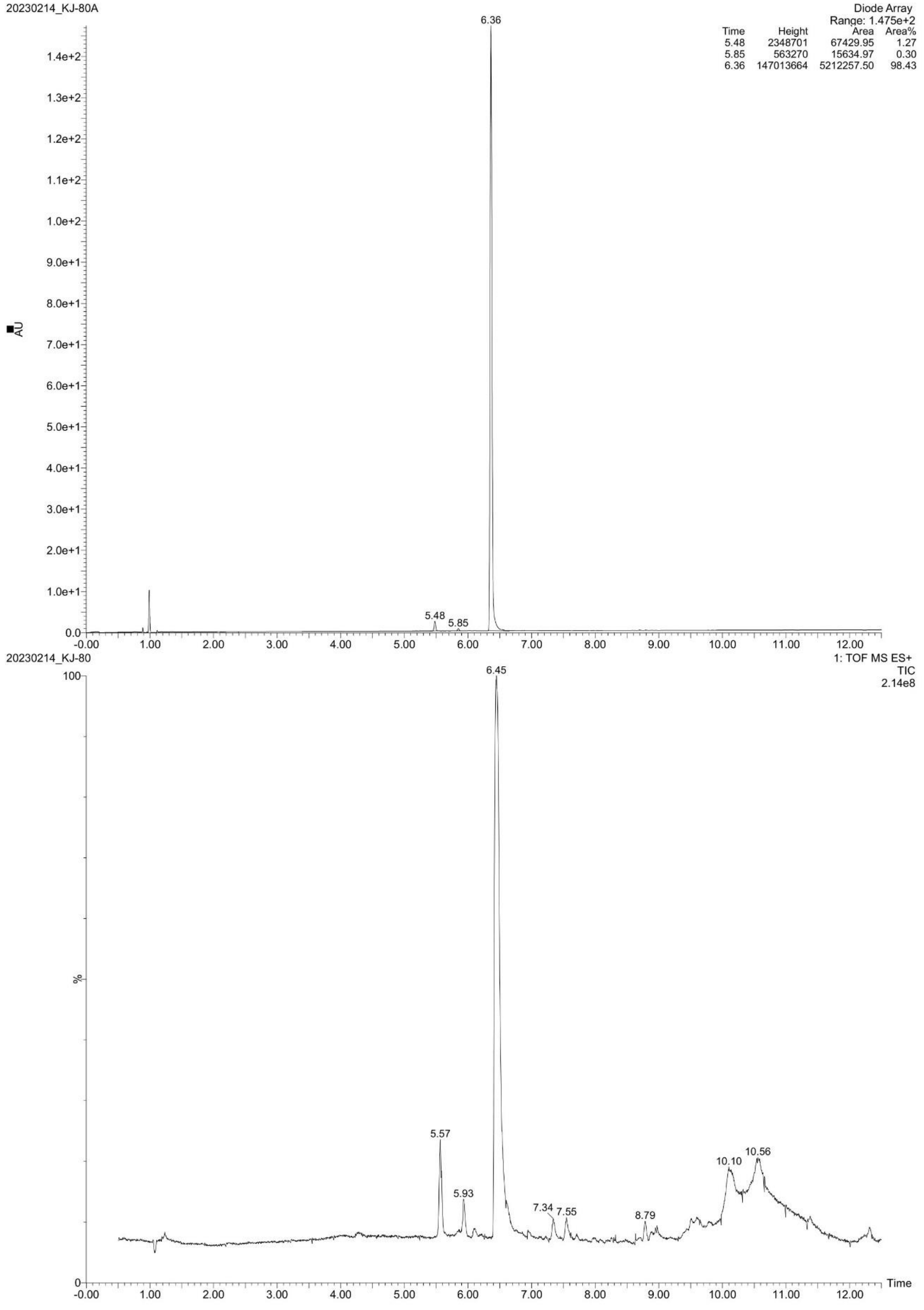

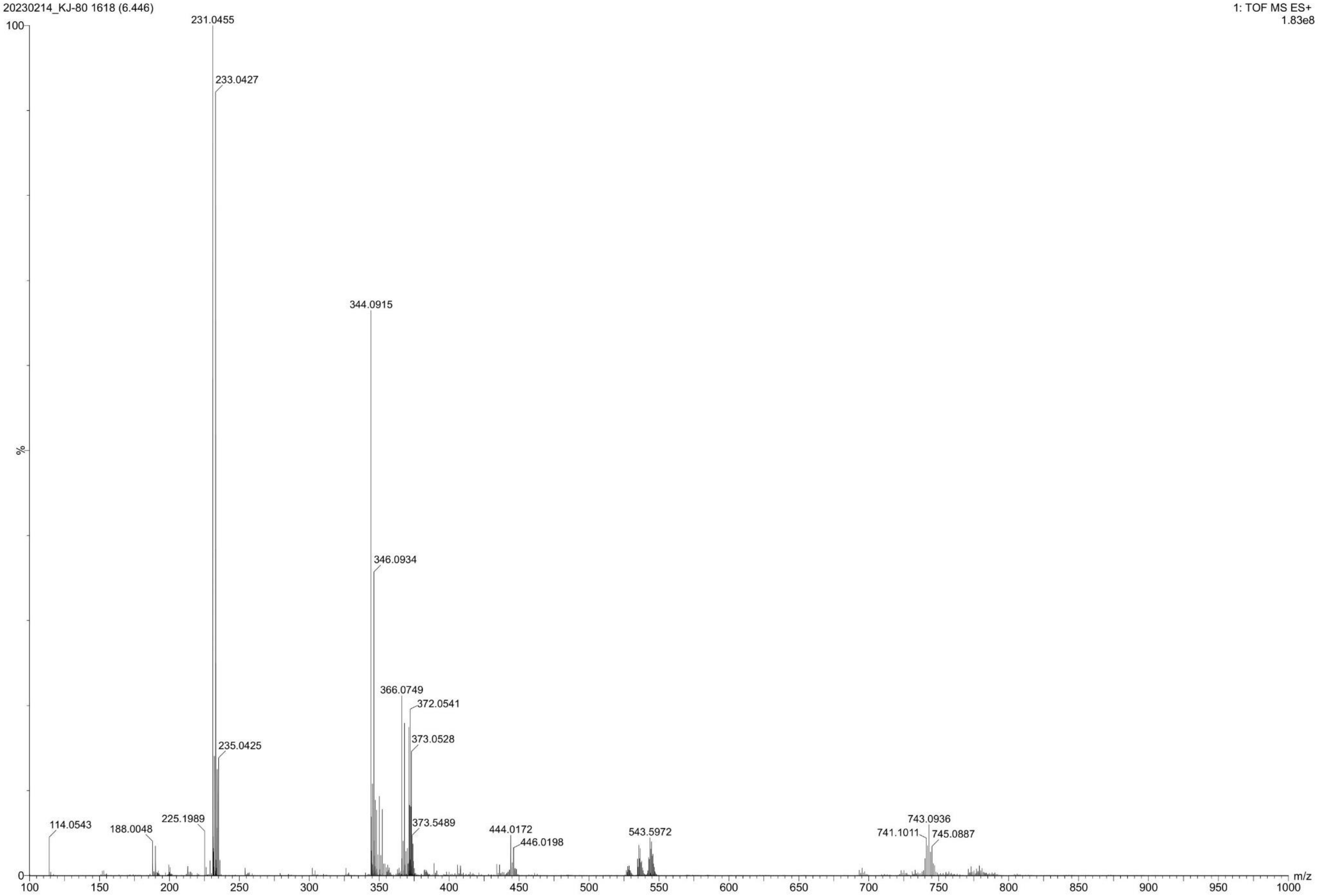

20230214_KJ-80 1618 (6.446)
1: TOF MS ES+
1.83e8
100
%
0
231.0455
233.0427
344.0915
346.0934
366.0749
372.0541
373.0528
235.0425
114.0543
188.0048
225.1989
373.5489
444.0172
446.0198
543.5972
743.0936
741.1011
745.0887
100
150
200
250
300
350
400
450
500
550
600
650
700
750
800
850
900
950
1000
m/z

***N*-(1-oxo-1-(4-(3-(trifluoromethyl)phenyl)piperazin-1-yl)propan-2-yl)acetamide (26)**

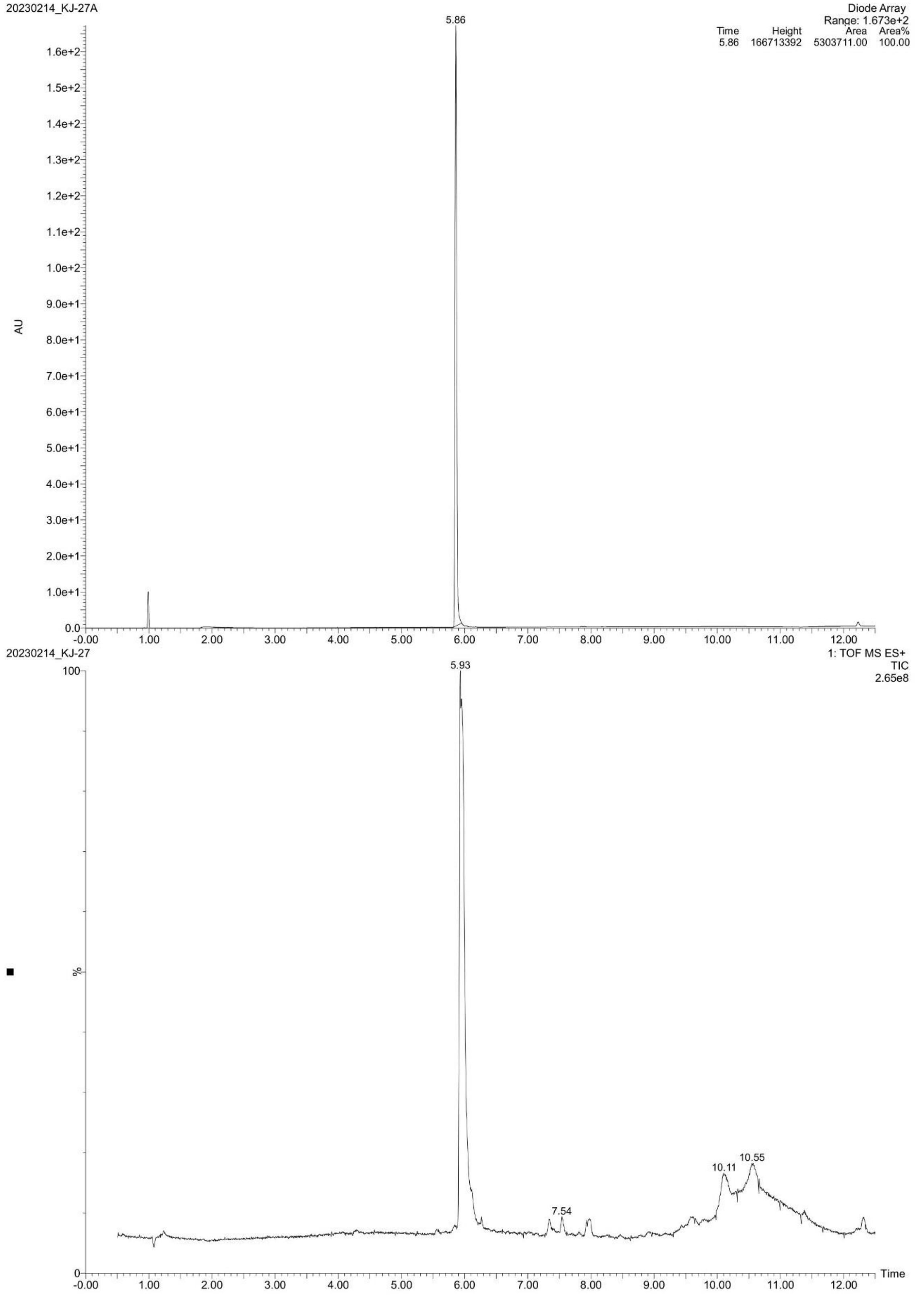

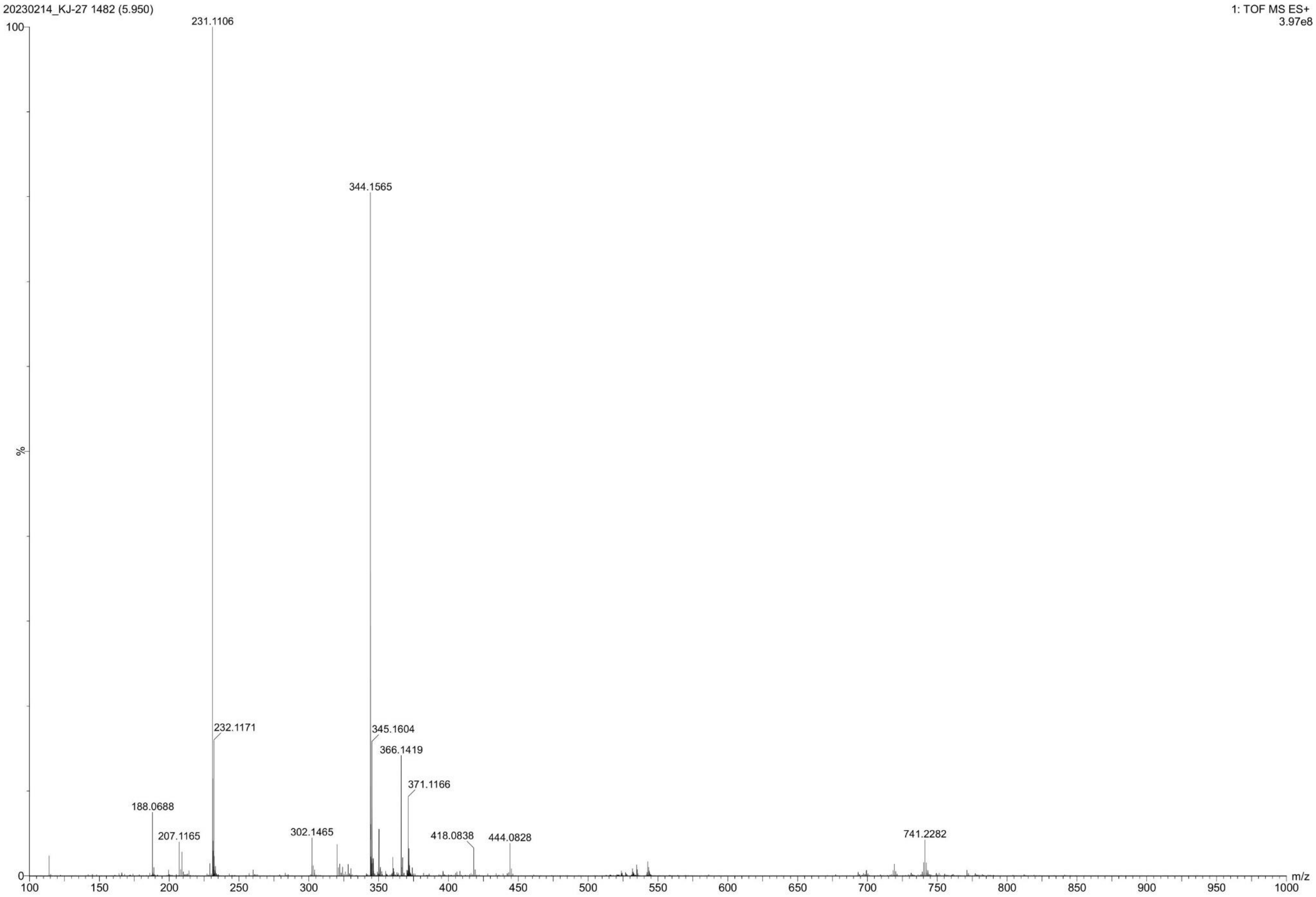
20230214_KJ-27 1482 (5.950)
1: TOF MS ES+
3.97e8
100
%
0
231.1106
344.1565
232.1171
345.1604
366.1419
371.1166
188.0688
207.1165
302.1465
418.0838
444.0828
741.2282
100
150
200
250
300
350
400
450
500
550
600
650
700
750
800
850
900
950
1000
m/z

***N*-(1-oxo-1-(4-(3-(trifluoromethoxy)phenyl)piperazin-1-yl)propan-2-yl)acetamide (28)**

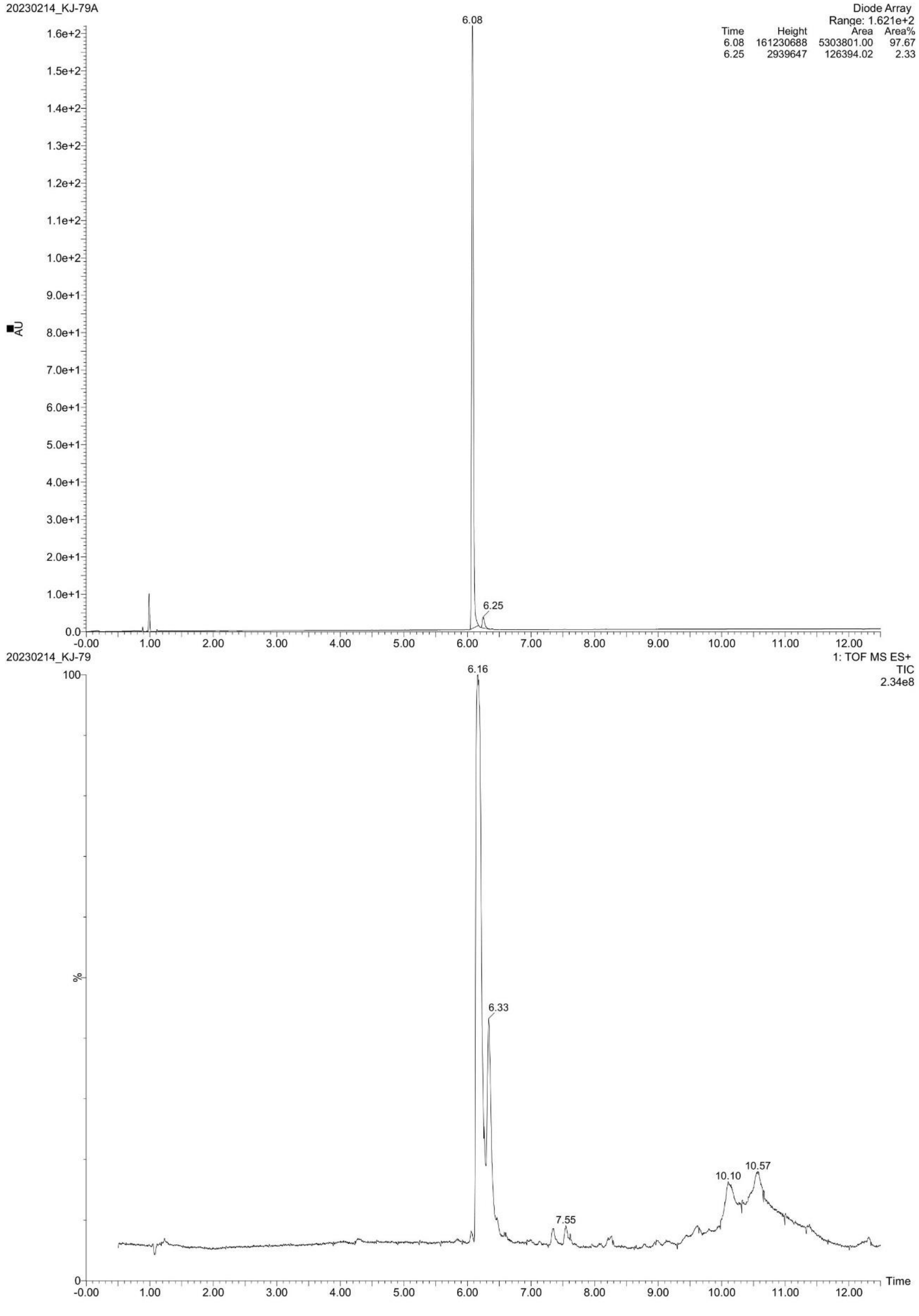

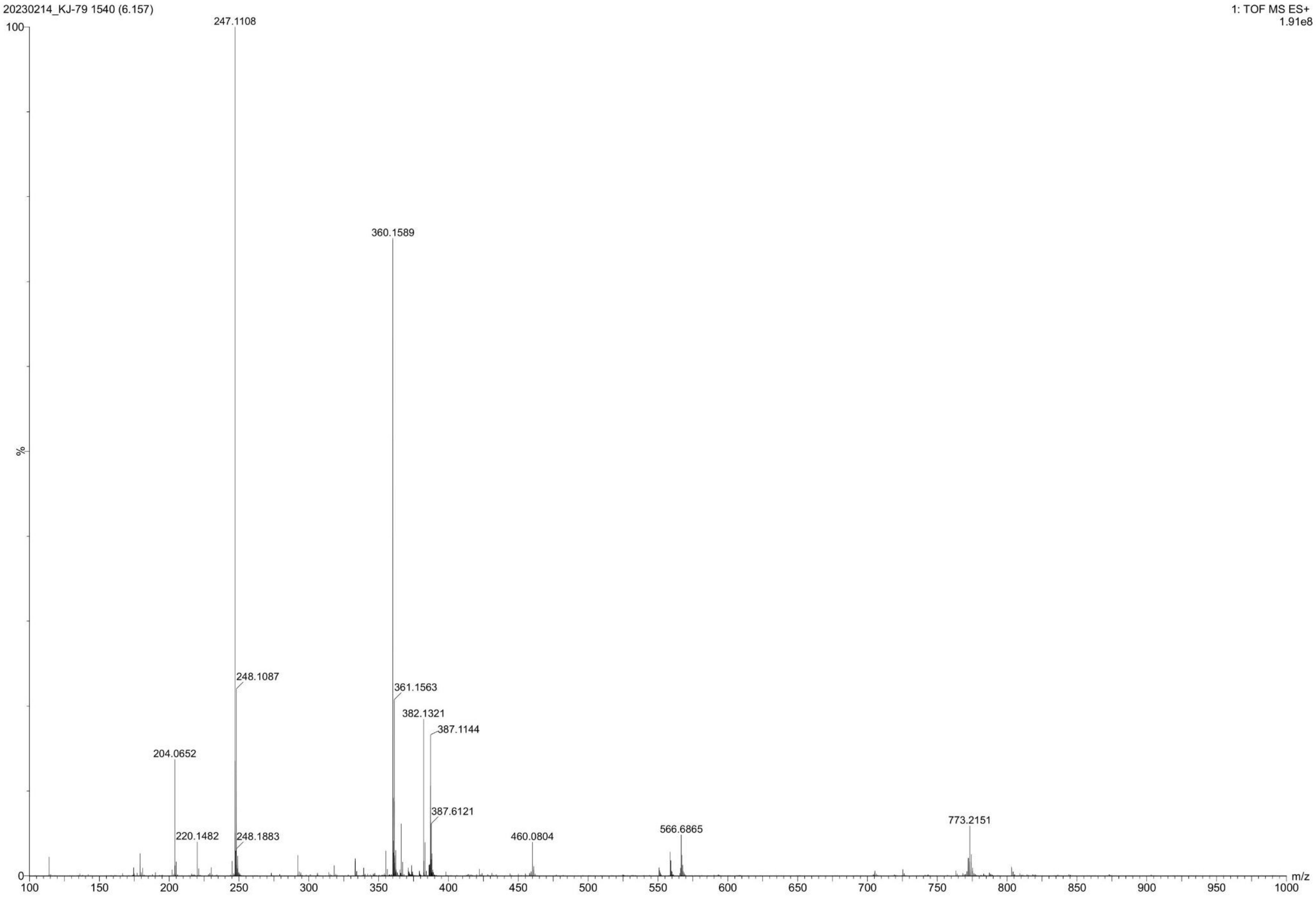
20230214_KJ-79 1540 (6.157)
1: TOF MS ES+
1.91e8
100
%
0
247.1108
360.1589
248.1087
361.1563
382.1321
387.1144
204.0652
387.6121
220.1482
248.1883
460.0804
566.6865
773.2151
m/z
100
150
200
250
300
350
400
450
500
550
600
650
700
750
800
850
900
950
1000

***N*-(1-oxo-1-(4-(3-phenoxyphenyl)piperazin-1-yl)propan-2-yl)acetamide (29)**

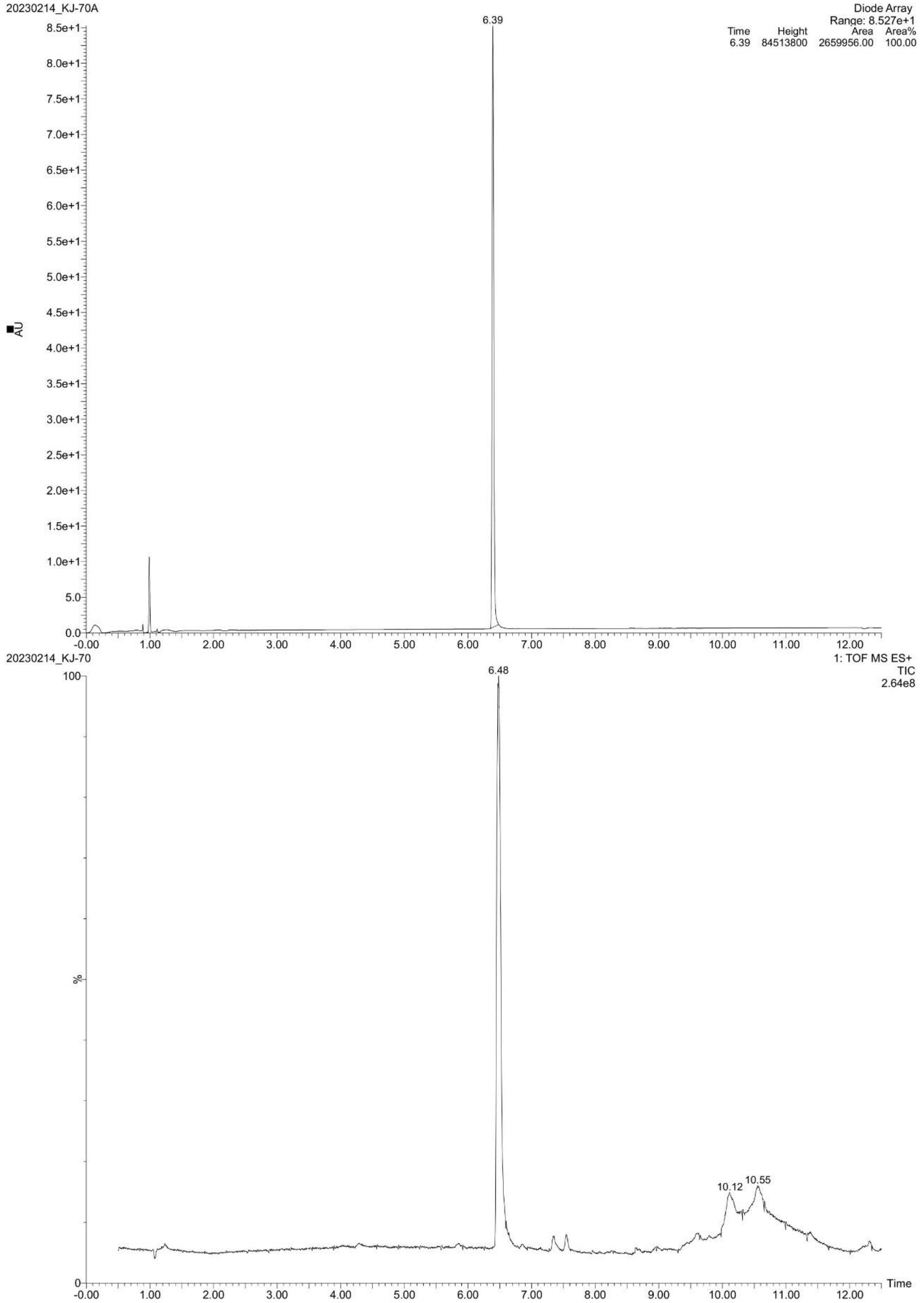

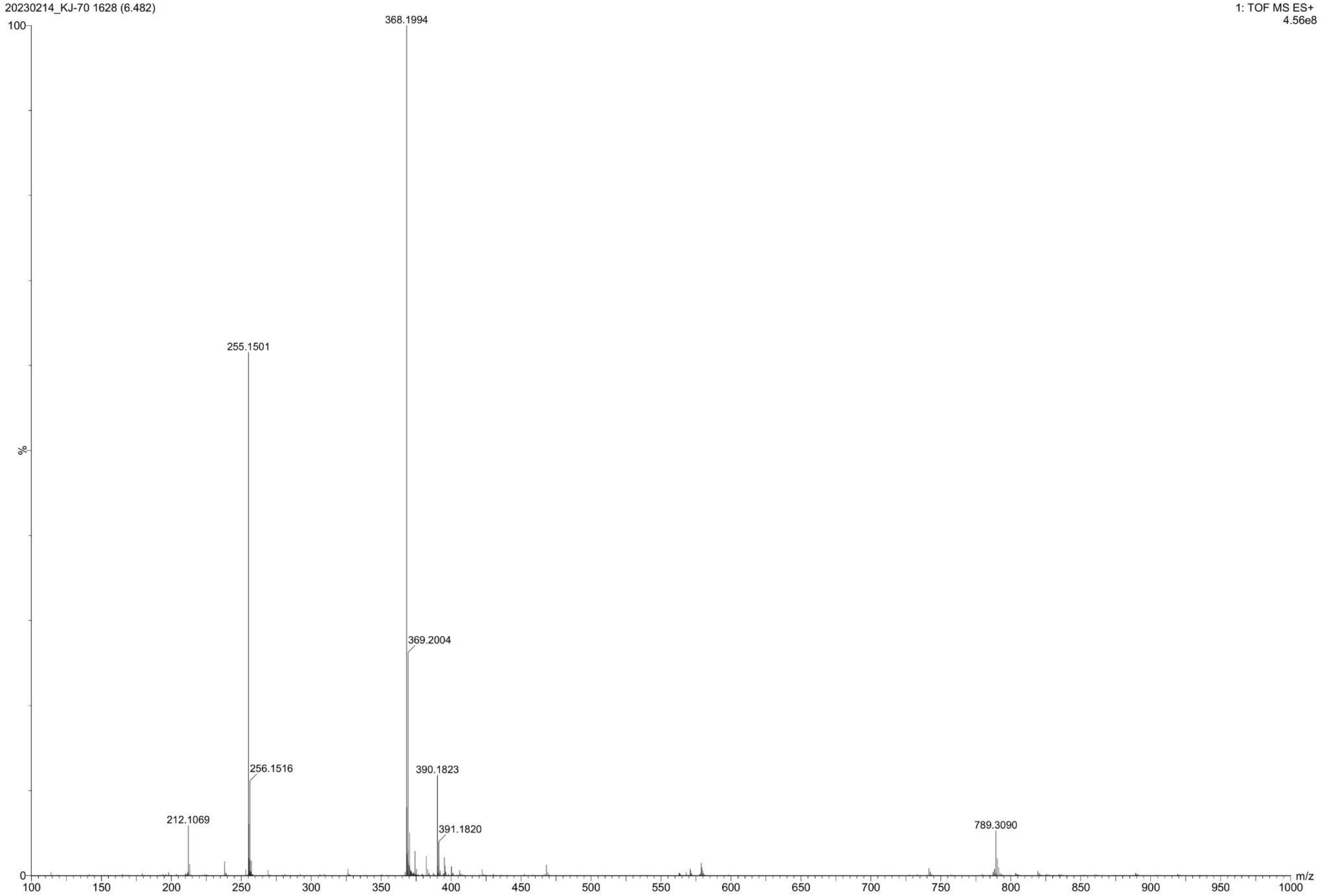
20230214_KJ-70 1628 (6.482)
1: TOF MS ES+
4.56e8
100
%
0
368.1994
255.1501
369.2004
256.1516
390.1823
212.1069
391.1820
789.3090
m/z
100
150
200
250
300
350
400
450
500
550
600
650
700
750
800
850
900
950
1000

N-(1-oxo-1-(4-(3-((trifluoromethyl)thio)phenyl)piperazin-1-yl)propan-2-yl)acetamide (30)

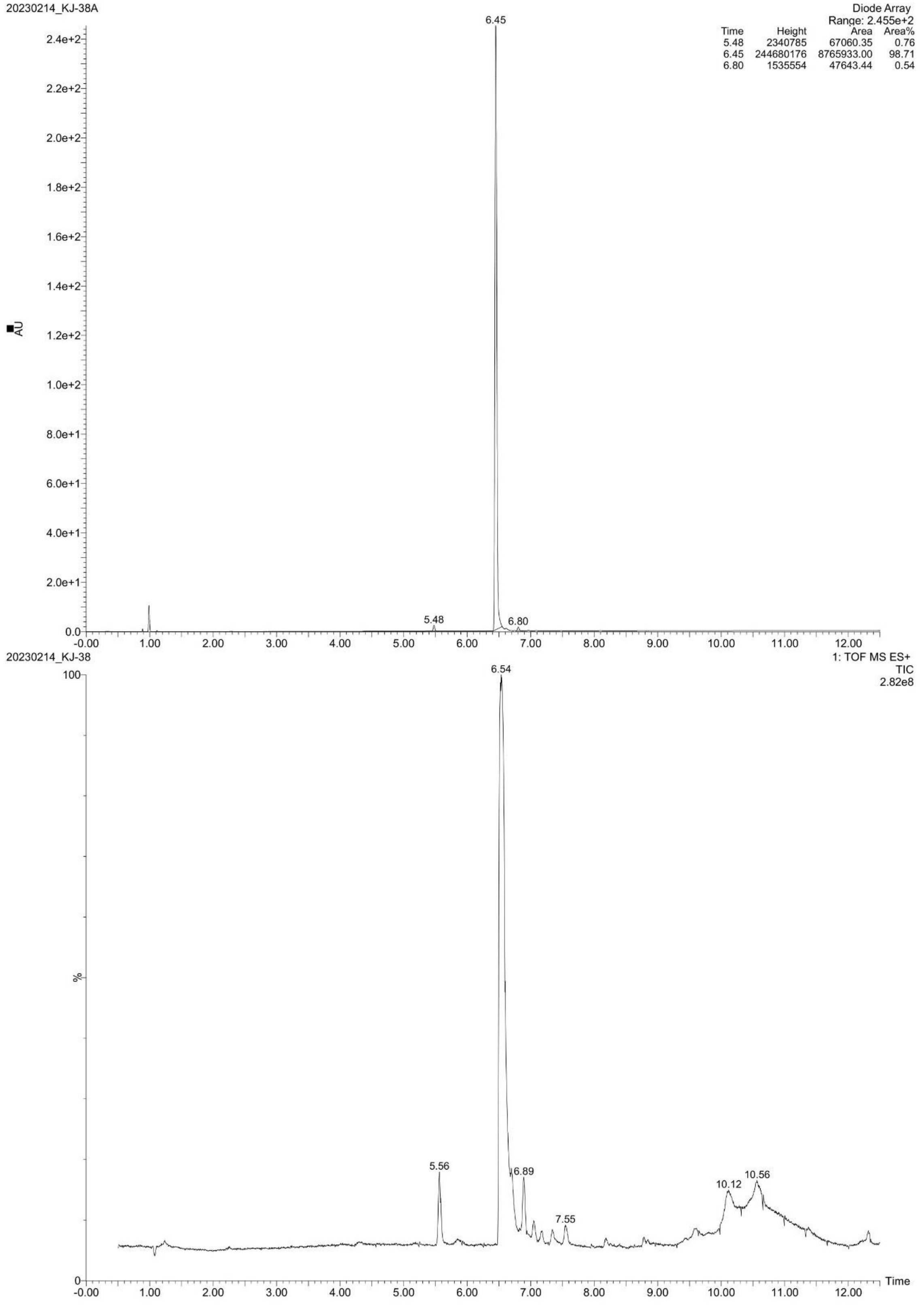

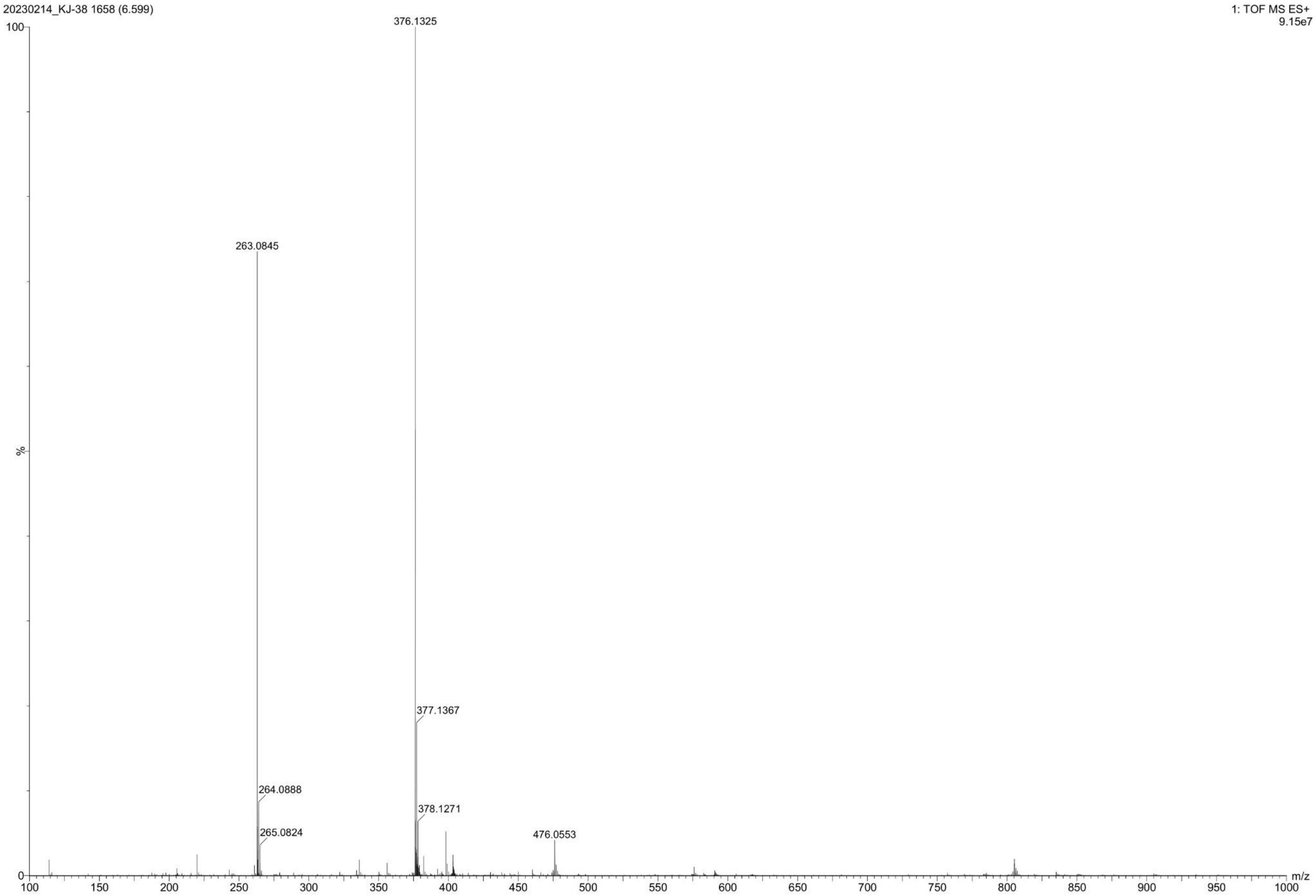
20230214_KJ-38 1658 (6.599)
1: TOF MS ES+
9.15e7
376.1325
263.0845
377.1367
264.0888
378.1271
265.0824
476.0553
%
m/z

^{1}H NMR and ^{13}C NMR spectra for the final compounds

N-(1-oxo-1-(4-phenylpiperazin-1-yl)propan-2-yl)acetamide (**21**) – ^{1}H NMR

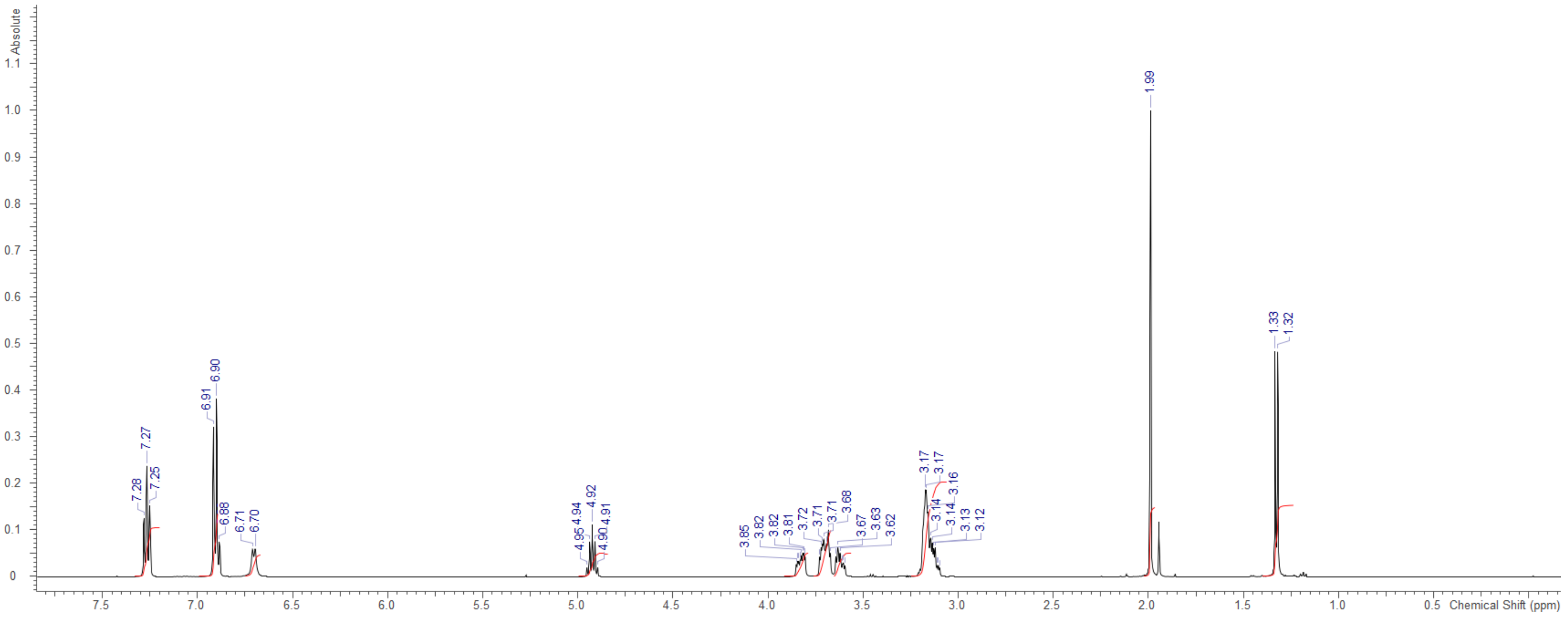

N-(1-oxo-1-(4-phenylpiperazin-1-yl)propan-2-yl)acetamide (**21**) – ^{13}C NMR

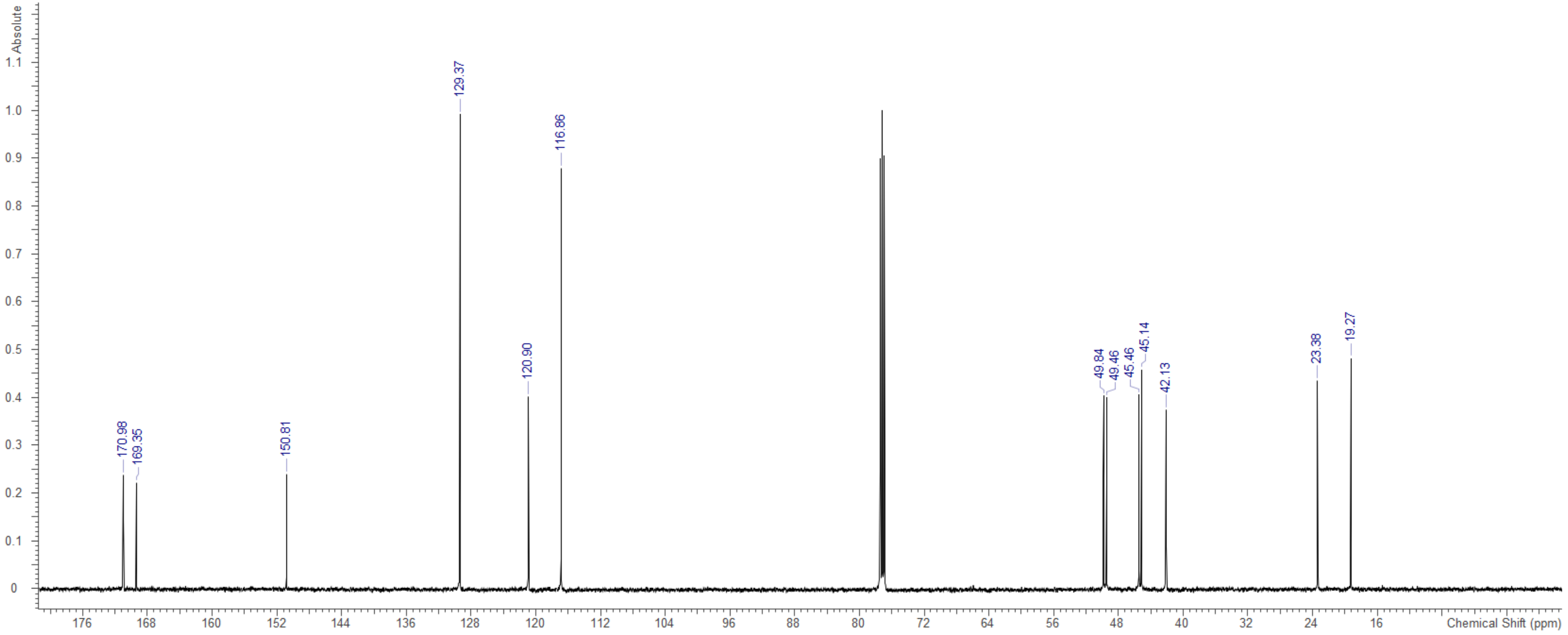

N-(1-(4-(3-chlorophenyl)piperazin-1-yl)-1-oxopropan-2-yl)acetamide (**22**) – ^{1}H NMR

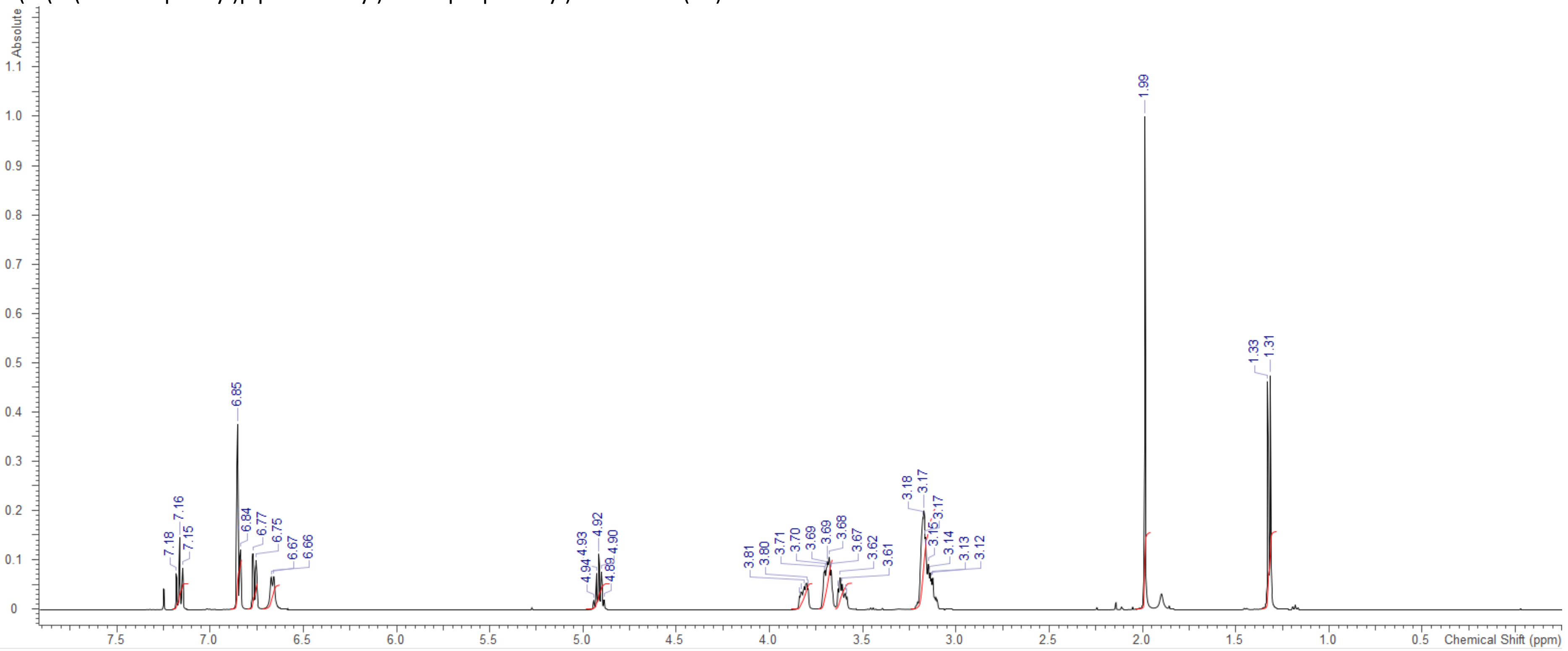

N-(1-(4-(3-chlorophenyl)piperazin-1-yl)-1-oxopropan-2-yl)acetamide (**22**) – ^{13}C NMR

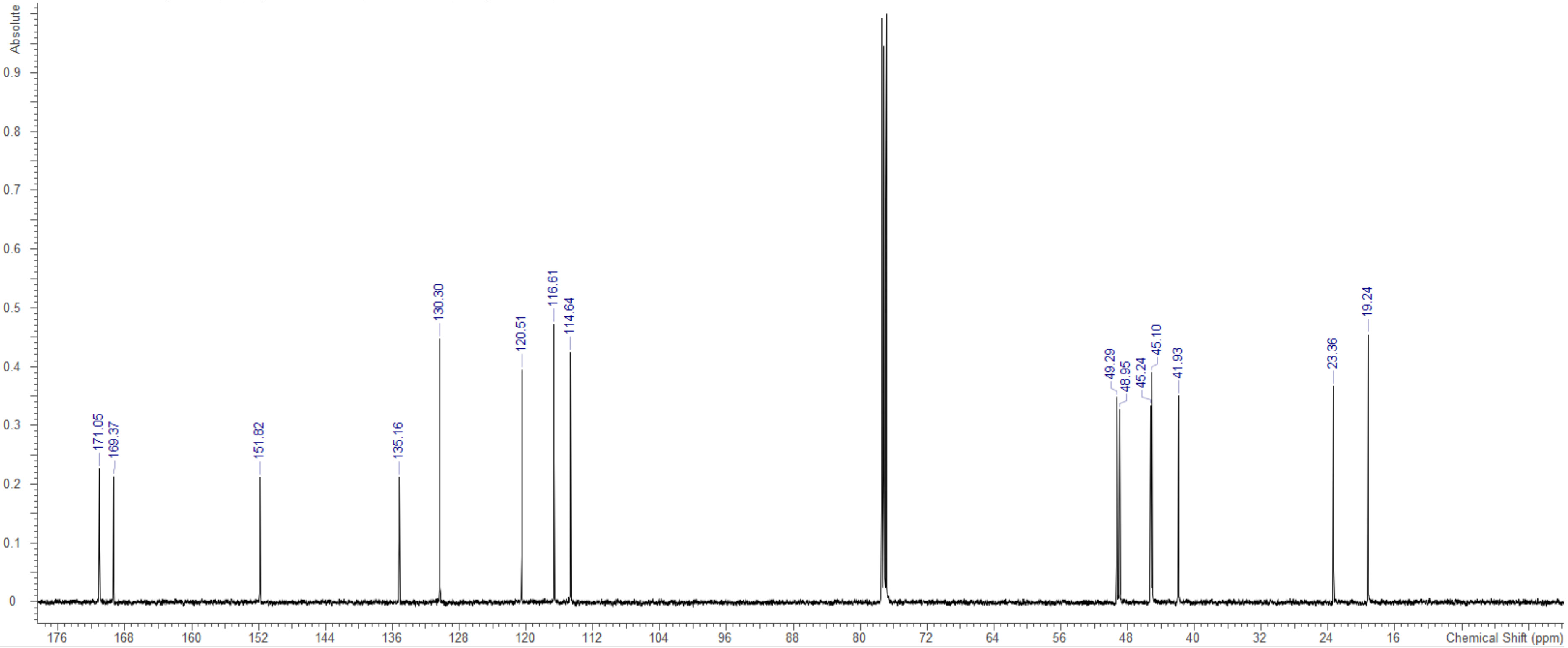

N-(1-(4-(4-chlorophenyl)piperazin-1-yl)-1-oxopropan-2-yl)acetamide (**23**) – ^{1}H NMR

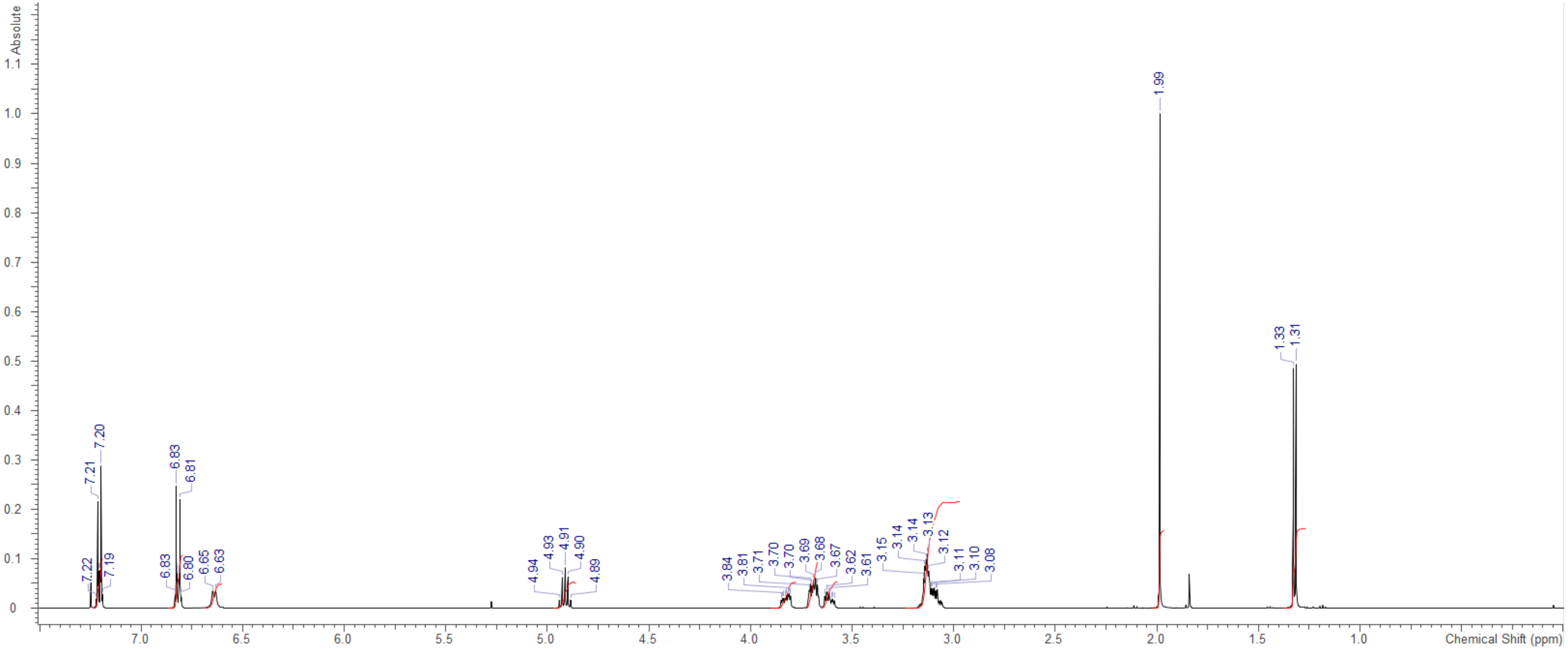

N-(1-(4-(4-chlorophenyl)piperazin-1-yl)-1-oxopropan-2-yl)acetamide (**23**) – ^{13}C NMR

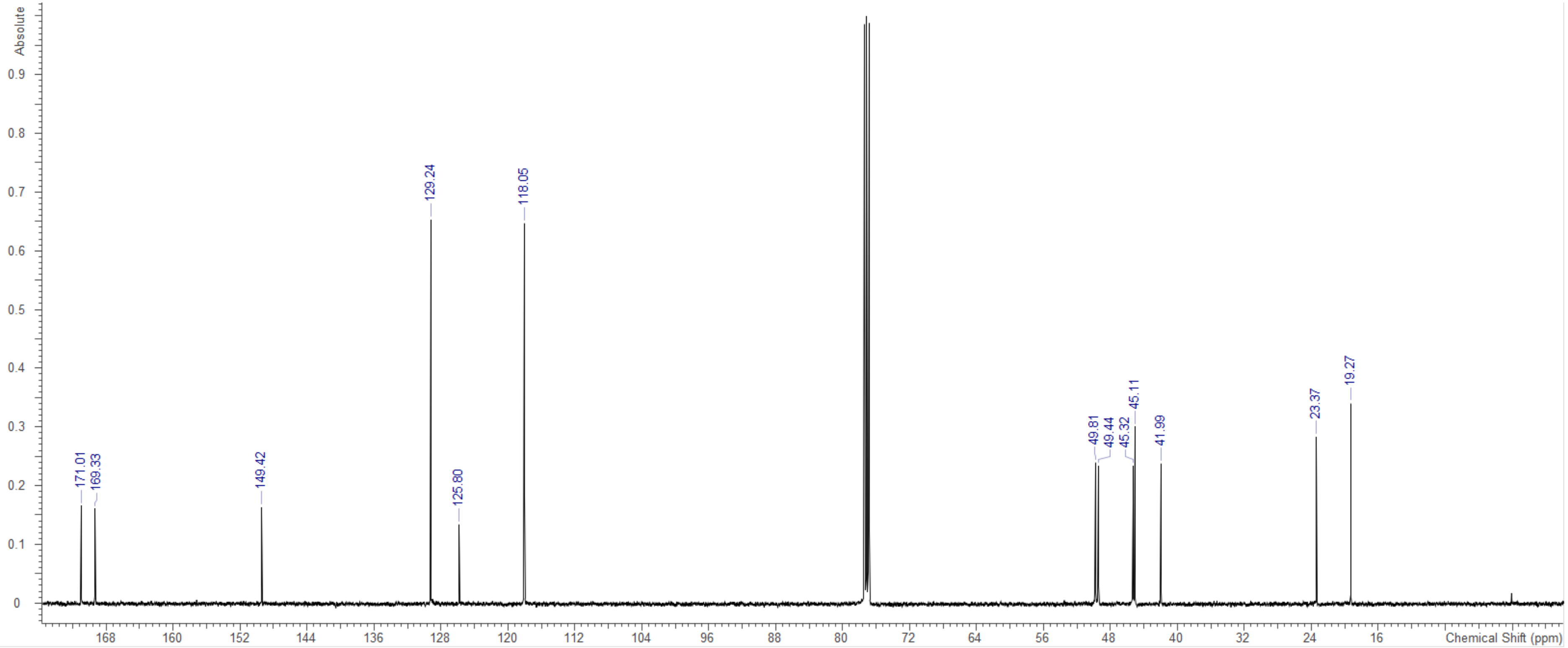

N-(1-(4-(3,4-dichlorophenyl)piperazin-1-yl)-1-oxopropan-2-yl)acetamide (**24**) – ^{1}H NMR

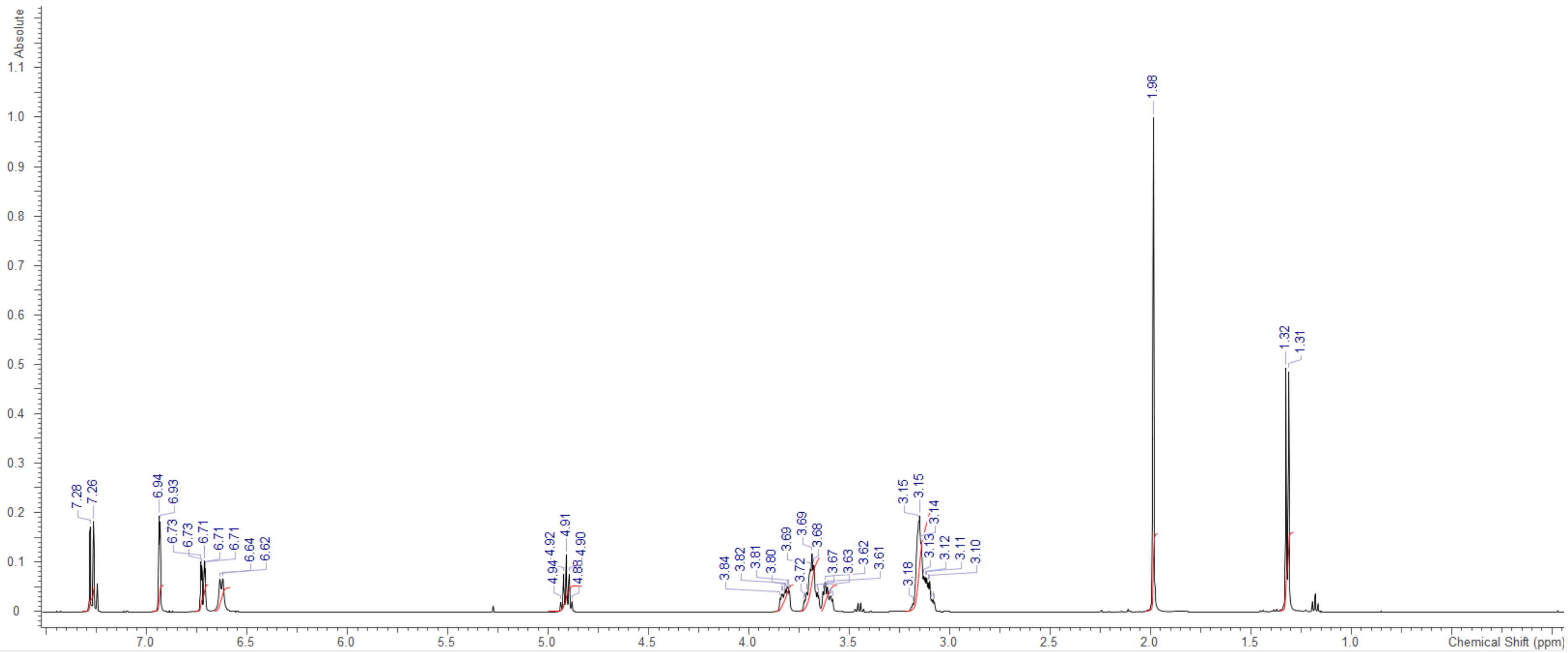

N-(1-(4-(3,4-dichlorophenyl)piperazin-1-yl)-1-oxopropan-2-yl)acetamide (**24**) – ^{13}C NMR

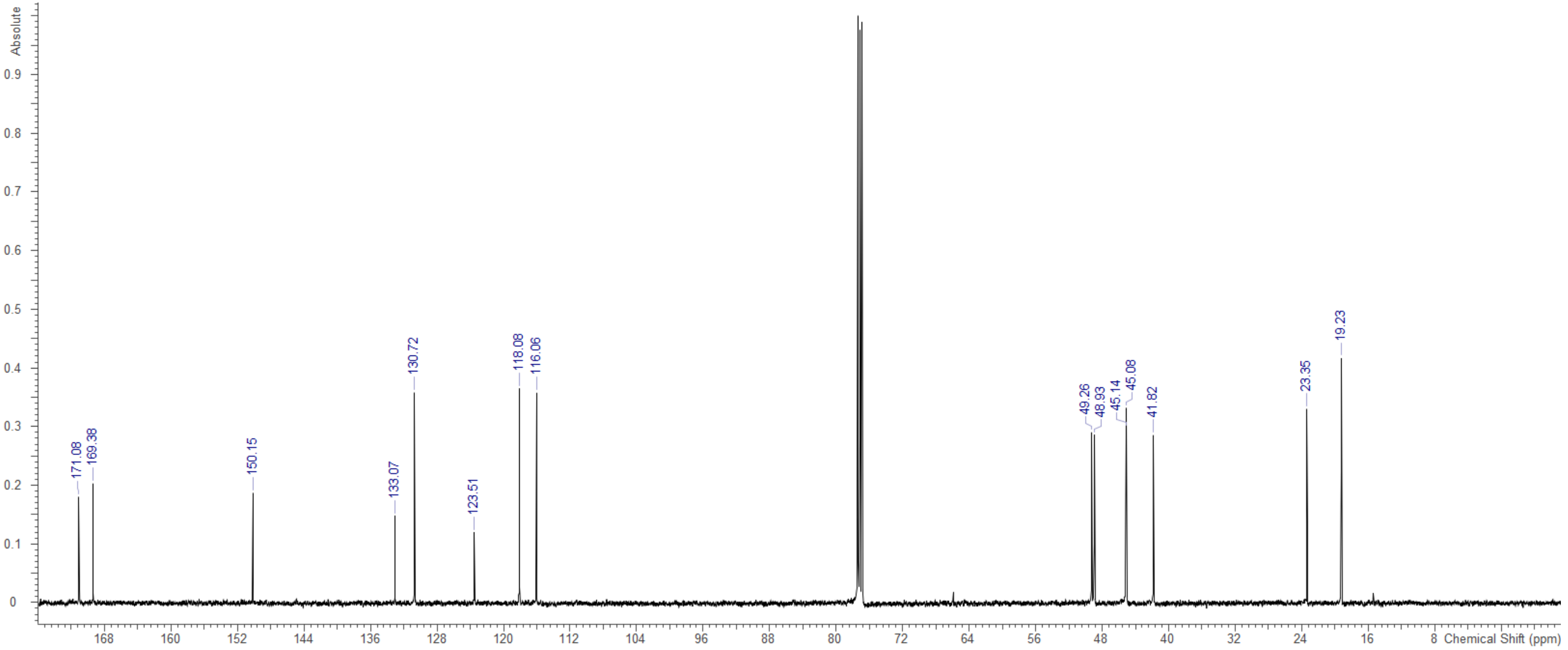

N-(1-(4-(3,5-dichlorophenyl)piperazin-1-yl)-1-oxopropan-2-yl)acetamide (**25**) – ^{1}H NMR

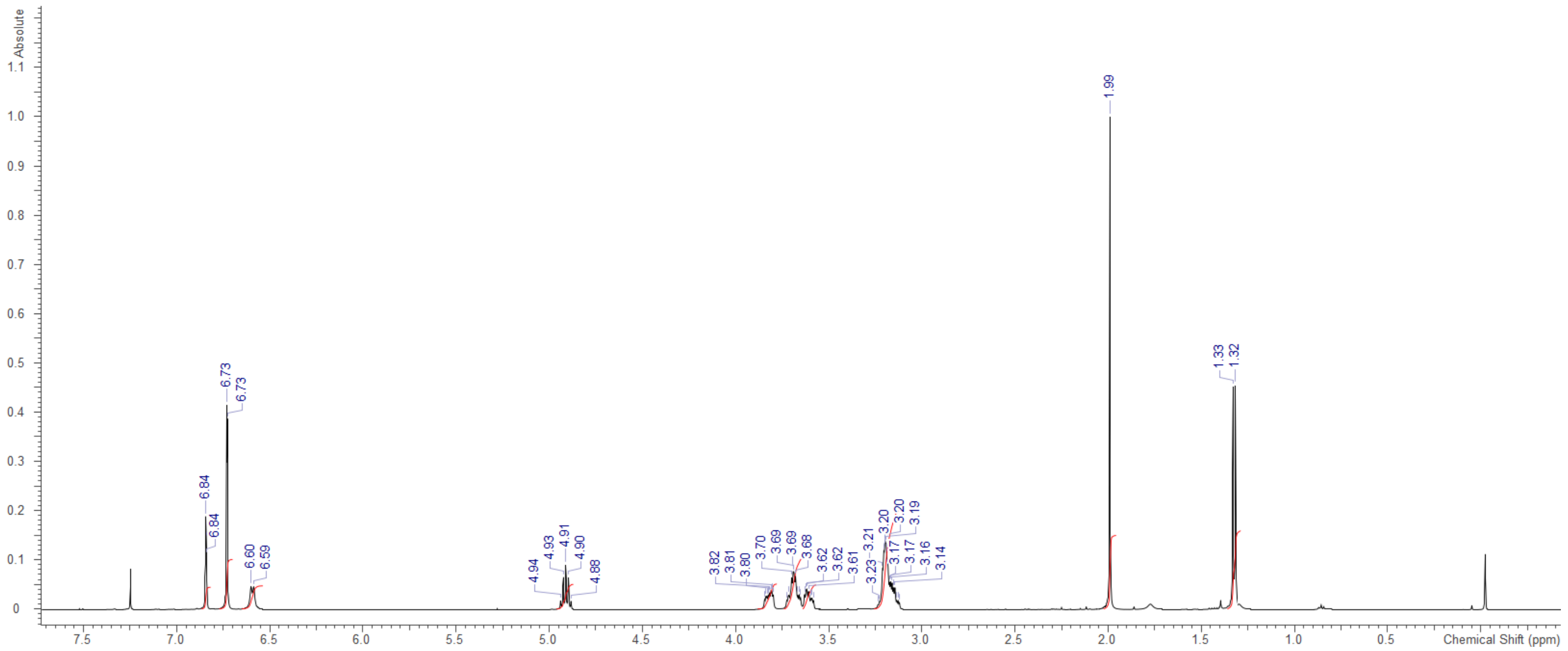

N-(1-(4-(3,5-dichlorophenyl)piperazin-1-yl)-1-oxopropan-2-yl)acetamide (**25**) – ^{13}C NMR

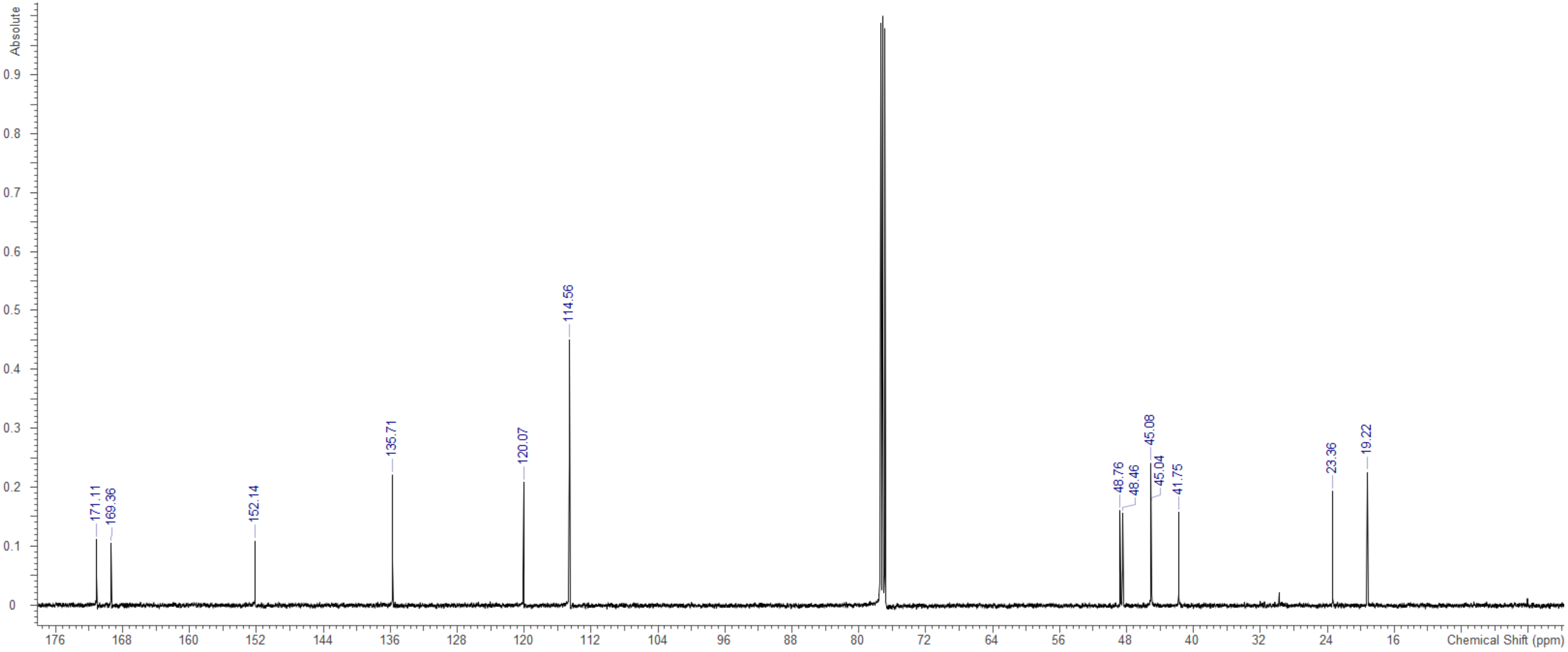

N-(1-oxo-1-(4-(3-(trifluoromethyl)phenyl)piperazin-1-yl)propan-2-yl)acetamide (**26**) – ^{1}H NMR

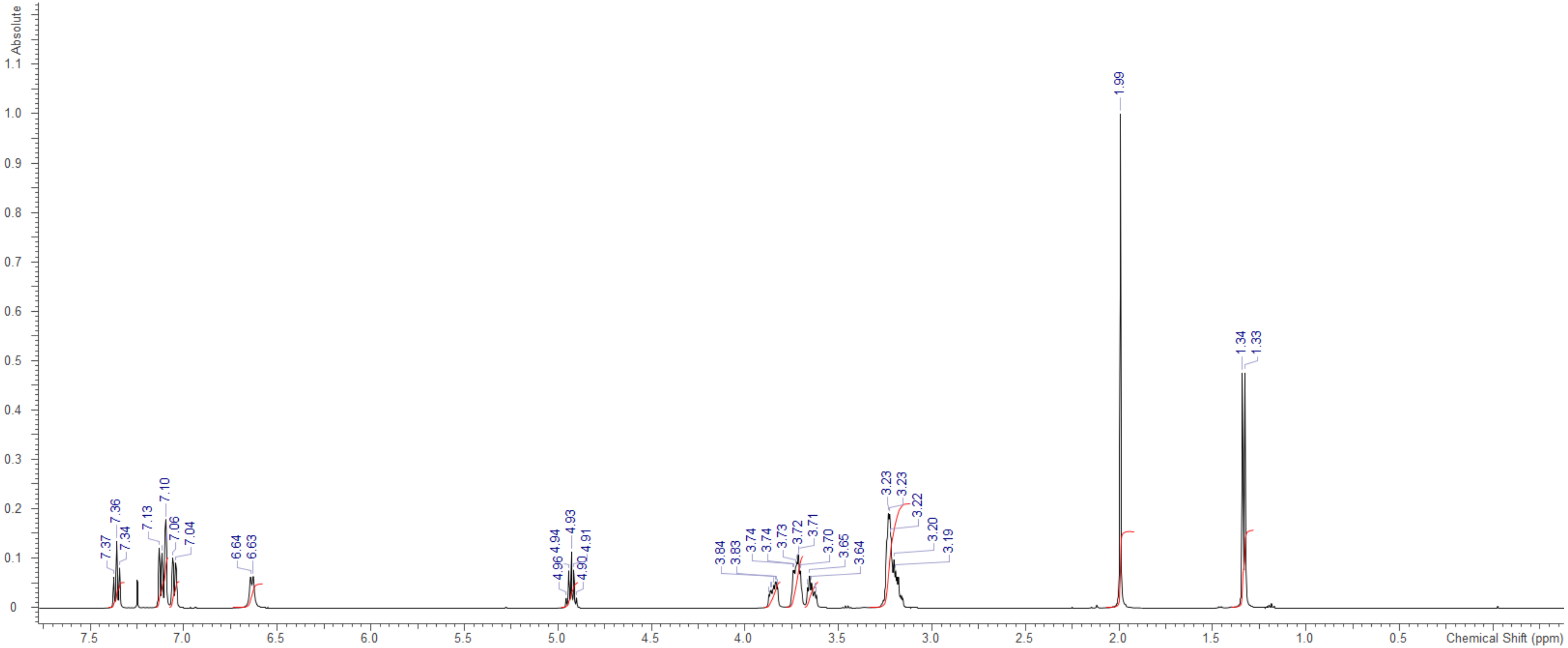

N-(1-oxo-1-(4-(3-(trifluoromethyl)phenyl)piperazin-1-yl)propan-2-yl)acetamide (**26**) – ^{13}C NMR

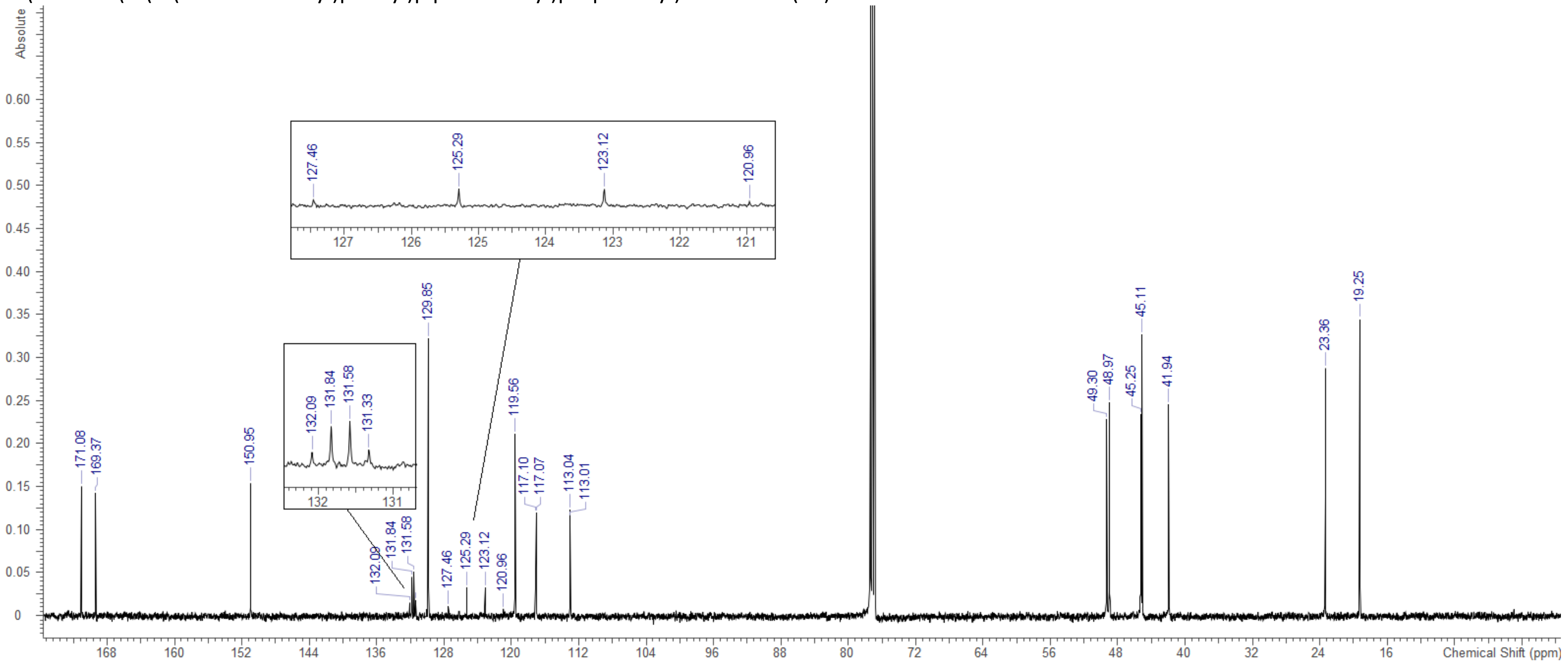

N-(1-oxo-1-(4-(4-(trifluoromethyl)phenyl)piperazin-1-yl)propan-2-yl)acetamide (**27**) – ^{1}H NMR

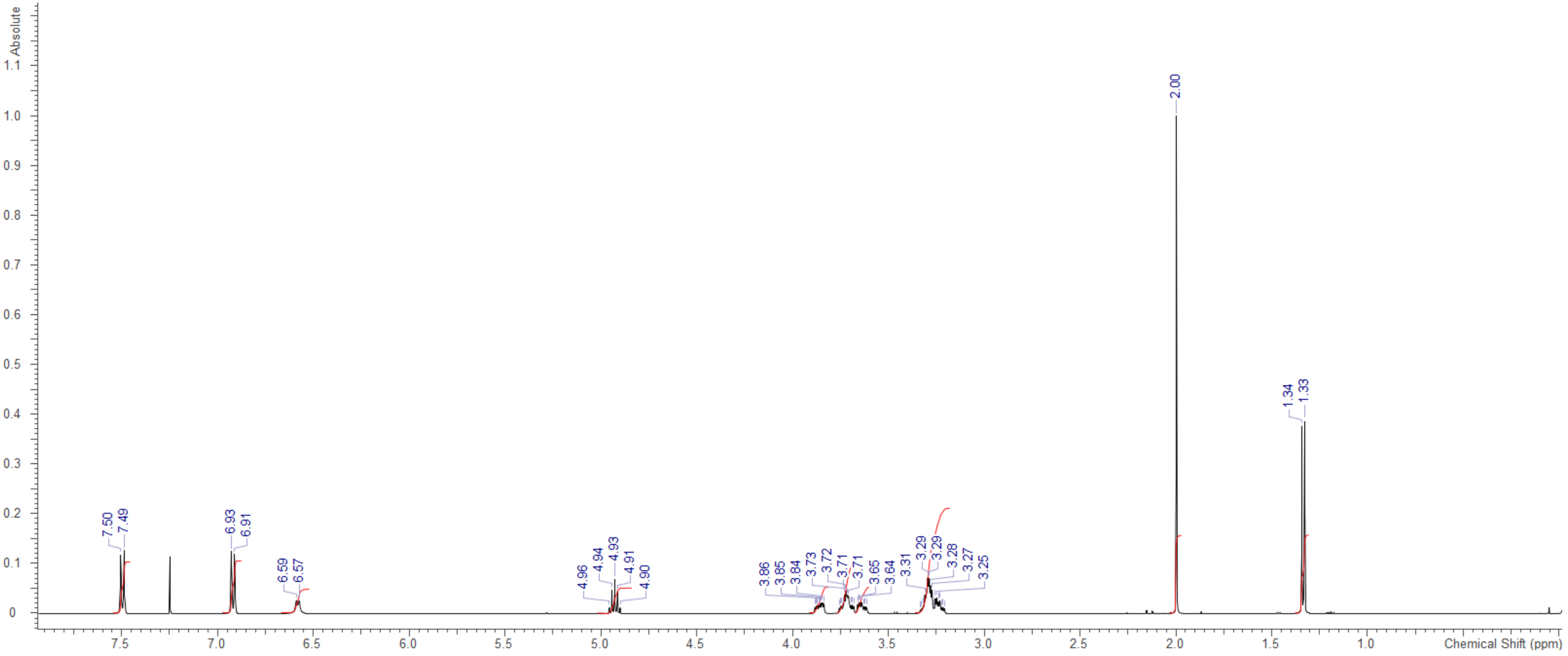

N-(1-oxo-1-(4-(4-(trifluoromethyl)phenyl)piperazin-1-yl)propan-2-yl)acetamide (**27**) – ^{13}C NMR

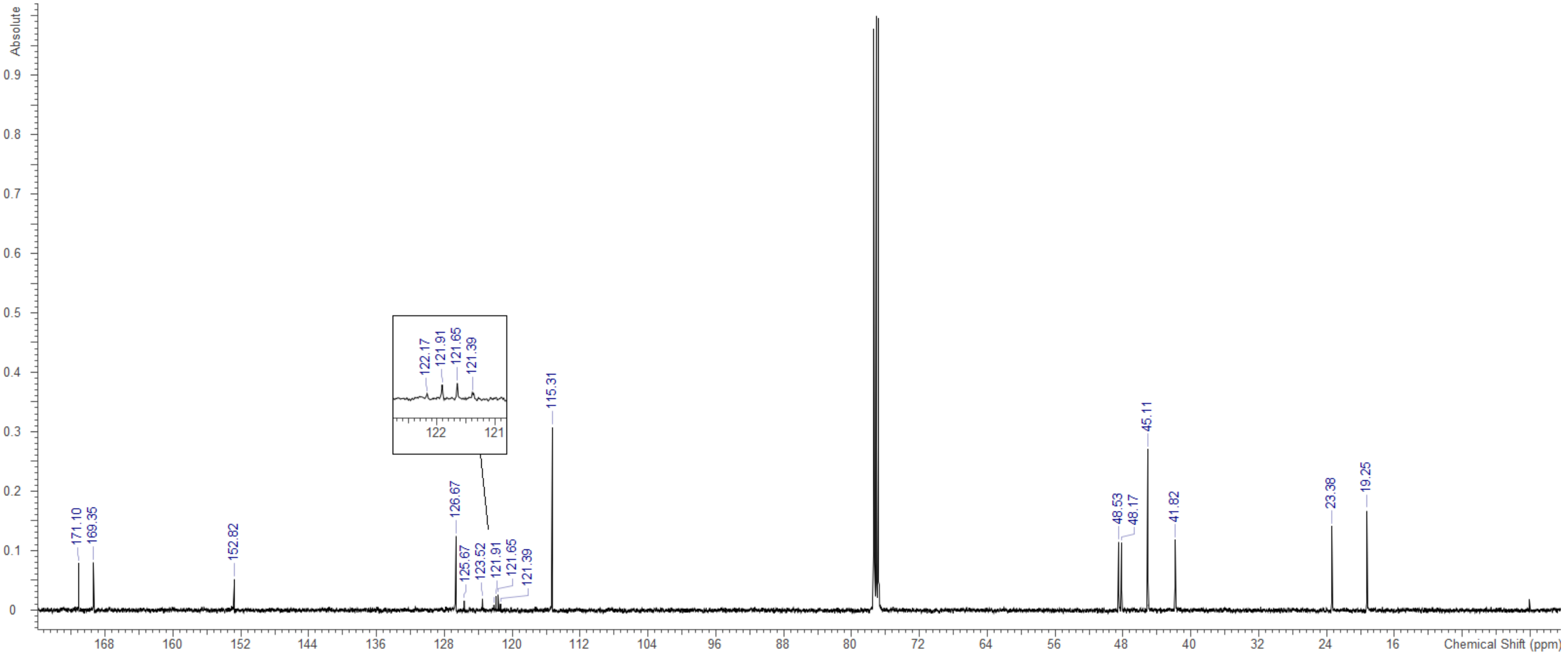

N-(1-oxo-1-(4-(3-(trifluoromethoxy)phenyl)piperazin-1-yl)propan-2-yl)acetamide (**28**) – ^{1}H NMR

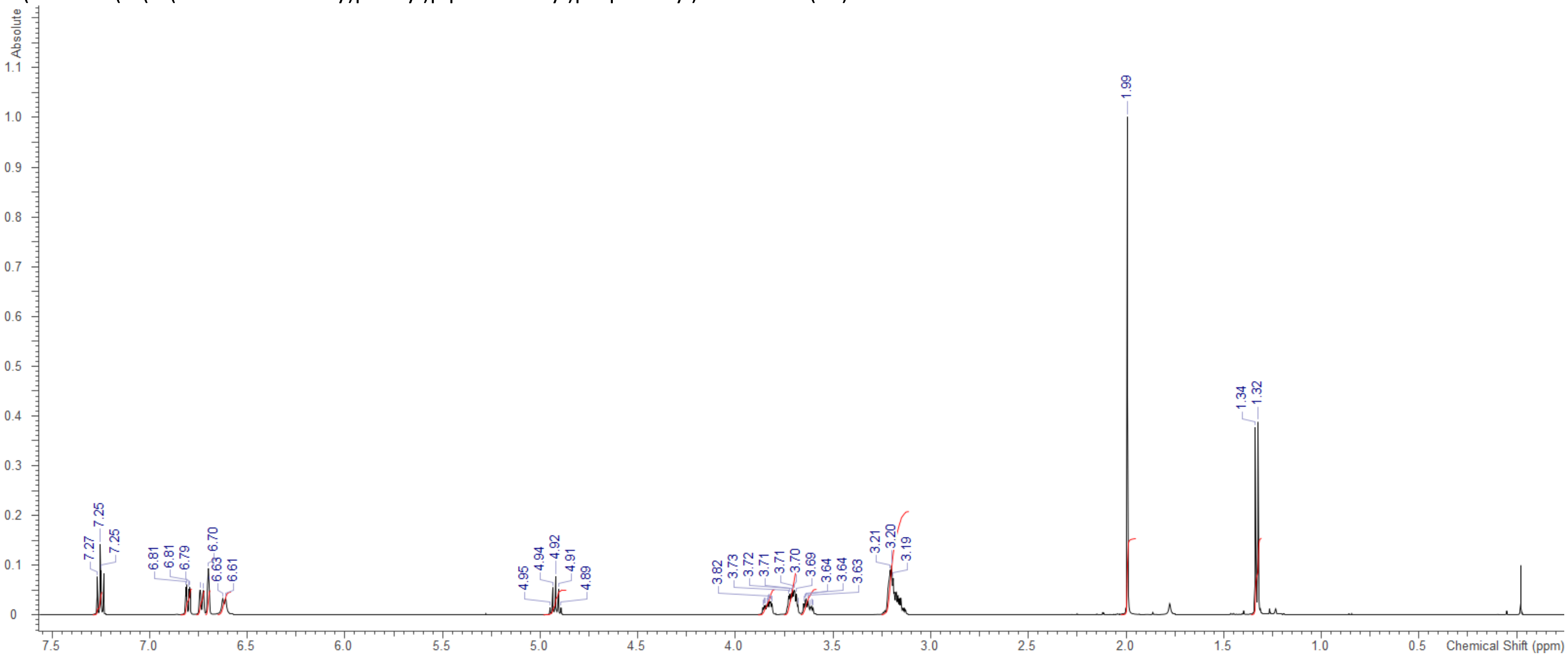

N-(1-oxo-1-(4-(3-(trifluoromethoxy)phenyl)piperazin-1-yl)propan-2-yl)acetamide (**28**) – ^{13}C NMR

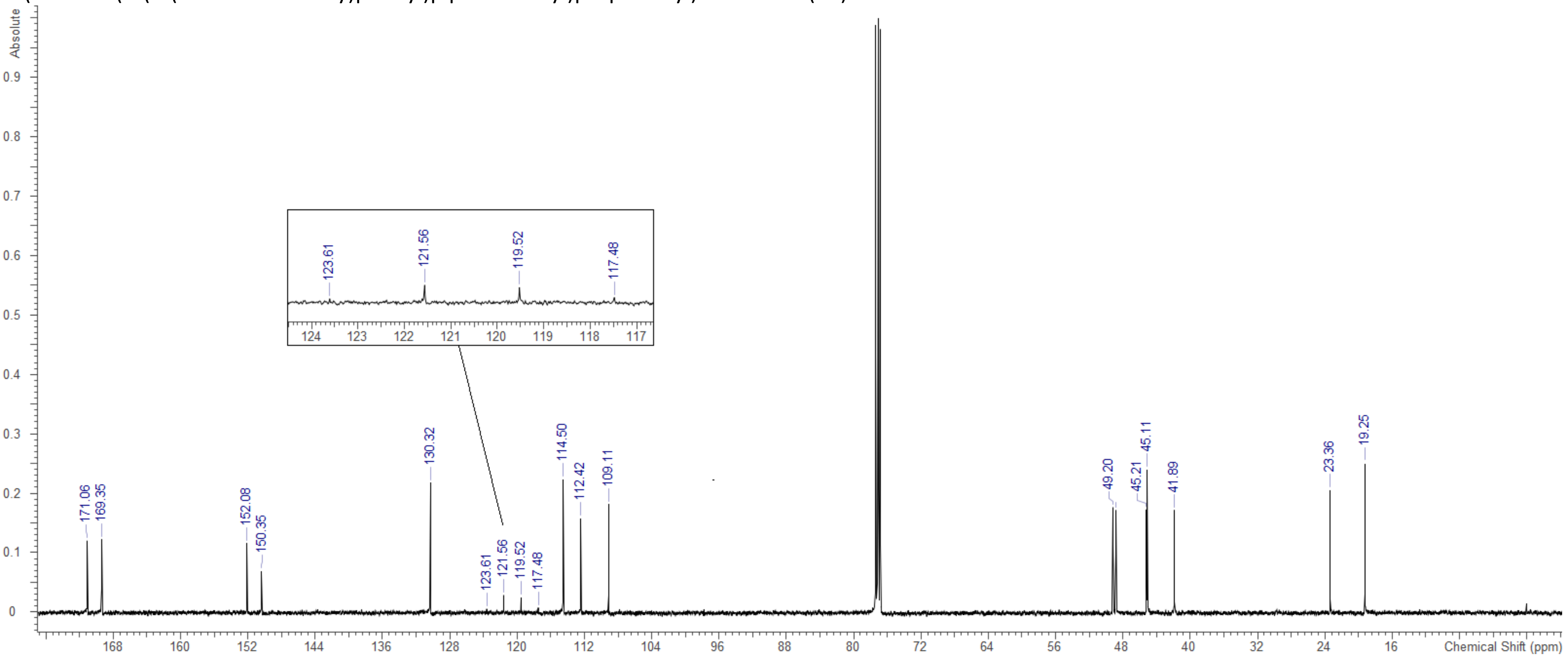

N-(1-oxo-1-(4-(3-phenoxyphenyl)piperazin-1-yl)propan-2-yl)acetamide (**29**) – ^{1}H NMR

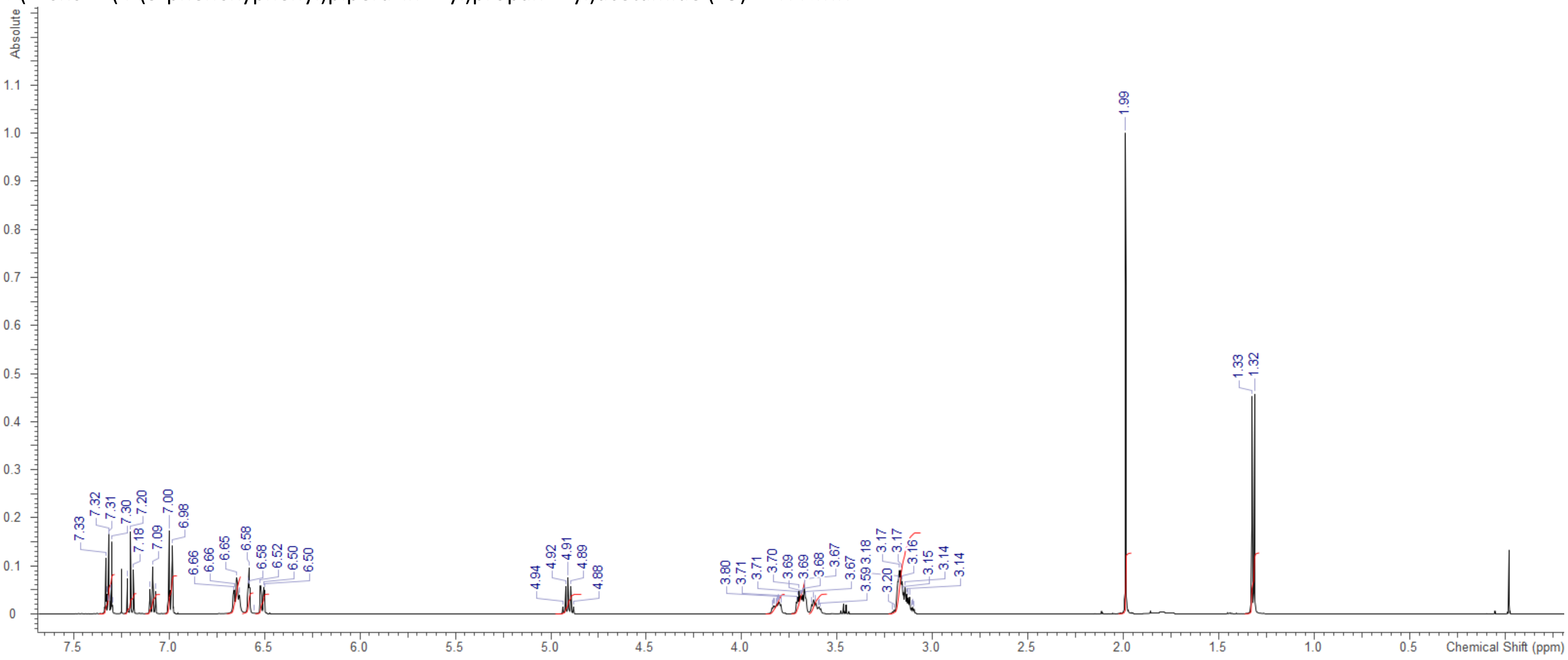

N-(1-oxo-1-(4-(3-phenoxyphenyl)piperazin-1-yl)propan-2-yl)acetamide (**29**) – ^{13}C NMR

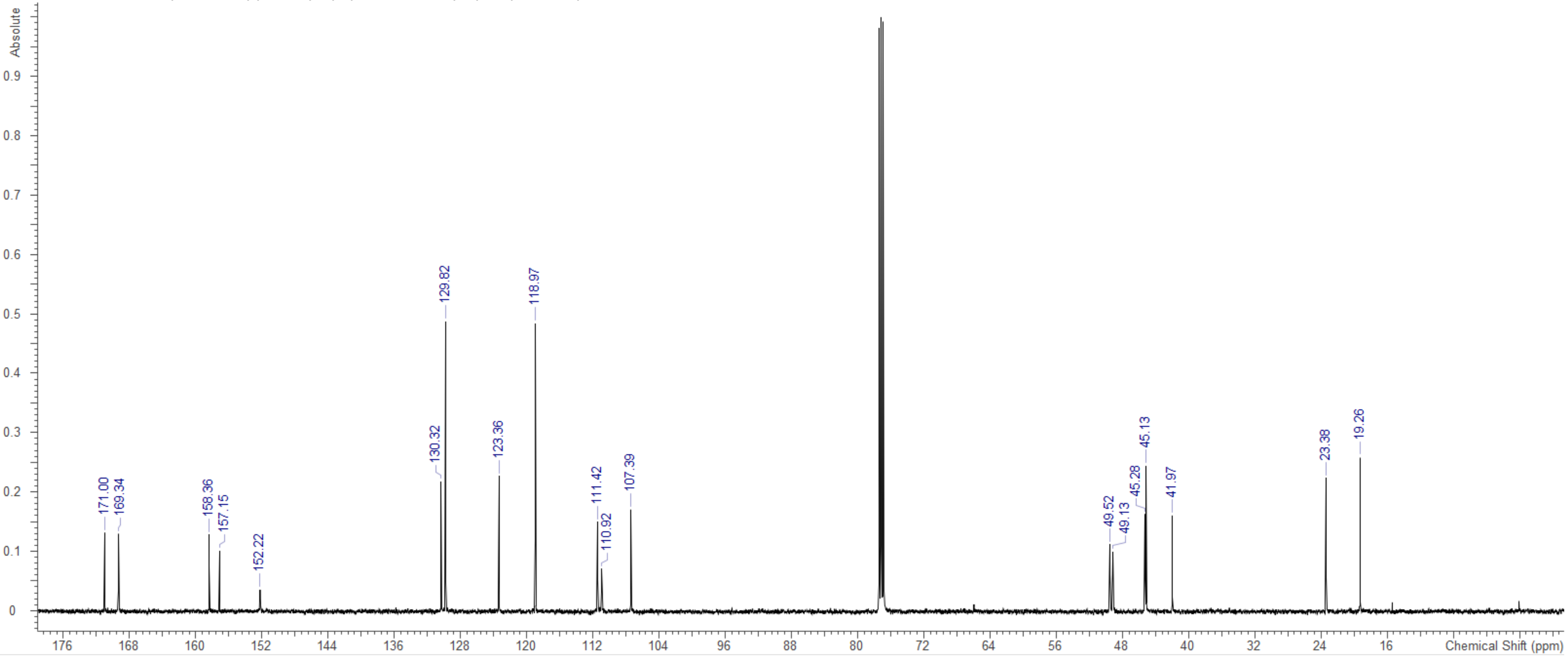

N-(1-oxo-1-(4-(3-((trifluoromethyl)thio)phenyl)piperazin-1-yl)propan-2-yl)acetamide (**30**) – ^{1}H NMR

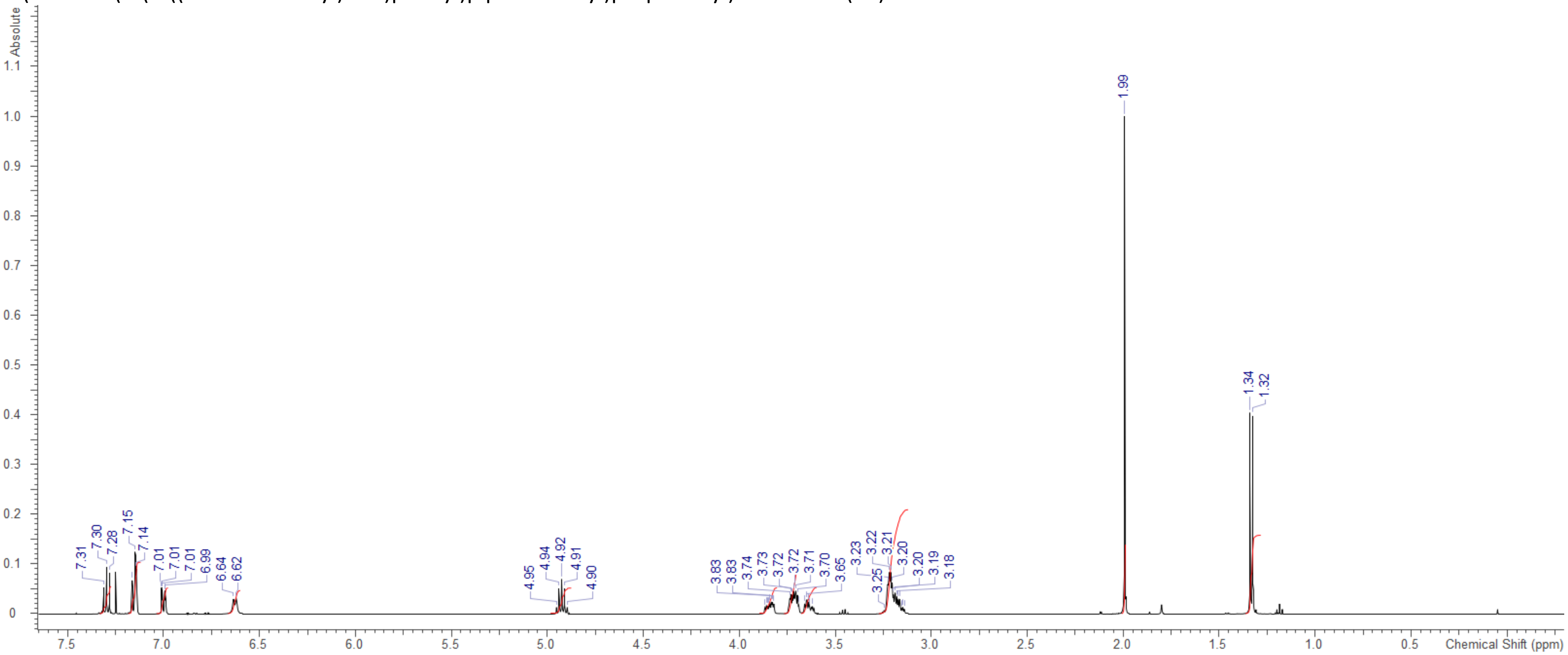

N-(1-oxo-1-(4-(3-((trifluoromethyl)thio)phenyl)piperazin-1-yl)propan-2-yl)acetamide (**30**) – ^{13}C NMR

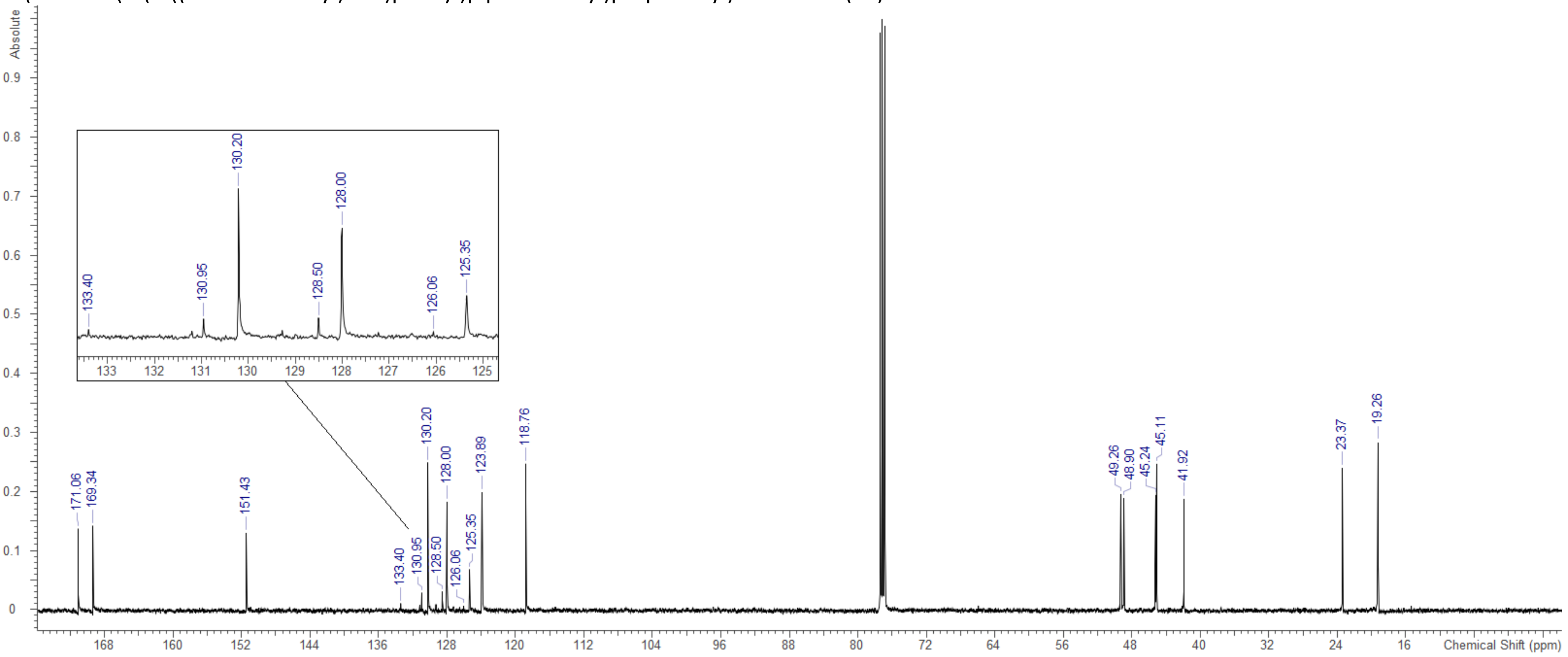

N-(1-(4-(3-chlorophenyl)piperazin-1-yl)-1-oxopropan-2-yl)benzamide (**31**) – ^{1}H NMR

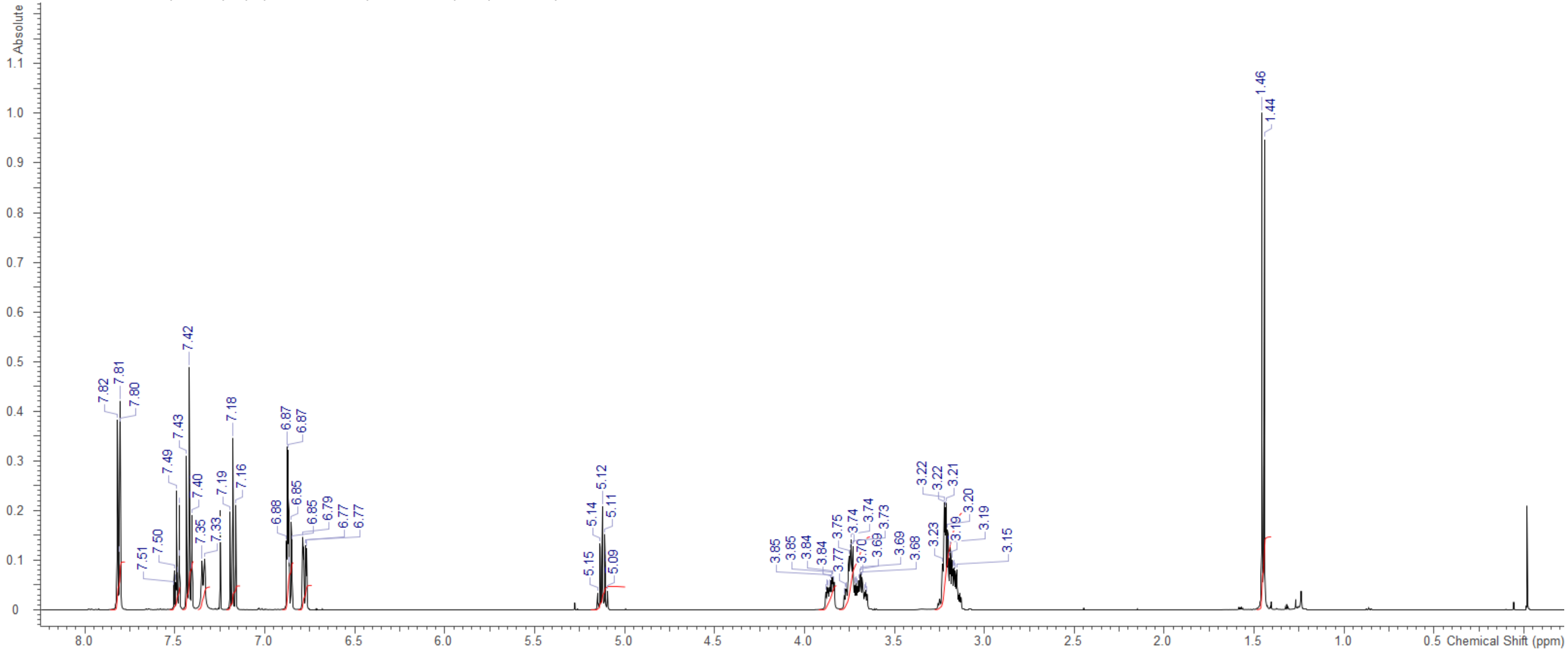

N-(1-(4-(3-chlorophenyl)piperazin-1-yl)-1-oxopropan-2-yl)benzamide (**31**) – ^{13}C NMR

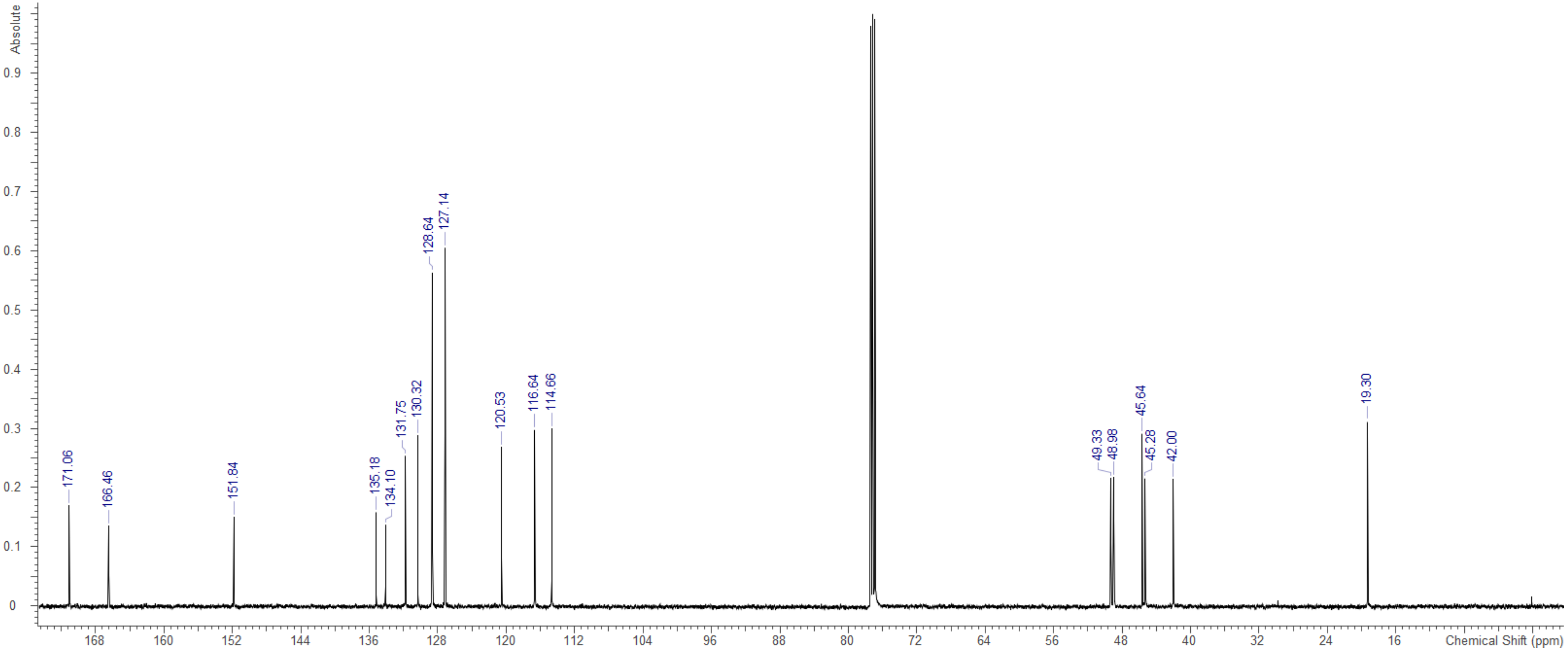

N-(1-oxo-1-(4-(3-(trifluoromethyl)phenyl)piperazin-1-yl)propan-2-yl)benzamide (**32**) – ^{1}H NMR

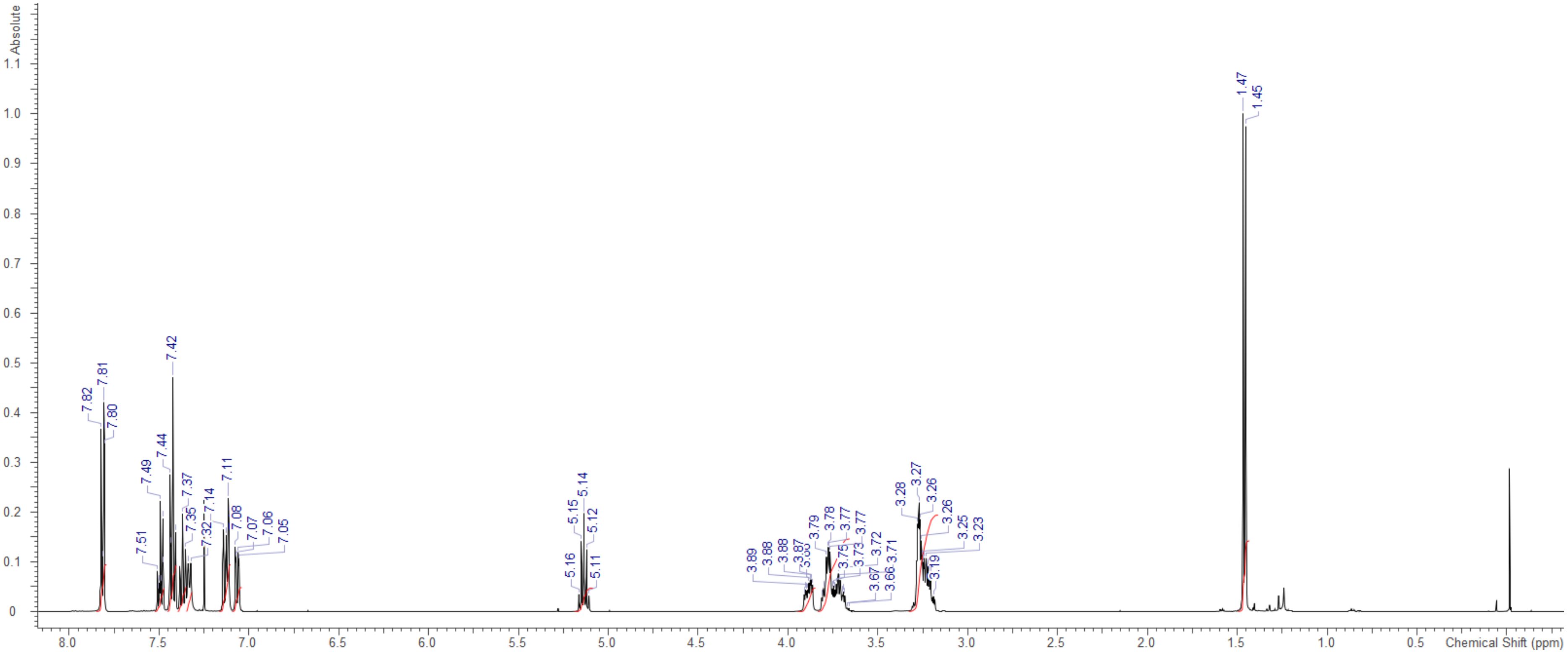

N-(1-oxo-1-(4-(3-(trifluoromethyl)phenyl)piperazin-1-yl)propan-2-yl)benzamide (**32**) – ^{13}C NMR

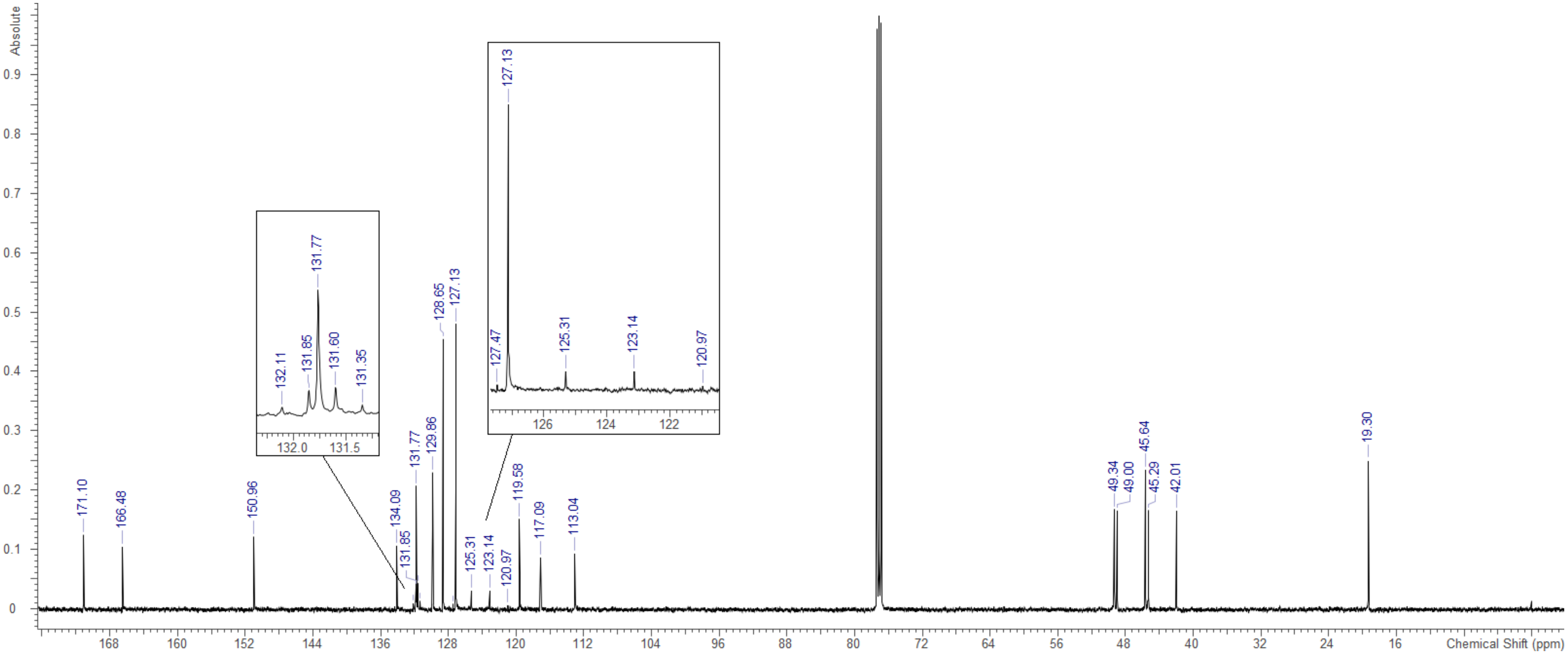

1-(1-(4-(3-chlorophenyl)piperazin-1-yl)-1-oxopropan-2-yl)-3-ethylurea (**33**) – ^{1}H NMR

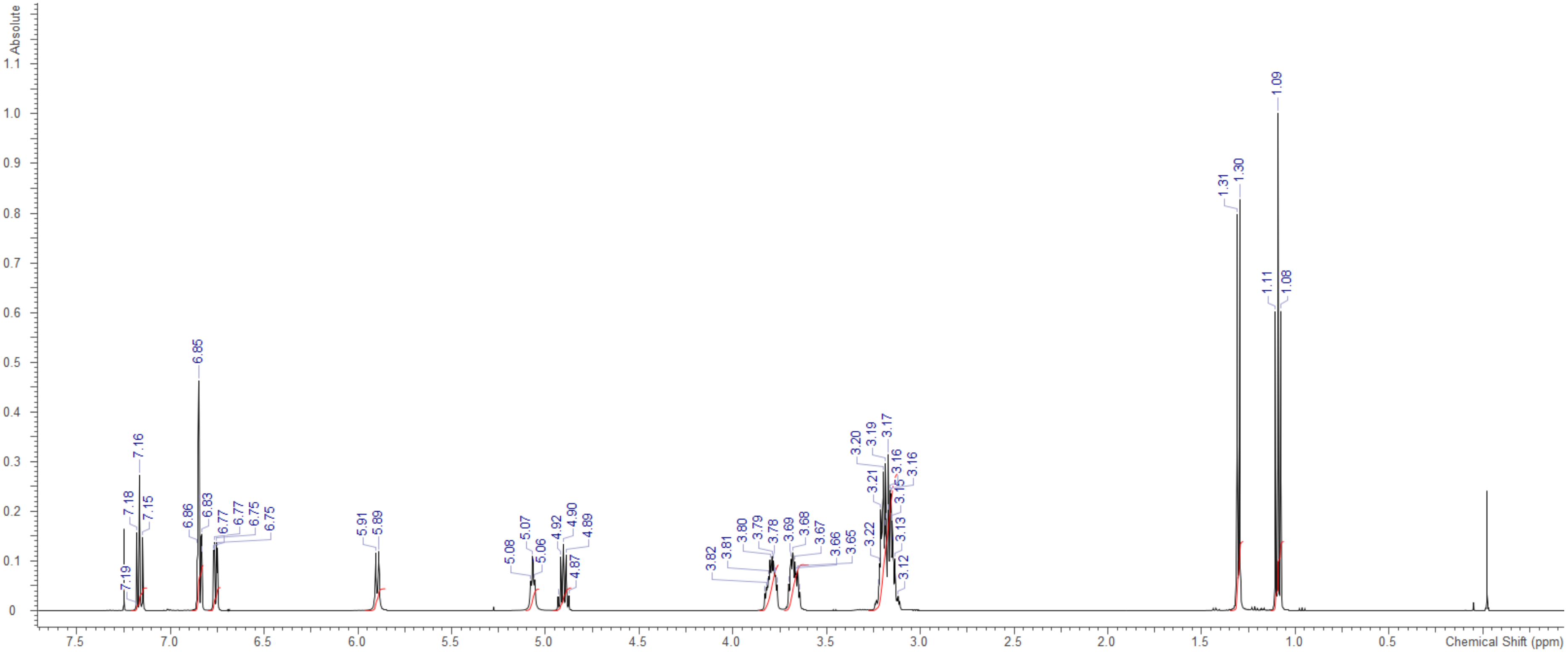

1-(1-(4-(3-chlorophenyl)piperazin-1-yl)-1-oxopropan-2-yl)-3-ethylurea (**33**) – ^{13}C NMR

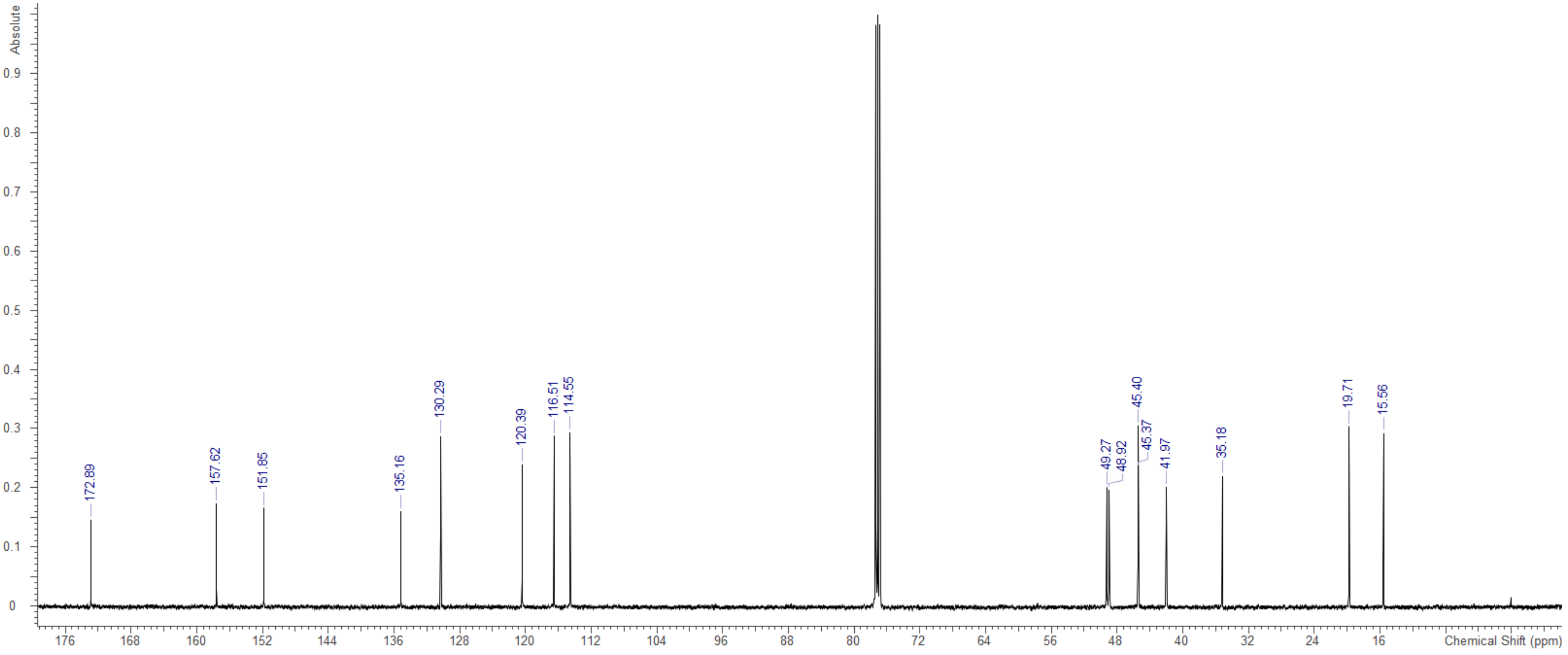

1-ethyl-3-(1-oxo-1-(4-(3-(trifluoromethyl)phenyl)piperazin-1-yl)propan-2-yl)urea (**34**) – ^{1}H NMR

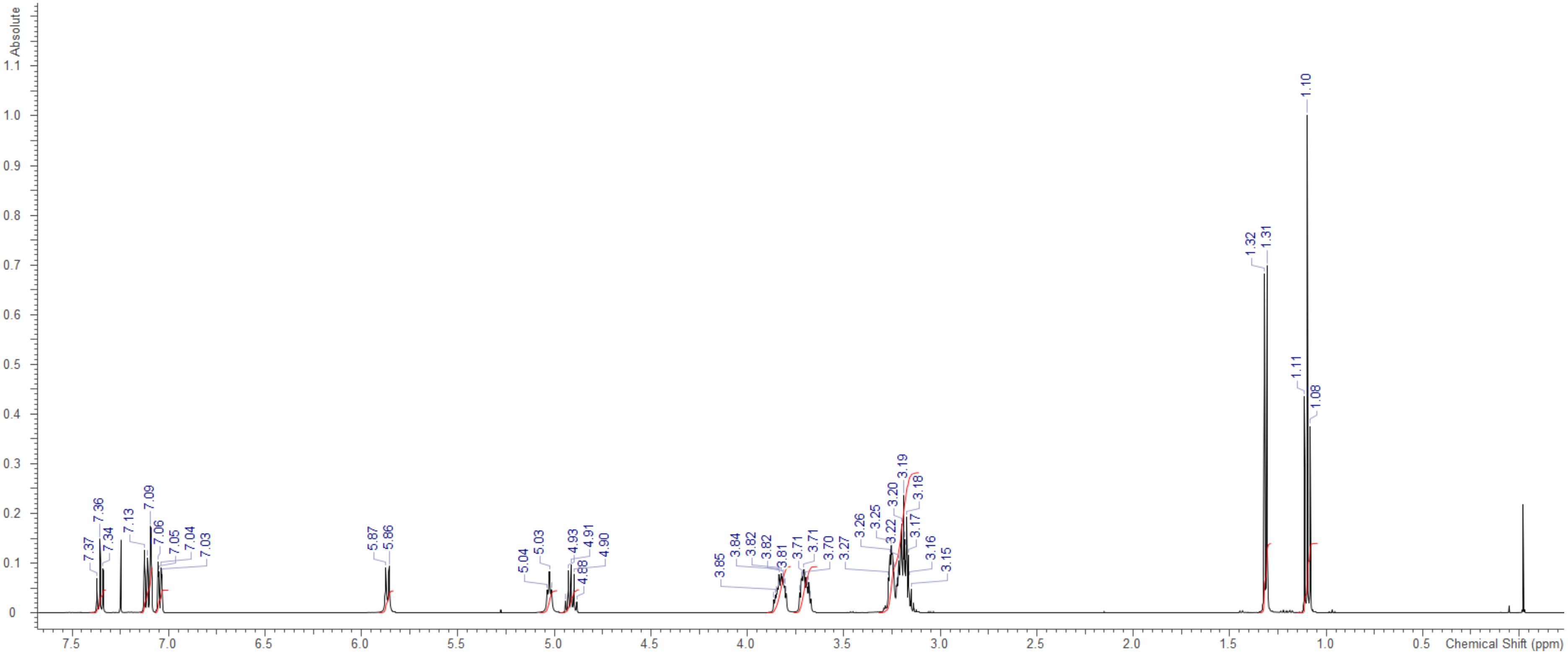

1-ethyl-3-(1-oxo-1-(4-(3-(trifluoromethyl)phenyl)piperazin-1-yl)propan-2-yl)urea (**34**) – ^{13}C NMR

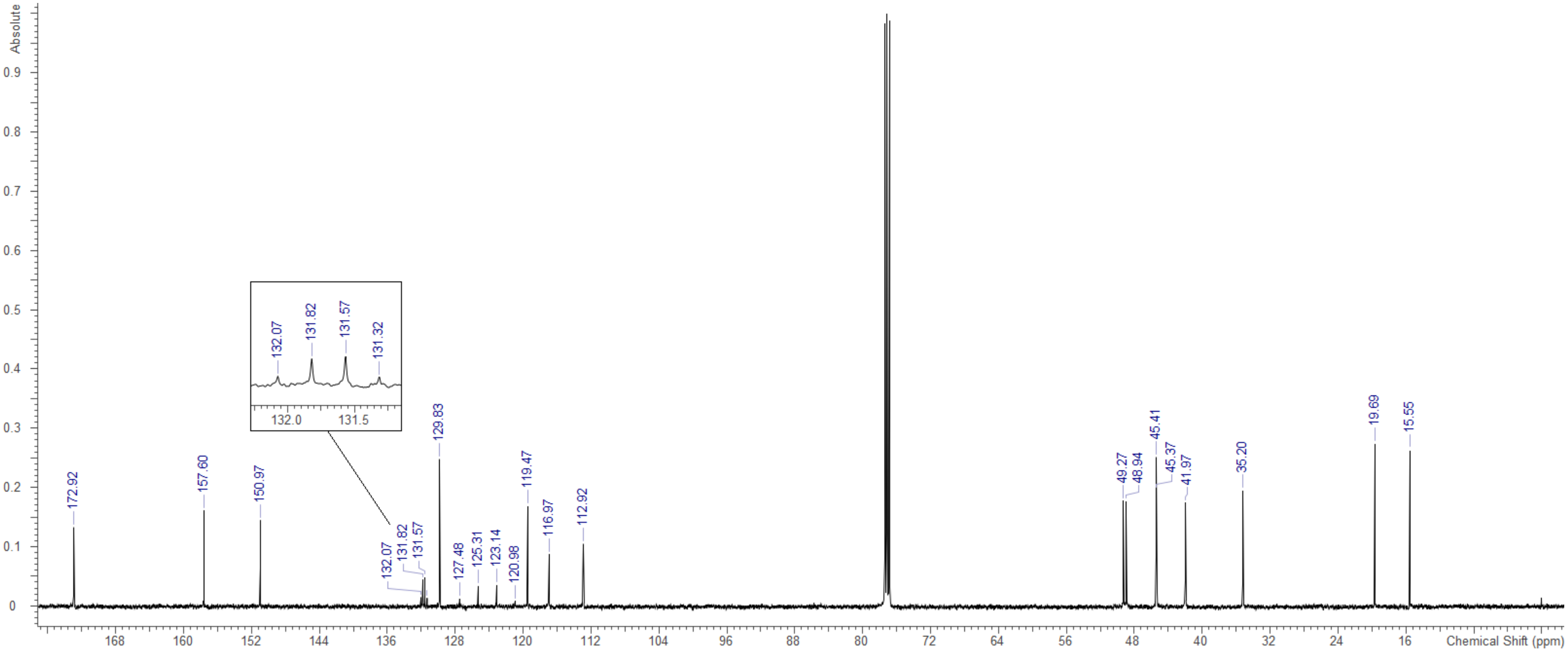

1-(1-(4-(3-chlorophenyl)piperazin-1-yl)-1-oxopropan-2-yl)-3-isopropylurea (**35**) – ^{1}H NMR

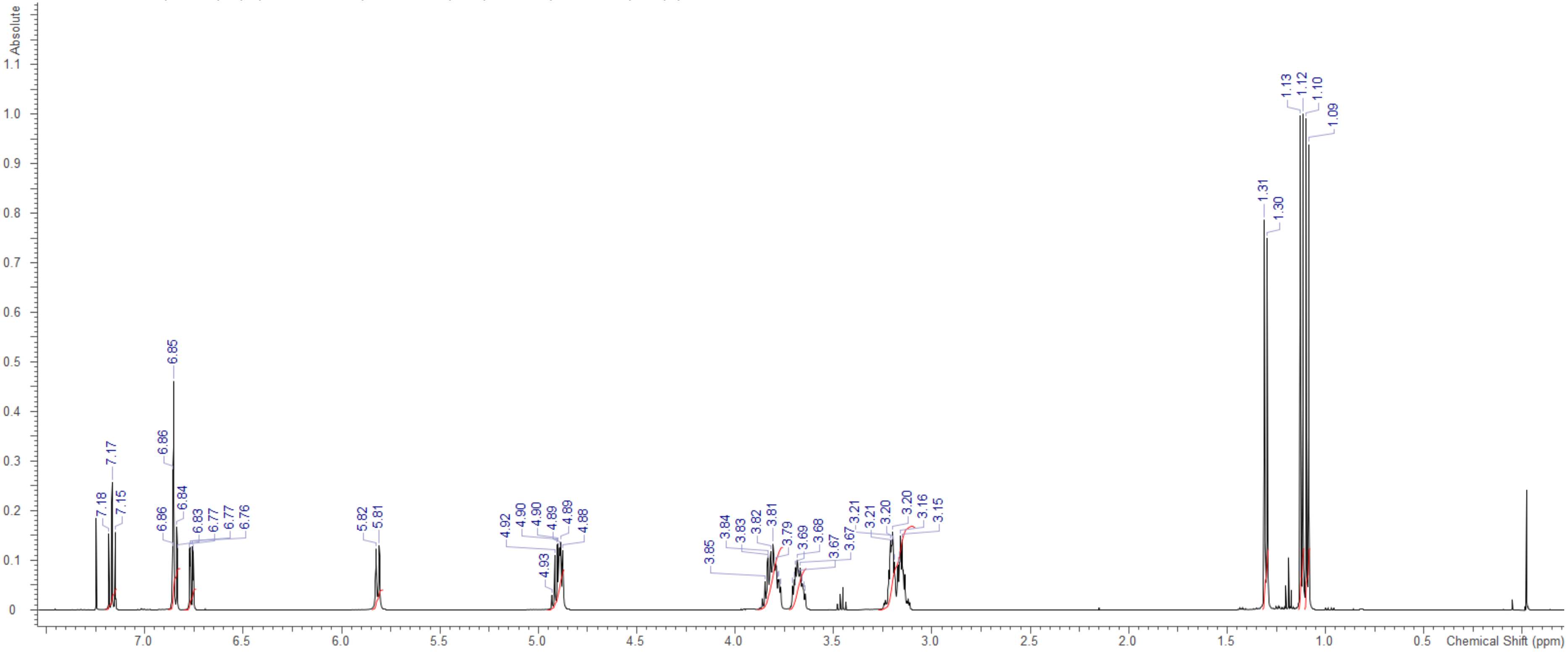

1-(1-(4-(3-chlorophenyl)piperazin-1-yl)-1-oxopropan-2-yl)-3-isopropylurea (**35**) – ^{13}C NMR

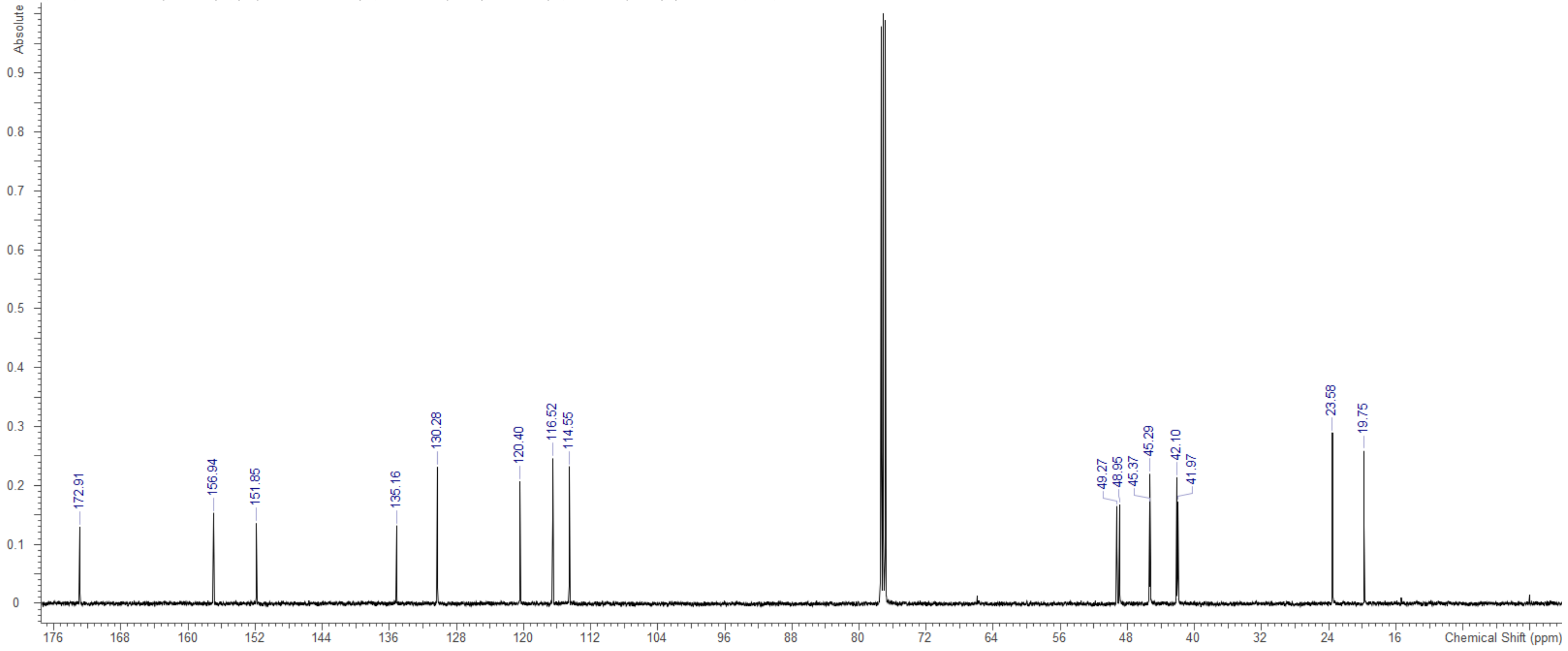

1-isopropyl-3-(1-oxo-1-(4-(3-(trifluoromethyl)phenyl)piperazin-1-yl)propan-2-yl)urea (**36**) – ^{1}H NMR

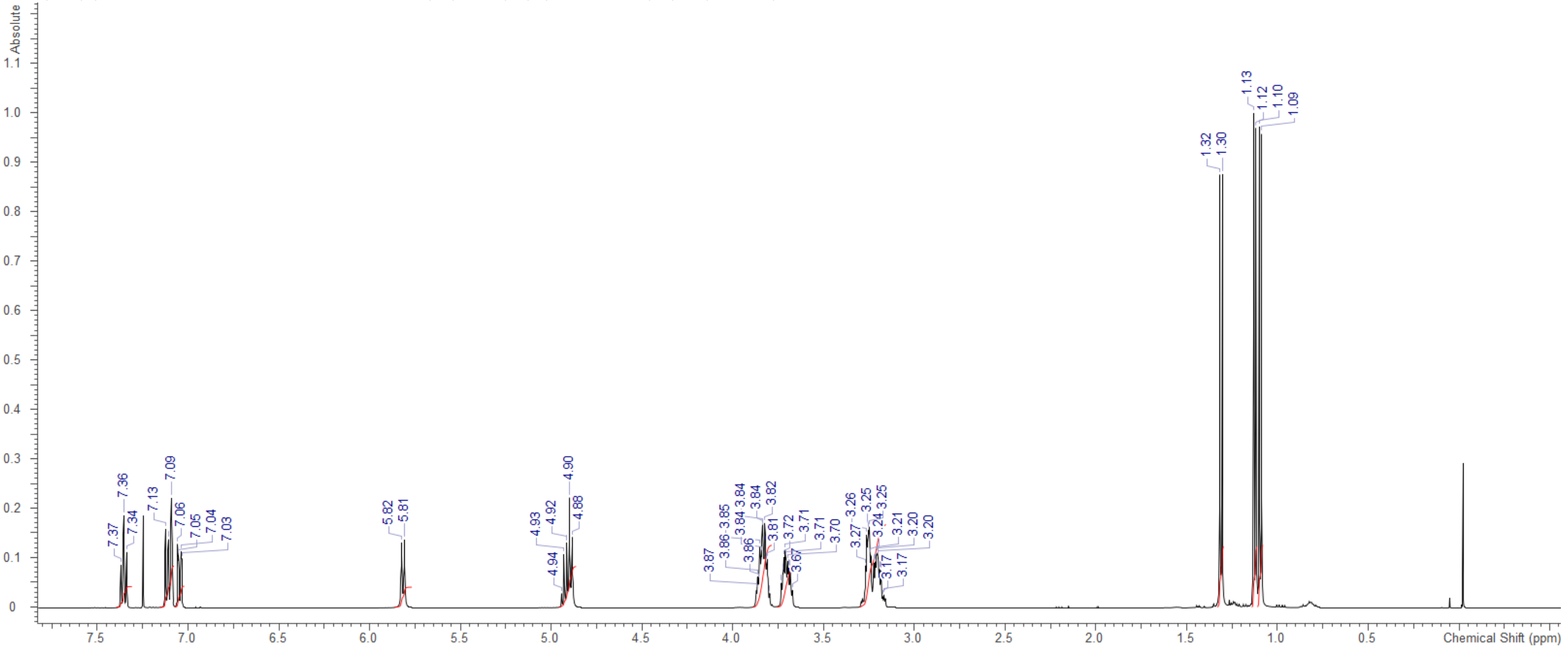

1-isopropyl-3-(1-oxo-1-(4-(3-(trifluoromethyl)phenyl)piperazin-1-yl)propan-2-yl)urea (**36**) – ^{13}C NMR

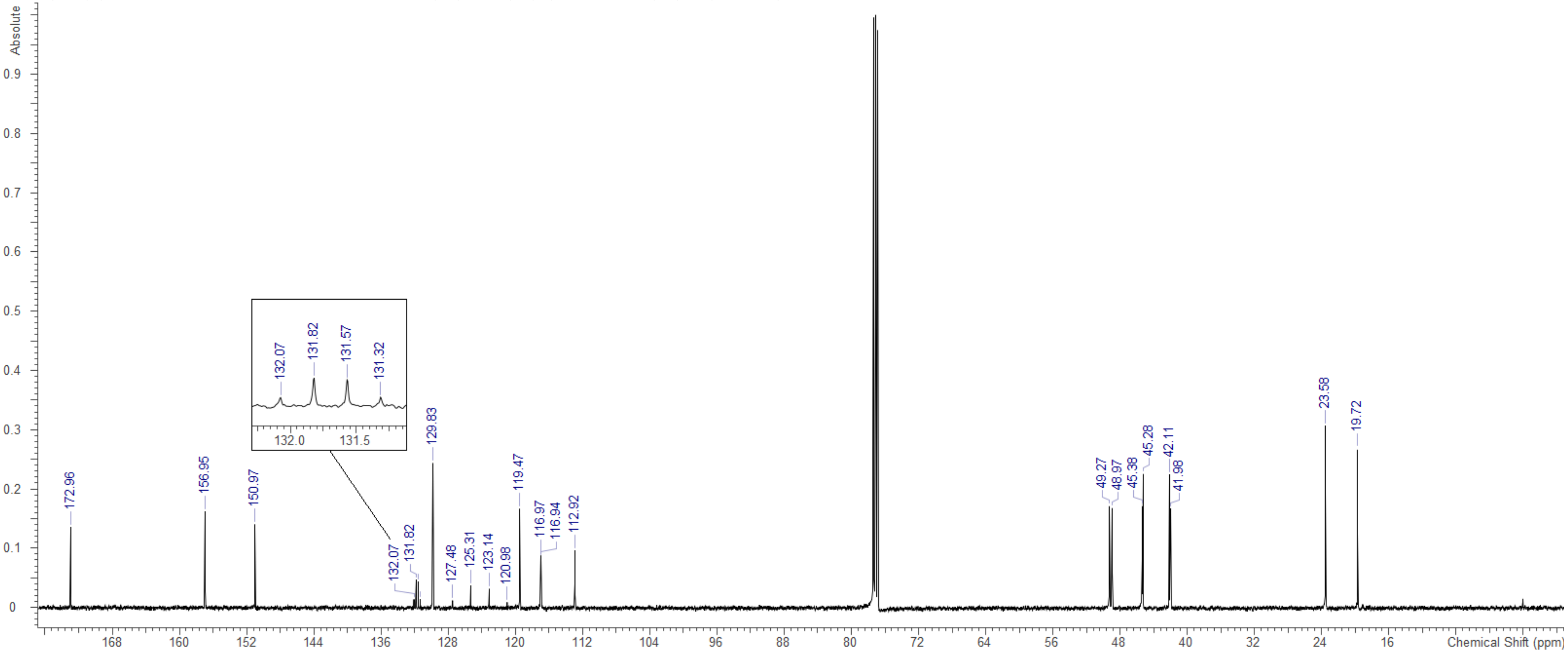

2-acetamido-N-(1-phenylpyrrolidin-3-yl)propanamide (**45**) – ^{1}H NMR

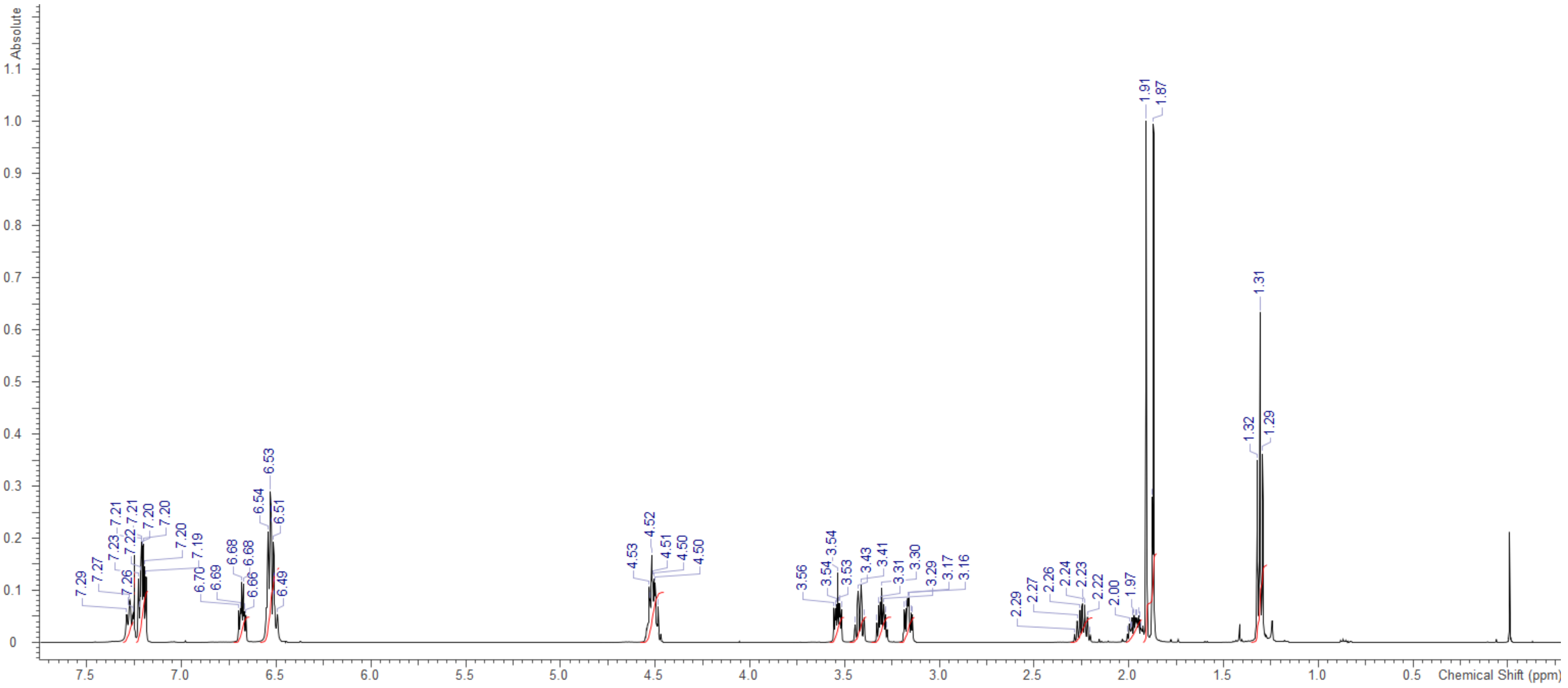

2-acetamido-N-(1-phenylpyrrolidin-3-yl)propanamide (**45**) – ^{13}C NMR

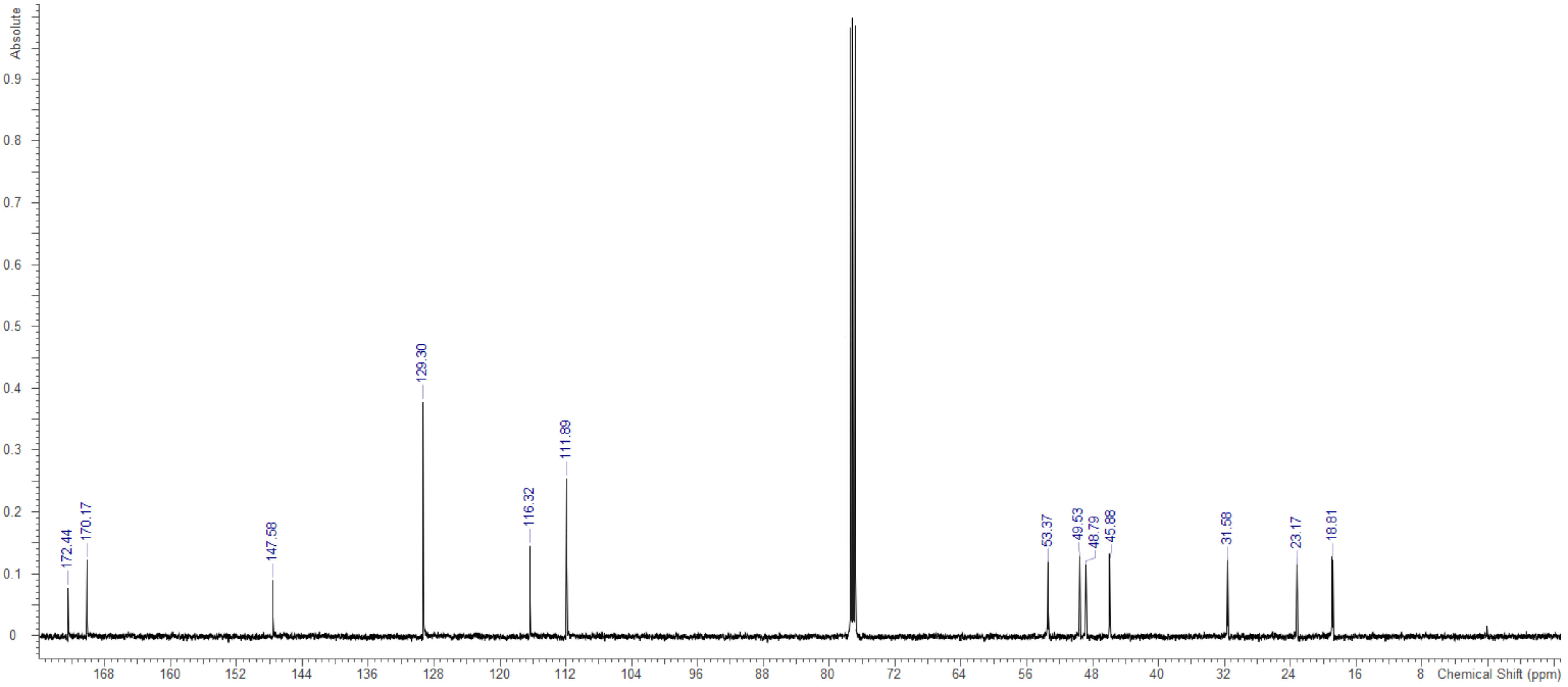

2-acetamido-N-(1-(3-(trifluoromethyl)phenyl)pyrrolidin-3-yl)propanamide (**46**) – ^{1}H NMR

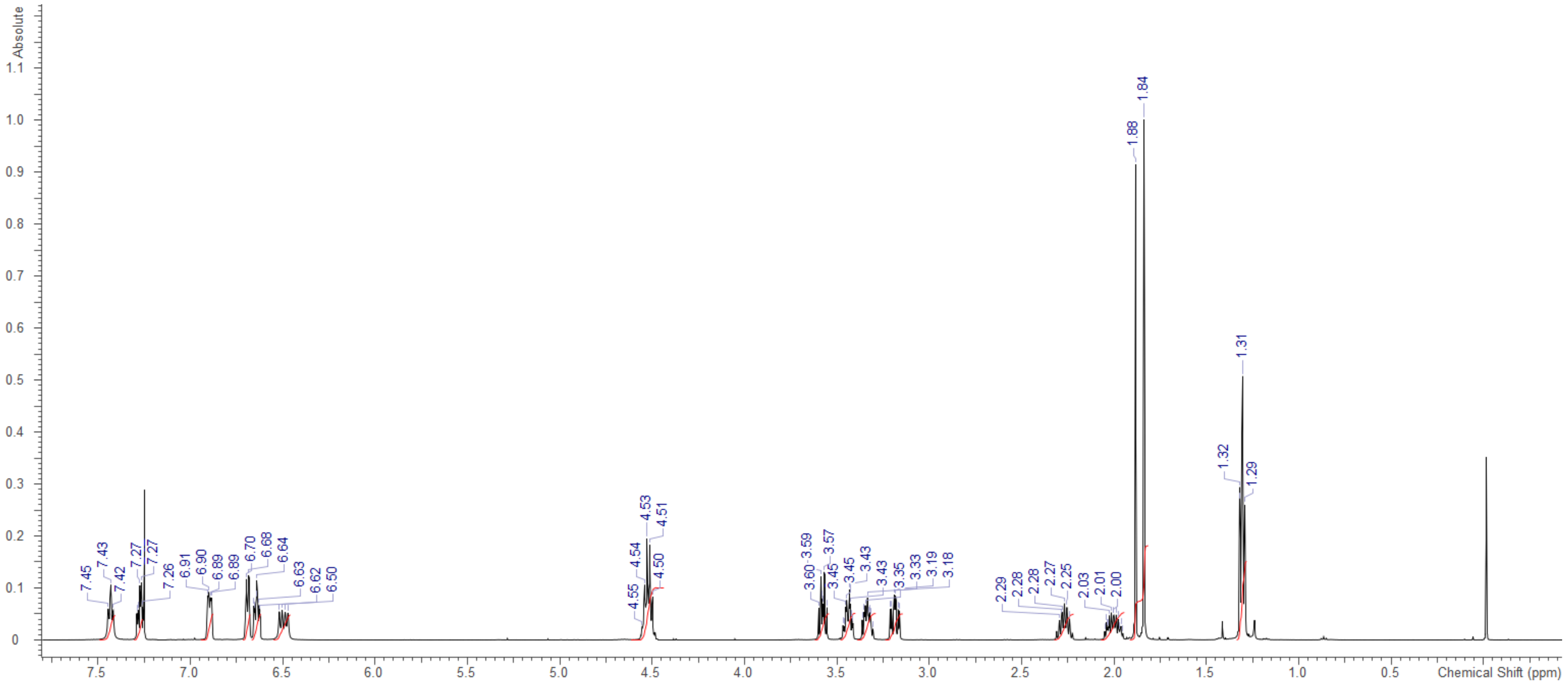

2-acetamido-N-(1-(3-(trifluoromethyl)phenyl)pyrrolidin-3-yl)propanamide (**46**) – ^{13}C NMR

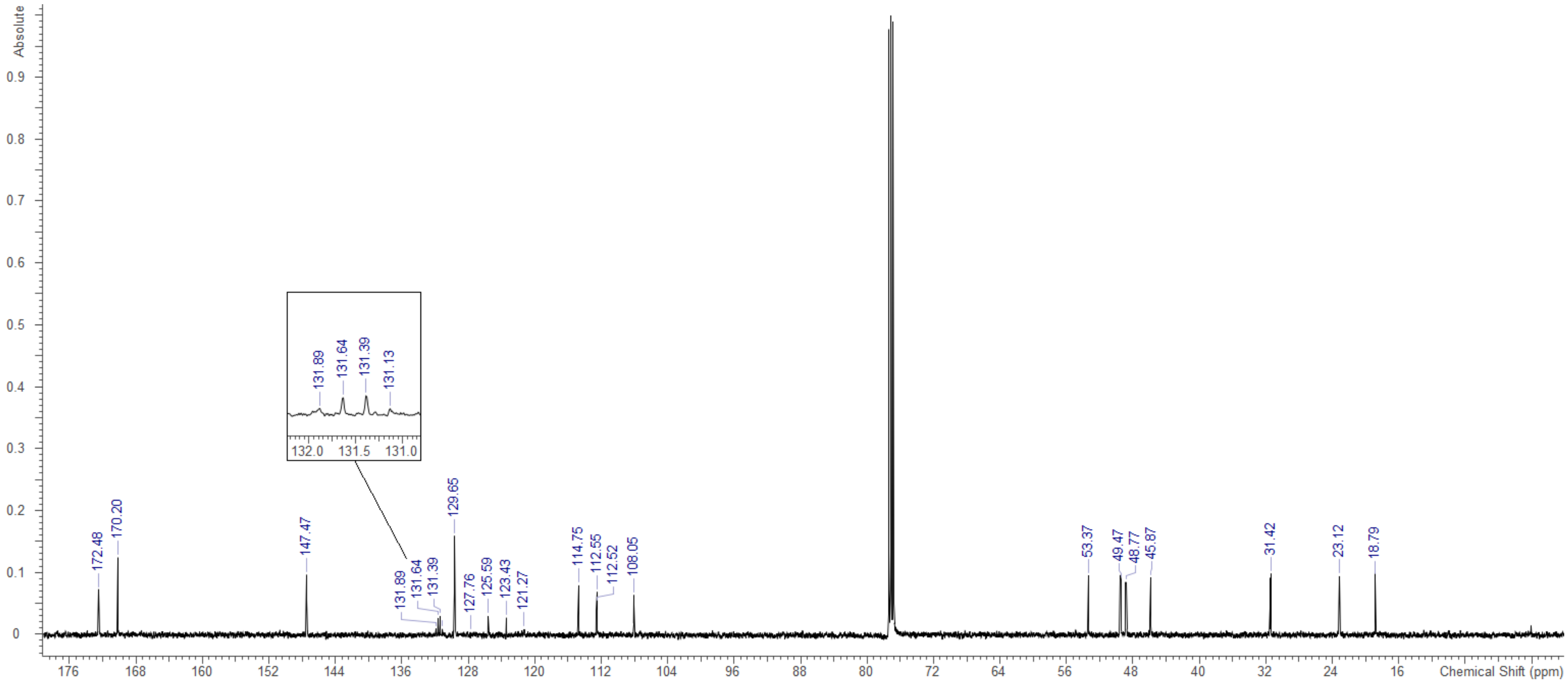

2-acetamido-N-(1-(3-(trifluoromethoxy)phenyl)pyrrolidin-3-yl)propanamide (**47**) – ^{1}H NMR

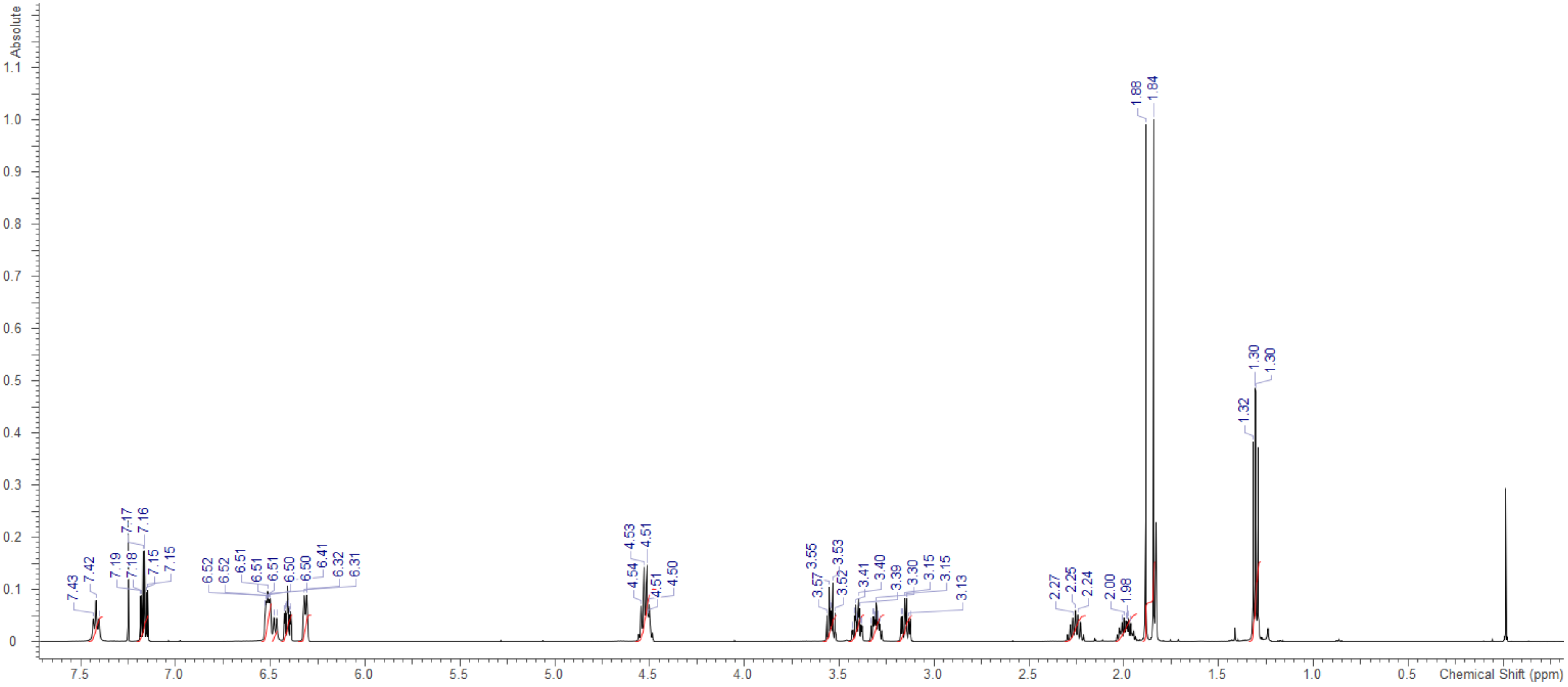

2-acetamido-N-(1-(3-(trifluoromethoxy)phenyl)pyrrolidin-3-yl)propanamide (**47**) – ^{13}C NMR

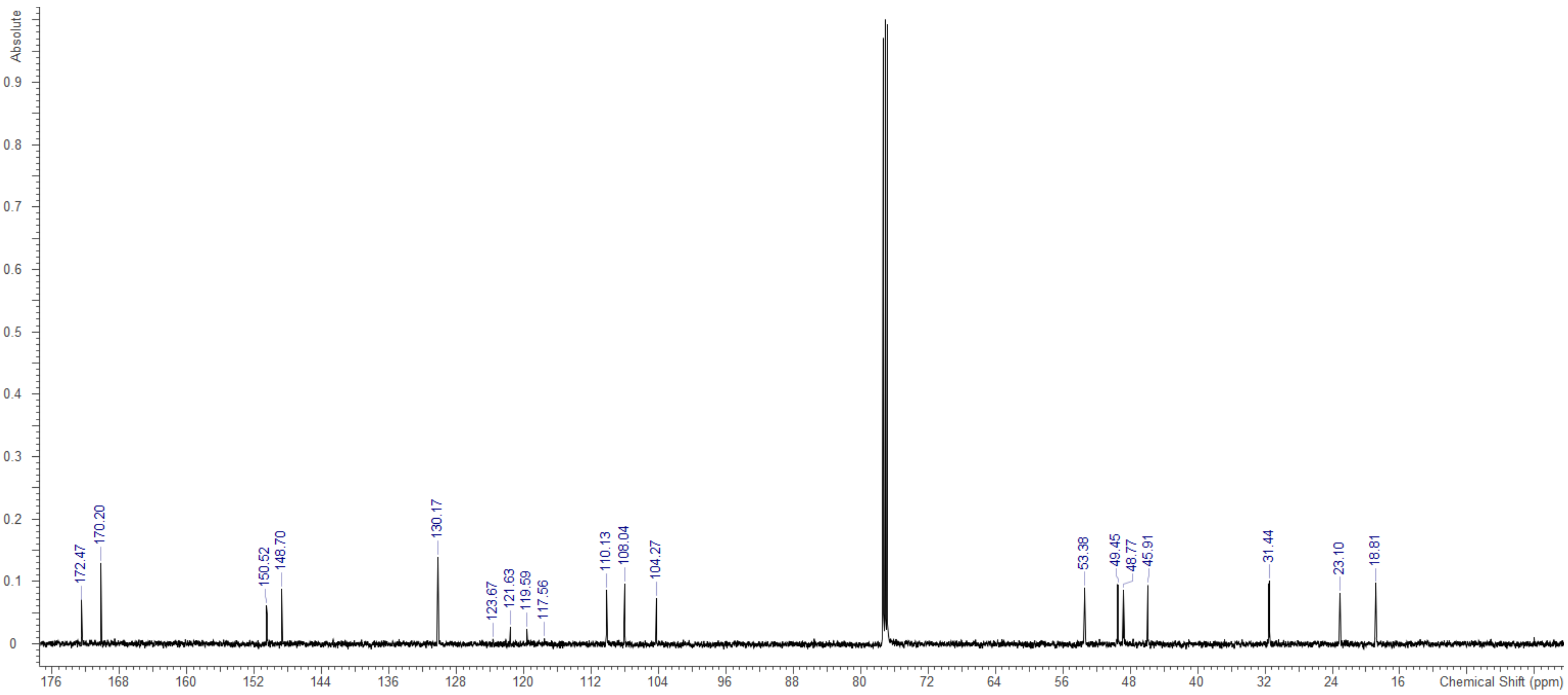
Absolute
0.9
0.8
0.7
0.6
0.5
0.4
0.3
0.2
0.1
0
172.47
170.20
150.52
148.70
130.17
123.67
121.63
119.59
117.56
110.13
108.04
104.27
53.38
49.45
48.77
45.91
31.44
23.10
18.81
176
168
160
152
144
136
128
120
112
104
96
88
80
72
64
56
48
40
32
24
16
Chemical Shift (ppm)

2-acetamido-N-(1-(3-((trifluoromethyl)thio)phenyl)pyrrolidin-3-yl)propanamide (**48**) – ^{1}H NMR

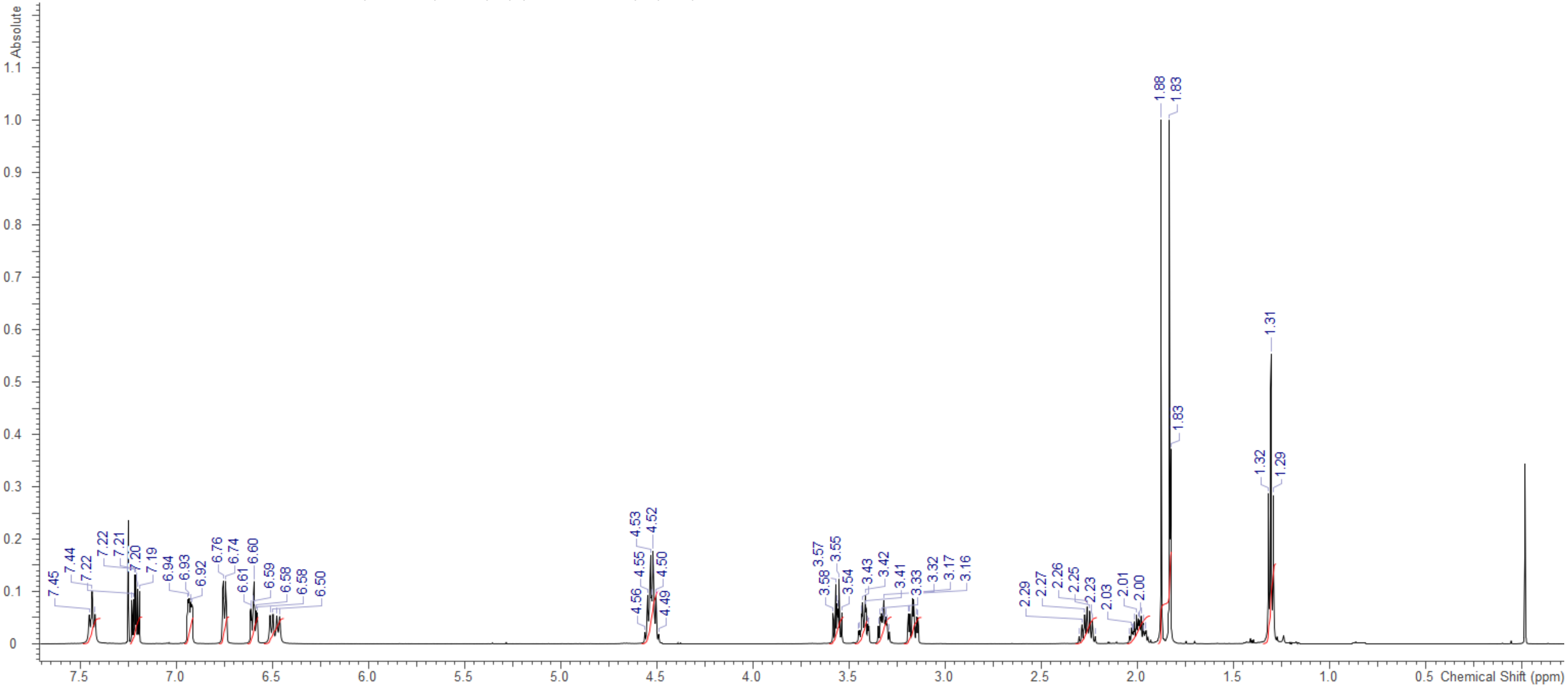

2-acetamido-N-(1-(3-((trifluoromethyl)thio)phenyl)pyrrolidin-3-yl)propanamide (**48**) – ^{13}C NMR

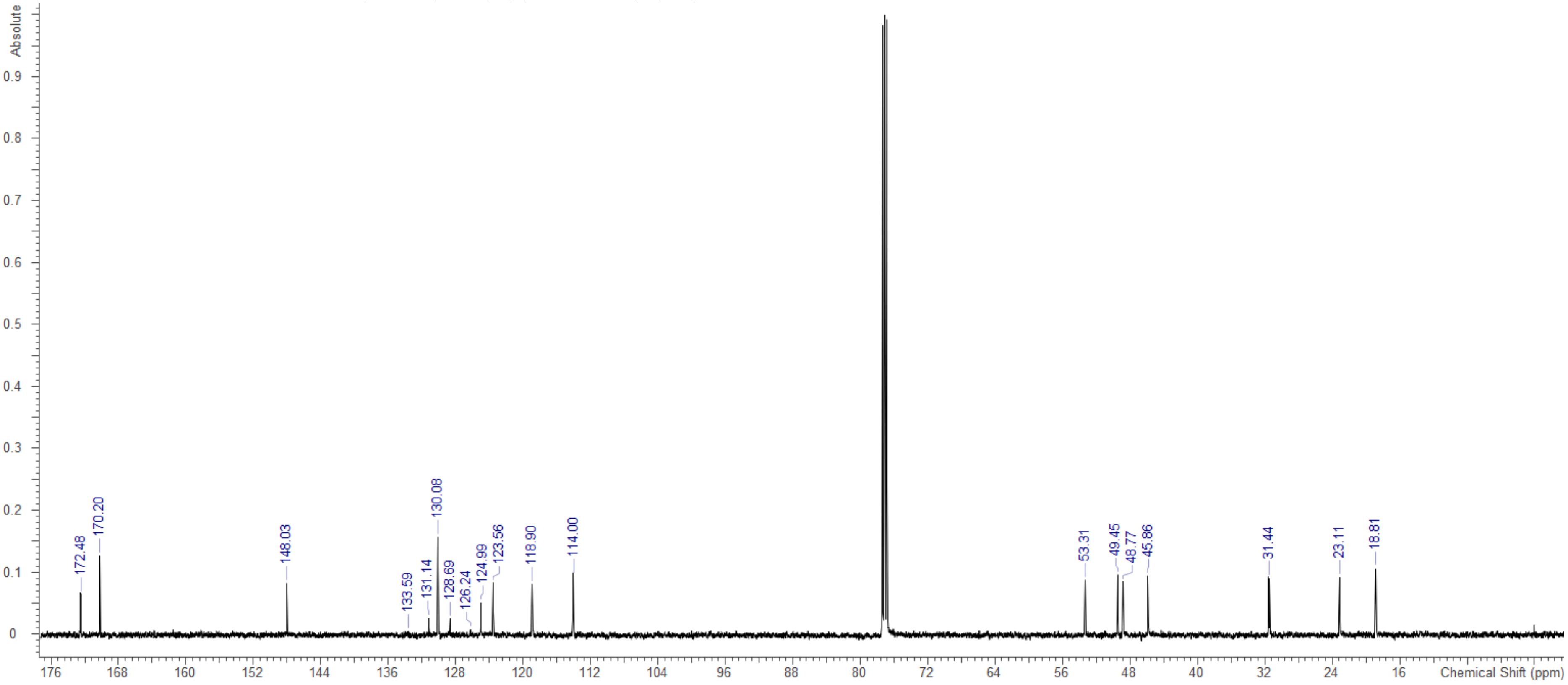

Supplement: Supplementary file 1 — cn4c00013_si_001.pdf [file cn4c00013_si_001.pdf]
